# Impact of sex and APOE-*ε*4 genotype on patterns of regional brain atrophy in Alzheimer's disease and healthy aging

**DOI:** 10.3389/fneur.2023.1161527

**Published:** 2023-06-02

**Authors:** Benoît Sauty, Stanley Durrleman

**Affiliations:** Sorbonne Université, Institut du Cerveau-Paris Brain Institute–ICM, CNRS, Inria, Inserm, AP-HP, Hôpital de la Pitié Salpêtrière, Paris, France

**Keywords:** Alzheimer's disease, longitudinal studies, brain atrophy, genetic covariates, sexual dimorphism, aging

## Abstract

Alzheimer's Disease (AD) is a heterogeneous disease that disproportionately affects women and people with the APOE-*ε*4 susceptibility gene. We aim to describe the not-well-understood influence of both risk factors on the dynamics of brain atrophy in AD and healthy aging. Regional cortical thinning and brain atrophy were modeled over time using non-linear mixed-effect models and the FreeSurfer software with t1-MRI scans from the Alzheimer's Disease Neuroimaging Initiative (*N* = 1,502 subjects, 6,728 images in total). Covariance analysis was used to disentangle the effect of sex and APOE genotype on the regional onset age and pace of atrophy, while correcting for educational level. A map of the regions mostly affected by neurodegeneration is provided. Results were confirmed on gray matter density data from the SPM software. Women experience faster atrophic rates in the temporal, frontal, parietal lobes and limbic system and earlier onset in the amygdalas, but slightly later onset in the postcentral and cingulate gyri as well as all regions of the basal ganglia and thalamus. APOE-*ε*4 genotypes leads to earlier and faster atrophy in the temporal, frontal, parietal lobes, and limbic system in AD patients, but not in healthy patients. Higher education was found to slightly delay atrophy in healthy patients, but not for AD patients. A cohort of amyloid positive patients with MCI showed a similar impact of sex as in the healthy cohort, while APOE-*ε*4 showed similar associations as in the AD cohort. Female sex is as strong a risk factor for AD as APOE−ε4 genotype regarding neurodegeneration. Women experience a sharper atrophy in the later stages of the disease, although not a significantly earlier onset. These findings may have important implications for the development of targeted intervention.

## 1. Introduction

Alzheimer's Disease (AD) is a neurodegenerative pathology that accounts for about 70% of the more than 55 millions dementia cases worldwide (1). A silent phase, referred to as prodromal phase, shows accumulation of pathological proteins in the brain that lead to structural alterations in the brain without specific symptoms, eventually leading to a progressive cognitive decline that translates into a loss of the patient's autonomy for daily tasks, with impaired mnesic, visuospatial, and communicative functions as well as unusual behaviors such as paranoia and aggressiveness. For people under the age of 75, the incidence of AD has been estimated below 1.0%, while it reaches 8.4% (2, 3) for patients over 85, making older age the most important risk factor of the disease. Other risk factors have been identified with certainty and are briefly presented below.

The second most important risk factor is often said to be the presence of hereditary gene mutations (4). More precisely, the inheritance of AD can be of two types. On one hand, rare autosomal dominant mutations in APP, PSEN1, and PSEN2, that encode amyloid precursors and presenilin proteins, leading to early-onset (<60 years old) familial AD, which accounts for less than 1% of cases (1), and are often dismissed from studies on risk factors because of their low prevalence and understood causality. On the other hand, a frequent gene polymorphism: the ε4 variant of the APOE gene, can influence susceptibility for roughly 50% of the common late-onset AD (4). Genome wide association studies also identified a handful of other loci as potential risk factors, but thus far, the only gene variant considered to be an established risk factor for late onset AD is the APOE−ε4 allele (1, 5, 6). The APOE gene provides the blueprint for the alipoprotein E that transports cholesterol in the bloodstream and helps bind Aβ to cerebrospinal fluid, effectively clearing the brain of excess Aβ. The presence of ε4 allele hinders Aβ clearance and leads to reduced neuronal injury response (7), and in turns correlate with higher AD risks.

The other genetic factor that is known to impact AD risk rates is the sex of patients. Two thirds of AD patients are women (1) and their cognitive decline is faster than for men (8). By contrast, healthy aging tends to display delayed cognitive decline and structural alterations for women. Beam et al. (9) review conflicting studies on the topic of incidence rates and reach the conclusion that before the age of 75, no significant difference in incidence rates is detected, while incidence rates get significantly higher for women than for men after that inflection point. The longer longevity of women has long been thought to be the sole cause for this discrepancy. However, the loss of the protective effects of estrogens during menopause, demonstrated *in vitro* and *in vivo* (10, 11), has also been suggested as a cause. Evidence also highlight a faster loss of autonomy but a longer lifespan after diagnosis for women (12). These facts raise the question of a sexual dimorphism of AD. A precise understanding of the distinct patterns of structural and functional brain alterations over time is still lacking. Accurate descriptions of those patterns could help to implement better practices to care for both men and women. In addition, women are still under-represented in clinical trials (13) and only 15% of trials report results stratified by sex (14), although this proportion increases in more recent studies. For instance, the recent trials for Lecanemab reported a better response to treatment for men, and featured just over 50% women in both arms (15). On the other hand, trials for Donanemab (16) and Aducanumab (17) display similar enrollment demographics, but do not report results by sex. As suggested in Mielke (18), several gendered factors other than sex can also mediate the observed associations, although data is rarely available in large scales datasets.

Another often-mentioned risk factor is the education level. According to the cognitive reserve hypothesis, having more years of education increases the connections between neurons and enables the brain to compensate for the early changes of Alzheimer's by using alternate routes of neuron-to-neuron communication to complete a cognitive task (19, 20). Women are believed to benefit even more from higher education than men, albeit receiving less education (21). Mungas et al. (22) confirm that education is an indicator of cognitive reserve, but that the protective effects on cognition are depleted as neurodegeneration progresses.

Building on the observation that the influence of sex on the progression of AD is not well understood, and that APOE-*ε*4 status is the strongest known genetic variant associated with AD, this work aims to describe the disentangled contributions of both those genetic factors on patterns of structural brain alterations, while accounting for biases due to varying education level. Such alterations, that are visible through MRI scans, reveal neurodegeneration occurring before the onset of the cognitive symptoms, and are thus crucial to understanding the early stages of the disease and for the design of drug trials. Leveraging the information from repeated measurements of cortical thicknesses and volumes of subcortical regions allows describing each patient's atrophic dynamics and compare across populations. Our goal is thus to provide a map of the brain regions that show significant correlation between sex or APOE-*ε*4 status and onset age and pace of regional atrophy. We provide this analysis for AD diagnosed and cognitively normal patients in order to emphasize the specificities of Alzheimer's progression.

### 1.1. Related work

#### 1.1.1. Cross-sectional studies

Over the course of AD progression, the link between cognitive decline and various covariates such as body mass index (21), cardiac pathologies, APOE-*ε*4 genotype (23–26) and sex have been explored, highlighting a higher impact of comorbidities and APOE-*ε*4 genotype for women on the severity of the disease symptoms. Ferretti et al. (27) and Laws et al. (28) review the state of knowledge about the differences across sexes in AD clinical manifestations, biomarkers patterns and risk factors.

On the other hand, healthy aging displays comparatively more spared alteration profiles for women compared to men, for both brain atrophy and cognitive decline. Seminal works dating back to the beginning of *in-vivo* imaging modalities report higher size decreases in men for average brain (29) and frontal and temporal lobes (30). Many studies have since provided more insights into the differences between sexes for cognitive decline and link with comorbidities (31, 32), exhibiting opposite correlation than in Alzheimer's studies. Jack et al. (33) exhibit associations between male sex, worse memory, and higher hippocampal atrophy.

#### 1.1.2. Longitudinal studies

The collection of repeated measurements of neuroimaging data and clinical assessment allows a finer understanding of pathological pathways. Well-established modeling approaches include event-based models (34, 35), Gaussian-Process models (36, 37), Deep Learning methods (38, 39) and mixed-effects models. Application of such longitudinal models are frequent for the assessment of cognitive decline (34, 40–46) but less used for imaging features. Bernal-Rusiel et al. (47) advocate the use of linear mixed-effects models (LME) for progression models of neuroimaging data, and apply LME to a mesh of control points of cortical thickness in a mass-univariate setting in (48). Sabuncu et al. (49) correlate these progression models with diagnosis in order to exhibit regions of the cortex that are most representative of the cognitive state of patients. Other studies also apply progression models to a few ROIs of the brain (50–53) in order to display the regions that correlate most with diagnosis. Applying this kind of modeling to a study of sex differences, (54, 55) find that atrophy rates are higher for female than male patients, while (56, 57) explain that brain volumes and cortical thicknesses are consistently smaller for cognitively normal males after accounting for age, and that the differences get smaller as cohorts are chosen at a more advanced state of cognitive decline, until they get indistinguishable for confirmed AD patients, with a sharper decline for women. Several studies also find that sex modulates the interactions between amyloid SUVR and brain volume change (32), between CSF biomarkers and hippocampal atrophy (58) and between APOE genotype and hippocampal atrophy (59).

Those studies do not take into account important covariates such as APOE status and educational level, and do not disentangle the age of onset and the pace of atrophy. Many studies report the impact of APOE polymorphisms on brain atrophy in AD progression and link ε4 with faster cortical thinning (60–62) and hippocampal atrophy (62–65), with increasing effects as patients get older (66). In the context of non-demented aging, only ε4 homozygotes are found to increase hippocampal and total gray matter atrophy (67), with a stronger correlation between hippocampal volume and memory loss for ε4 carriers (68). GWAS also highlight APOE as the genetic variant mostly associated with brain atrophy and cortical thinning across lifespan, with a stronger effect for subjects with brain disorders (69).

### 1.2. Contributions

Overall, many studies explored the differences between men and women regarding AD progression and healthy brain aging, as well as the influence of APOE polymorphisms. However, many of them rely on cross-sectional data that miss important information about the progression of the disease. Longitudinal tools have been proposed to leverage such information, and have been successfully applied to biomarkers for disease progression models. However, these studies have either focused on the patterns of cognitive decline, or on a chosen set of ROIs to assess structural alterations, thus not providing a detailed map of the differences across the brain. Some longitudinal studies have proposed analysis of cortical thinning or brain atrophy with a high spatial resolution, however the focus has always been put on finding regions that correlate best with diagnosis in order to obtain valuable information about the brain alterations most specific to AD. To the best of our knowledge, no study has yet provided a quantitative comparison between male and female patterns of structural alterations over time, while accounting for APOE-*ε*4 genotype and education level, in order to provide a map of the brain regions that showcase distinct age-related alterations profiles. In addition, former works usually focus on the impact of one single factor, while only a joint analysis of both the genetic factors allows isolating the contributions from each one.

In this context, it seems relevant to further explore the differences between men and women regarding the onset and pace of alterations of the brain in both normal and AD progression, with a high degree of spatial resolution, while accounting for APOE-*ε*4 genotype and education level. Longitudinal studies allow to leverage information about the progression over time, while mixed-effect models allow for an interpretable modeling of each feature's trajectory across time. To sum up our contributions:

- We model regional cortical thinning and brain atrophy using non-linear mixed-effect models, and propose a formulation that disentangles the onset age and the pace of atrophy,
- for each region, we proceed to a multivariate regression for the individual parameters with regard to female sex, APOE-*ε*4 allele count and education level, in order to isolate the effect of each factor,
- we obtain a brain map of *p*-values that assess how significantly each factor influences the patterns of atrophy over time, when corrected for the other covariates,
- we display the regions that differ the most regarding onset age and pace of structural atrophy for the chosen covariate, for both healthy and AD progression.

This statistical pipeline addresses the lack of understanding of the influence of sex and APOE on structural brain alterations, and disentangles the joint effects of the multiple risk factors.

## 2. Materials and methods

### 2.1. Data sets

We performed the analysis on publicly available data from all waves (GO,1,2,3) of the Alzheimer's Disease Neuroimaging Initiative (ADNI) database (adni.loni.usc.edu), as it provides repeated MRI scans for both cognitively normal elderlies and AD patients. The ADNI was launched in 2003 as a public-private partnership and its primary goal has been to test whether serial magnetic resonance imaging (MRI), positron emission tomography (PET), other biological markers, and clinical and neuropsychological assessment can be combined to measure the progression of mild cognitive impairment (MCI) and early Alzheimer's disease (AD). All scans are quality-checked by trained anatomists. We selected all patients with at least 2 visits with a MRI scan and selected three cohorts : an AD cohort with patients with at least one confirmed AD diagnosis, a healthy cohort with patients who are diagnosed cognitively normal at every visit and an intermediate cohort of amyloid positive patients (see Hansson et al. (70) for the chosen cutoffs) with at least one MCI diagnosis but no AD conversion. Patients with inconsistent AD diagnosis that revert to CN or MCI (107 patients) and amyloid negative patients (50 patients) are removed from the pathological cohort. Table 1 reports the demographics for the selected ADNI patients. The AD cohort is composed mostly of late-onset AD (566 patients) but also features a few early-onset cases (24 patients).

**Table 1:** Demographics for cohorts selected from ADNI.

	**AD cohort**		**A**β**+ MCI cohort**		**Healthy cohort**	
	**Male**	**Female**	**Male**	**Female**	**Male**	**Female**
Patients (*N*)	329	261	262	176	210	264
Visits (*N*_*scan*_)	1,447	1,092	1,164	794	1,063	1,169
Total follow-up (y)	3.7 ± 2.9	3.4 ± 3.0	4.5 ± 3.4	4.6 ± 3.3	4.6 ± 3.3	4.1 ± 3.0
Age at baseline	75.0 ± 7.1	73.6 ± 7.7	75.4 ± 7.5	71.5 ± 7.8	74.0 ± 6.1	72.7 ± 6.0
Education (y)	16.1 ± 2.8	14.7 ± 2.6	16.4 ± 2.8	15.5 ± 2.7	17.2 ± 2.4	16.1 ± 2.8
APOE-*ε*4 (2/1/0)	69/163/97	44/133/84	32/99/121	24/71/81	5/51/154	7/75/182
MMSE	24.0 ± 4.0	23.2 ± 4.6	27.7 ± 2.0	28.2 ± 2.1	29.0 ± 1.2	29.2 ± 1.1
ADAS-Cog13	26.5 ± 10.6	29.2 ± 11.8	15.3 ± 6.9	12.6 ± 6.7	10.0 ± 4.6	8.3 ± 4.3

Numeric fields are in the form mean ± standard deviation. (y) is years. (2/1/0) refers to the amount of APOE-*ε*4 alleles. Cognitive scores are for all visits (not just baseline).

### 2.2. Data processing

In order to validate our findings with an independent measure of atrophy, we extract gray matter density maps from the same t1-MRI data from ADNI. These features are obtained using the t1-volume pipeline of Clinica (71). This pipeline is a wrapper of the Segmentation, Run Dartel (using a ADNI-based template) and Normalize to MNI Space routines implemented in the SPM12 software and yields a map of the average gray matter densities in each anatomical region defined in the AAL2 atlas (72), which splits the brain into 80 gyri and sulci for the cortex and 14 subcortical regions. It displays a similar granularity to the Destrieux atlas and allows comparison. Gray matter density is fundamentally different from cortical thicknesses and regional volumes, and could potentially exhibit different progression patterns, especially since it is well documented that women have on average more gray matter and less white matter than men after correcting for brain size, in most of the brain (73–75). Each scan is processed independently, using the cross-sectional routines, since the amount and temporal spacing between successive scans is very variable. Indeed, the longitudinal routines build subject-specific templates to reduce within-subject variability, and are particularly useful when the successive visits are acquired at the same timepoint for all patients. In our case, the longitudinal templates would not be built the same way for each patient, and using the cross-sectional processing tools ensures that the thicknesses and volumes are estimated the same way for everyone, even when visits are missing.

MRI images from ADNI are acquired with either 1.5T (3,440 samples) or 3T (3,289 samples) scanners. As noted in Han et al. (76), Freesurfer outputs on 3T images are significantly thicker than for 1.5T images, which can hurt longitudinal modeling and bias population-level comparisons. As is common in longitudinal studies, we proceed to additive bias correction within each diagnosis group for each extracted feature. The corresponding matched histograms are presented in Supplementary Figures 7, 8.

### 2.3. Longitudinal modeling

**Figure 1:**
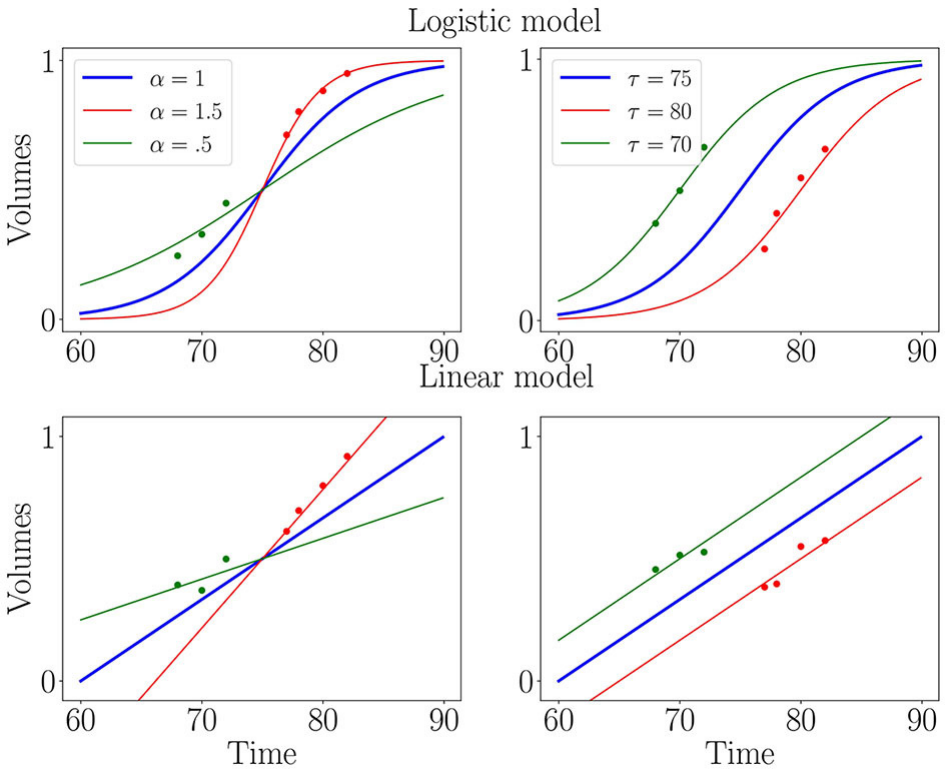
Illustration of logistic (first row) and linear (second row) mixed-effects models. Blue curve is the average trajectory (parametrized by *t*_0_, *p*_0_ and *v*_0_) and red and green curves illustrate the effect of progression pace (α = *e*^ξ^) and onset age (τ), which allow matching individual measurements (isolated dots) to a continuous trajectory.

Mixed-effects models describe each patient's progression over time as a small variation—the random effects of the model—around the average population trajectory—the fixed effects of the model. This study focuses on univariate models, that are calibrated on the repeated measurements for one region of the cortex or subcortical structure at a time. Linear mixed-effects models are widely used for longitudinal modeling and assume that features evolve according to straight lines over time. Each patient is thus characterized by a slope and an intercept. The slope can be interpreted as the pace of decline of a patient for the given feature, but the intercept is less meaningful. In that setting, we choose to parametrize the inter-subjects variability as the combined effects of an individual onset age, and a pace of decline as suggested in Schiratti et al. (44). The onset age can be understood as a horizontal intercept and describes the age at which a patient crosses the population-average-value threshold, and the pace of decline as the slope. Both these temporal parameters allow to align all patients on a common progression timeline. Namely, given a family of feature observations {*y*_*i,j*_} at times {*t*_*i,j*_} for 1 ≤ *i* ≤ *N* indexes the N patients and 1 ≤ *j* ≤ *N*_*i*_ indexes the *N*_*i*_ visits of patient *i*, we resort to the mixed-effect generative model


$$y_{i,j}=f\left(e^{\xi_{i}}\left(t_{i,j}-\tau_{i}\right)+t_{0}\right)+\varepsilon_{i,j}$$

where $e^{\xi_{i}}$ and τ_*i*_, respectively, the *progression pace* and *onset age* of patient *i* are called the individual parameters that provide an affine time warp to account for the variability in pace and onset of decline between patients. These individual parameters form the random effects of the model. We choose the Gaussian priors for the noise $\varepsilon_{i,j}\sim N\left(0,\sigma_{\varepsilon }^{2}\right)$ and random effects $\tau_{i}\sim N\left(t_{0},\sigma_{\tau }^{2}\right)$ and $\xi_{i}\sim \;N\left(0,\sigma_{\xi }^{2}\right)$. In a linear mixed-effects framework, *f* is an affine function *t*↦*p*_0_+*v*_0_*(*t*−*t*_0_), while for non-linear mixed-effects it is usually chosen to be the logistic function $t\mapsto {\left(1+\left(\frac{1}{p_{0}}\right)\exp\left(\frac{-v_{0}t}{p_{0}\left(1-p_{0}\right)}\right)\right)}^{-1}$ as illustrated in Figure 1. The parameters *p*_0_, *v*_0_ and *t*_0_ are respectively a reference *position, velocity* and *time* and describe the average trajectory. Together with the variances σ_ε_, σ_τ_, σ_ξ_, they form the fixed-effects of the model.

In practice, logistic regression is one of the most popular shape of trajectory, so all features should be normalized to the range [0, 1]. We thus discard outliers using the so-called three sigma rule and add a min-max normalization within each cohort, providing realistic asymptotes. Since cortical thicknesses and brain volumes decrease over time, we flip data around .5, using the rotation *x*↦1−*x*, in order to ensure increasingness, which is required for logistic modeling.

### 2.4. Calibration of the model

These longitudinal statistical models are part of a family of geometric models that have been studied in Koval et al. (45) and Schiratti et al. (44). We can proceed to a Maximum a Posteriori estimation of both random and fixed effects with the MCMC-SAEM procedure, in which the estimation step of an Expectation-Maximization algorithm is replaced by a stochastic approximation. See Kuhn et al. (77) and Allassonniere et al. (78) for details on this procedure, description of the complete likelihood, and proof of convergence and stability. One model is calibrated on each of the three cohorts. All analysis is done using the Leaspy software.

### 2.5. Logistic vs. linear regression

As mentioned above, the choice of *f* in the mixed-effect formulation should reflect the average progression of the feature. A few methods exist to learn the exact shape of the progression profile (79, 80) but add a strong computational burden. We compared the two most widespread available shapes, namely linear, and logistic curves, in order to assess the stability of convergence as well as the estimated noise of the longitudinal fit. The logistic curve marginally surpassed the linear curve for both these criteria.

### 2.6. Goodness of fit

In order to ensure that the longitudinal models accurately describe the atrophy of each feature, we compare the distributions of reconstruction errors (the differences between the predicted values of the models at the time of each visit and the actual measurements) for the three cohorts to the distribution of measurements noise. Imaging data are indeed subject to variations in the experimental conditions and in the processing pipeline. In order to evaluate this noise, some visits are acquired twice within a few hours. For ADNI, we dispose of 1604 duplicate images from 285 different patients. We provide boxplots that display the empirical distributions of test/re-test errors and of the reconstruction errors for the three cohorts in Supplementary material. Distributions largely overlap, and reconstruction errors fall close to the measurements' uncertainty, which hints that our models could not be improved without overfitting. Additionally, it is important to note that the reconstruction errors are not biased regarding sex, APOE status, education level, ADNI phases nor field strength.

### 2.7. Statistical analysis

**Figure 2:**
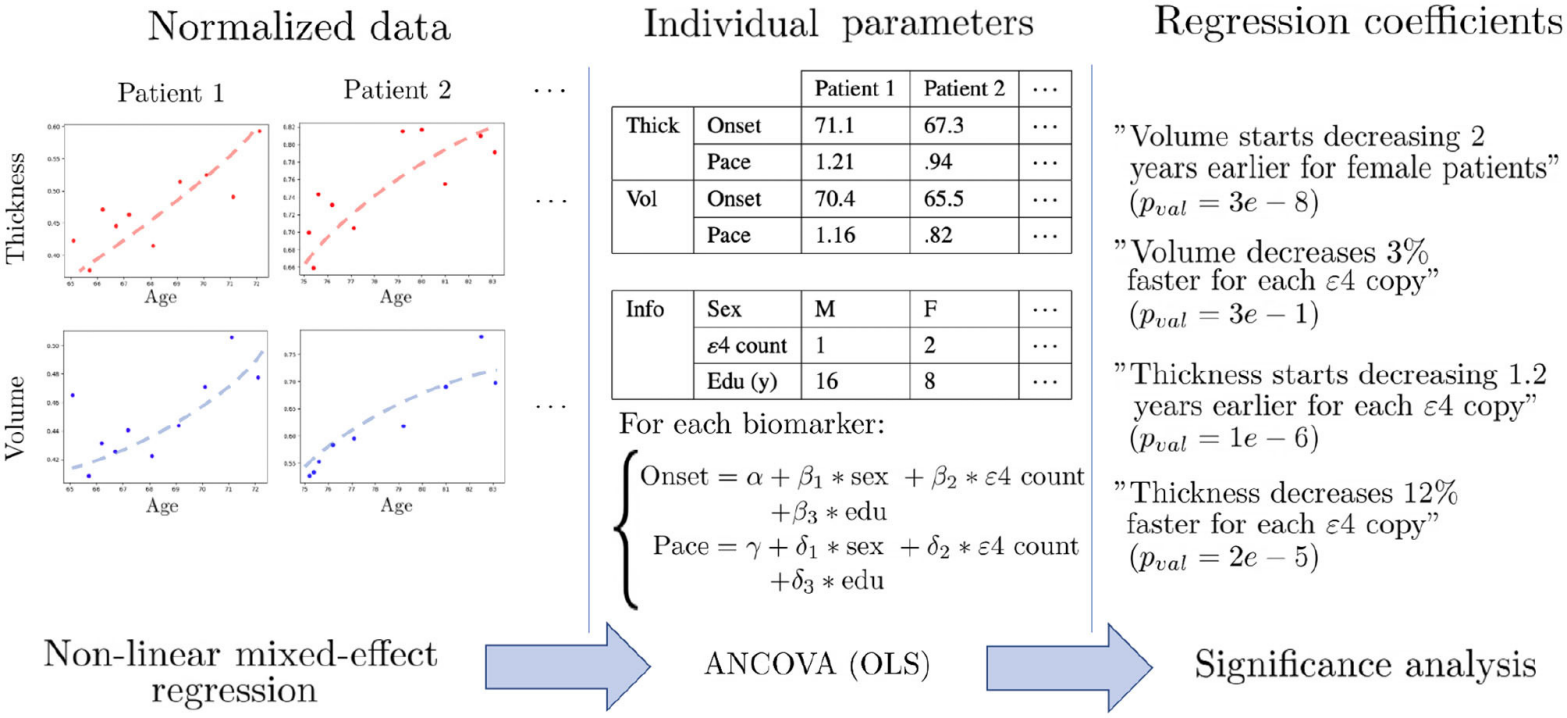
Schematic description of the statistical pipeline for two dummy features called “Volume” and “Thickness”. First, the normalized data is modeled by univariate non-linear mixed-effects models, in order to obtain an individual age at onset and a pace of decline for each feature. The dots are the actual measurements over time, and the dashed shapes are the fitted trajectories. Then, covariance analysis is performed using ordinary least square regression (OLS) in order to learn the influence of each covariate on the onset and pace of decline. Finally, statistical analysis is performed in order to assess the significance of each association.

Once the univariate models are calibrated, we have a family of patient-wise onset ages and progression paces for each feature. We can thus proceed to statistical testing of the differences between subgroups. To do so, we perform covariance analysis using ordinary least squares to regress the individual parameters with regard to female sex, number of APOE-*ε*4 alleles and education level of each patient. This evaluates the impact of each covariate after correcting for the other ones, and also the significance of the corresponding association. Since we are in a multiple testing setting, we use False Discovery Rate (FDR) (81) to obtain a map of corrected *p*-values that assess how certain it is that the chosen covariate influences the onset or pace of atrophy for each region. Figure 2 summarizes the analysis pipeline. Given the high number of statistical tests (one for each of the 148 regions of the Destrieux atlas), a more stringent correction method such as Family-Wise Error Rates would be unsuitable as it would discard significant correlations. This *a posteriori* analysis is necessary in our non-linear Bayesian setting, since the covariates can not be integrated in the longitudinal model as they would for a general linear model. Mixture models have been proposed in Poulet et al. (82) in order to account for covariates in the distributions of the random-effects, but it requires a lot more data in order to estimate such distributions for each combination of covariates.

### 2.8. Interpretability of the *p*-values

For each feature, patients are aligned on a common timeline with the individual onset age and pace of atrophy. One can, for instance, observe that for the inferior temporal gyrus, cortical thinning occurs on average 1.5 years earlier for each copy of the ε4 allele, and on average 23% faster for women, after correcting for the other covariates. However, these multiple timelines are learned independently and cannot be compared directly in order to exhibit the regions that are mostly affected by a covariate, as it may not be statistically significant. Resorting to statistical testing, using t-tests under the null hypothesis that the regression coefficient is 0, allows circumventing this issue as it normalizes all the effect sizes by the natural temporal scale associated with the region, and accounts for the uncertainties in estimation of the regression coefficients. In addition, for a fixed sample size, *p*-values are monotonically related to effect sizes, so lower corrected *p*-values can be interpreted as indicative of a bigger impact of the covariate, and allow comparison across brain regions and covariates.

We provide a map of cortical regions and a list of sub-cortical volumes that display significant differences when stratified for sex or APOE-*ε*4 status, regarding onset age and pace of atrophy, for AD, amyloid positive MCI and healthy aging. Corrected *p*-values higher than 0.05 are not displayed, and the remaining *p*-values are presented in logarithmic scale (base 10) for cortical thinning maps.

## 3. Results

**Figure 3:**
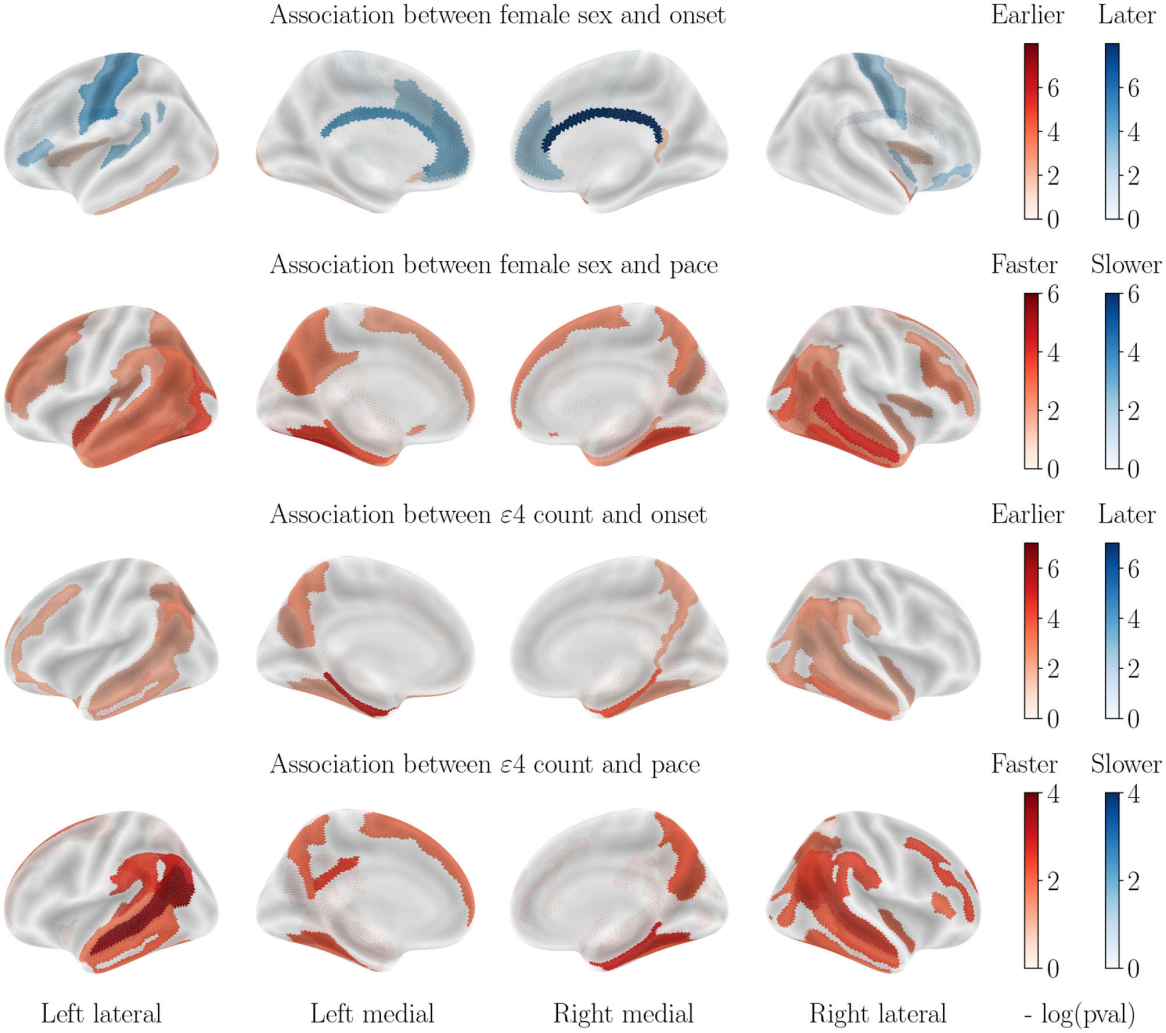
Cortical thinning over the course of AD progression. Legend bars show negative log *p*-values. It should be noted that blue values indicate that the considered covariate (female sex or APOE-*ε*4 genotype) is a protective factor, and red suggests a risk factor.

**Figure 4:**
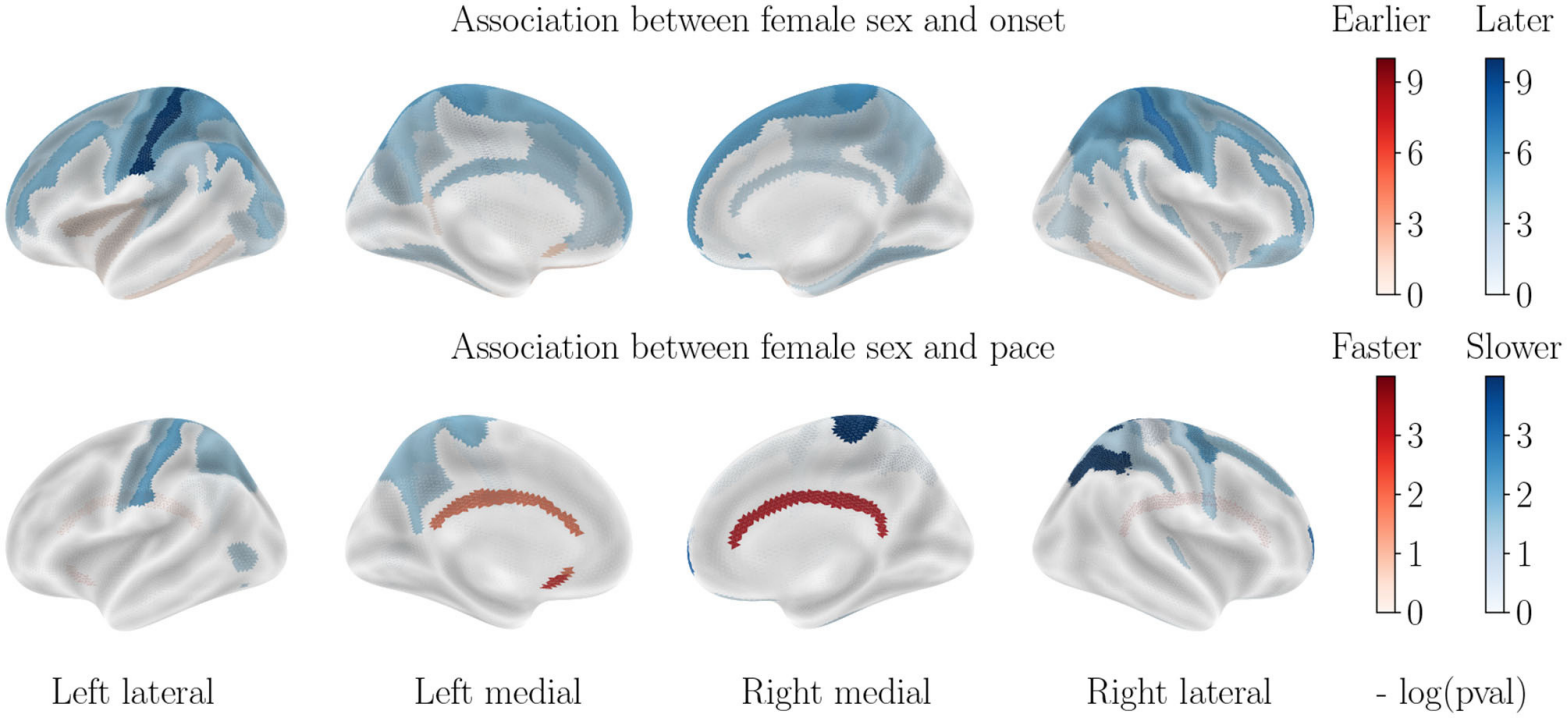
Cortical thinning over the course of cognitively normal aging. Associations with APOE-*ε*4 status are not displayed, as they are not significant.

**Figure 5:**
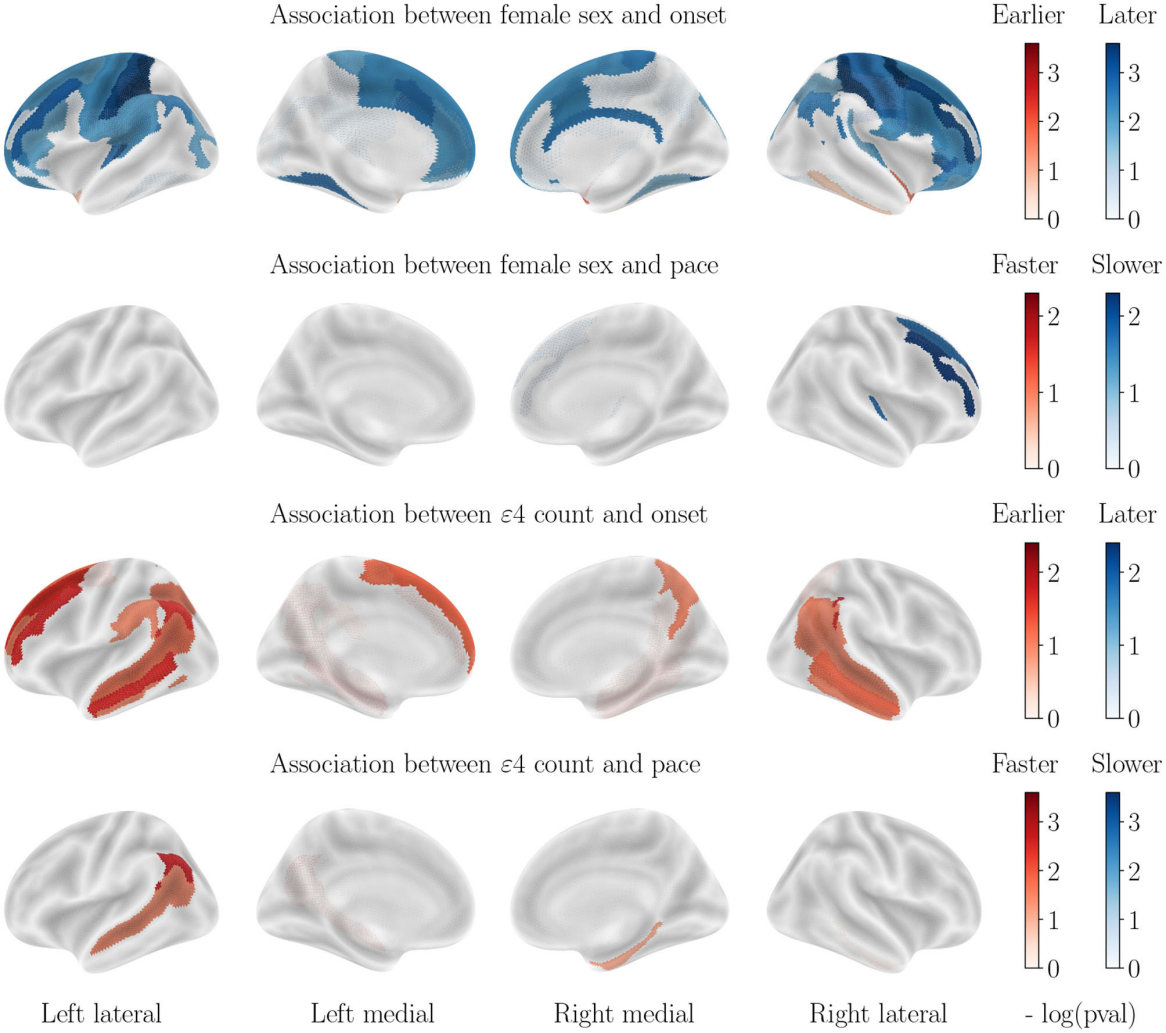
Cortical thinning over the course of MCI patients' aging.

Scales of log *p*-values vary between each plot and should be taken into account to evaluate the strength of the considered risk factor. For visualization purposes, cortical thinning maps are presented by cohort (AD in Figure 3, control cohort in Figure 4 and MCI in Figure 5) while all results for subcortical volumes are compiled in Table 2. Scatterplots of the raw data are provided in Supplementary material to illustrate the non-linear progression of repeated measurements over the course of disease progression, and the relevance of the chosen modeling framework. It should be noted that absolute pairwise-correlations between covariates are 0.02 for APOE/sex, 0.05 for APOE/education, and 0.18 for sex/education, which means that regressing with regard to the education level might mitigate the strength of the observed sexual dimorphism, but interactions between covariates are less likely for the other pairs.

**Table 2:** Significant correlations with subcortical atrophy.

		**Correlations with sex**			**Correlations with APOE-**ε4		
		*p*_*val*_ **CN**	*p*_*val*_ **MCI**	*p*_*val*_ **AD**	*p*_*val*_ **CN**	*p*_*val*_ **MCI**	*p*_*val*_ **AD**
Amygdala (L)	Onset	-	-	8.1e-03	-	4.9e-03	1.9e-07
	Pace	-	-	1.2e-06	-	2.2e-03	7.0e-06
Amygdala (R)	Onset	-	-	7.4e-05	-	3.2e-02	1.4e-06
	Pace	-	-	2.3e-05	-	2.5e-05	1.9e-02
Caudate (L)	Onset	5.5e-07	6.7e-04	1.7e-04	-	-	-
	Pace	-	-	-	-	-	-
Caudate (R)	Onset	1.2e-04	1.8e-04	8.3e-04	-	-	-
	Pace	-	-	-	-	-	-
Hippocampus (L)	Onset	4.7e-06	1.2e-02	-	-	3.0e-03	1.5e-07
	Pace	-	-	3.9e-05	-	5.7e-04	1.2e-02
Hippocampus (R)	Onset	1.1e-04	1.2e-02	-	-	2.1e-03	1.1e-09
	Pace	-	-	1.2e-04	-	3.9e-04	2.6e-02
Pallidum (L)	Onset	4.6e-04	6.9e-04	4.9e-02	-	-	-
	Pace	-	-	-	-	-	-
Pallidum (R)	Onset	6.5e-03	1.8e-04	3.1e-05	-	-	-
	Pace	-	-	-	-	-	-
Thalamus (L)	Onset	1.3e-06	7.8e-12	1.5e-07	-	-	-
	Pace	-	1.1e-02	-	-	-	-
Thalamus (R)	Onset	4.1e-06	4.6e-09	5.5e-08	-	-	-
	Pace	-	-	-	-	-	-

For all the significant features, APOE-*ε*4 genotype correlate with lower onset age and higher pace, and female sex correlates with higher onset age and higher pace, as illustrated by the colors (red for earlier or faster and blue for later or slower).

### 3.1. Impact of sex on cortical thinning for AD progression

Almost all regions of the cortex display a significantly higher progression pace for female patients, with the exception of the motor cortex, sensory areas and inferior frontal lobe. Regions that are most accelerated in females are the entire temporal lobe, the middle and superior frontal lobe, the entire occipital lobe and the anterior and medial parietal lobe. Onset ages are, on the other hand, more homogeneous across sexes. The motor cortex, the cingulate gyrus and the medial parietal lobe display an earlier onset for men, while the inferior temporal lobe and inferior frontal lobe display an earlier onset age for female patients. All other regions do not allow rejecting the null hypothesis.

### 3.2. Impact of APOE-*ε*4 genotype on cortical thinning for AD progression

Almost all regions of the cortex also display a significantly higher progression pace for APOE-*ε*4 carriers. The most affected regions are located roughly in the same areas as for the impact of sex, but with different regions of highest intensity, and an overall lower effect. The temporal lobe, the parietal lobe and the frontal lobe also display an earlier onset for APOE-*ε*4 carriers. The region that presents the most advanced onset for ε4 carriers is the hippocampal gyrus.

### 3.3. Impact of sex on cortical thinning for healthy aging

Contrary to what was seen in AD progression, the sexual dimorphism for healthy aging manifests mainly through a significantly earlier onset age for male subjects, especially in the parietal and frontal lobes. Only the inferior temporal lobe and subcallosal gyrus display a slightly earlier onset for women. On the other hand, progression paces are similar across sexes except for the cingulate gyrus that is much accelerated in female patients, while a few regions of the parietal lobe display a higher pace of atrophy for male patients.

### 3.4. Impact of APOE-*ε*4 genotype on cortical thinning for healthy aging

In order to assess the impact of the APOE-*ε*4 genotype on cognitively normal aging, we also stratified regarding this factor. Interestingly, the ε4 allele carriers do not display significantly different patterns of cortical thinning across healthy aging, which leads to believe that APOE-*ε*4 by itself does not cause the accelerated atrophy of the brain but only serves as one cog in the unraveling of AD. It should be noted however that the small amount of ε4 carriers in the healthy cohort can bias this result.

### 3.5. Influence of both covariates for patients with MCI

The progression paces show little significant correlation with both covariates, however, for onset age the correlations with sex are similar to those displayed for healthy aging while the correlations with APOE-*ε*4 status are similar to those displayed for AD progression, although of a weaker effect.

### 3.6. Correlations with the patterns of atrophy for subcortical structures

For healthy aging, male sex correlates with an earlier onset age for most regions with no significant differences in pace of atrophy, while no significant correlation is found for APOE-*ε*4 genotype. For the AD cohort on the other hand, female sex correlates with an earlier onset for most regions, and higher paces of the hippocampi and bilateral amygdalas while APOE-*ε*4 genotype correlates with earlier onset and higher pace for the hippocampi and bilateral amygdalas. We reach the same conclusion regarding the MCI cohort as for cortical thinning : sex correlates with atrophy similarly to the healthy cohort, while APOE-*ε*4 genotype correlates similarly to the AD cohort. Regions without significant correlations are not displayed.

### 3.7. Differences between left and right hemisphere

For AD progression, the left hemisphere displays more regions that differ significantly for both sex and APOE-*ε*4 stratification, but the associated t-values do not significantly differ between hemispheres. This confirms that atrophic patterns are asymmetric but not completely lateralized (83).

### 3.8. Impact of education level

**Figure 6:**
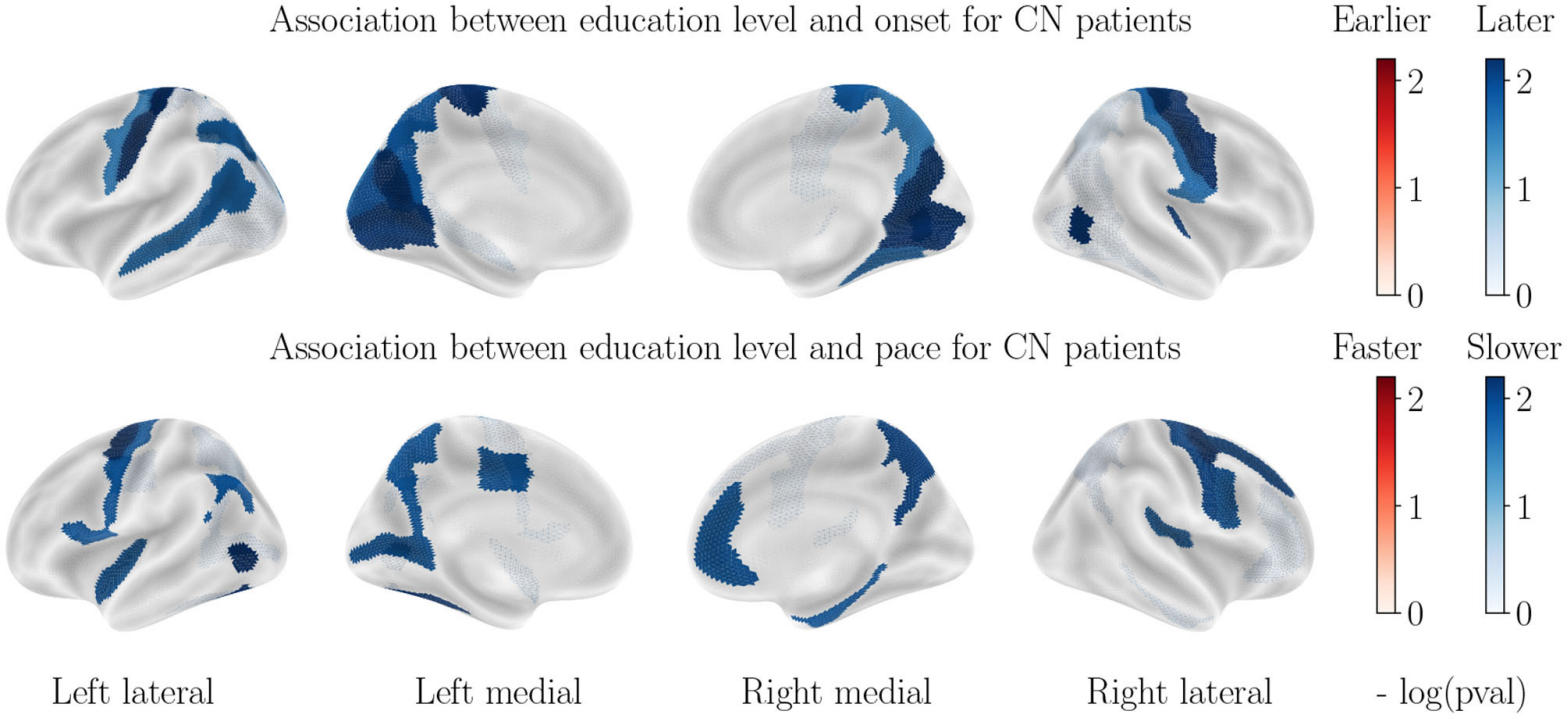
Correlation between education level and cortical thinning dynamics for CN patients. Correlations observed for the AD patients are not significant and thus not displayed.

Figure 6 illustrates the impact of education level on cortical thinning patterns, after accounting for sex and APOE−ε4 status. Correlations are only found in the healthy cohort, and higher education level correlates with higher onset age for the postcentral gyrus, the medial parietal and medial occipital lobes, as well as the left superior temporal lobe, and lower pace of atrophy for parts of the frontal and occipital lobes. No significant correlation is found for the AD cohort, in line with former studies (84). For subcortical volumes, only the hippocampal atrophy is delayed for more educated healthy patients, and no significant correlation is found for AD patients. One hypothesis is that education level is not, in itself, helping delay the atrophy for healthy controls, but acts as a proxy of lifestyle healthiness, which influences metabolic pathways and brain atrophy. In AD cohort, that small preserving effect is likely to be canceled by the influence of other covariates that cause massive atrophy.

### 3.9. Validation on gray matter density maps from ADNI

Despite the strong sexual dimorphism in regional gray/white matter ratios, we recover most results from the main analysis. For AD patients, the same regions display a strong dimorphism: almost all the temporal lobe, frontal lobe and occipital lobe display a much stronger atrophic pace for women, while the postcentral gyrus displays an earlier onset for men. APOE−ε4 allele also correlates with a faster pace and earlier onset in the medial temporal and parietal lobes, as well as in the temporal lobe and parts of the frontal lobe. For CN patients, we recover the absence of association with APOE genotype, while men display a much earlier onset for almost all regions (with emphasis on the parietal lobe and postcentral gyrus), while a few isolated regions of the frontal and temporal lobe display a faster pace of decline for men. The conclusions regarding the MCI cohort and the impact of education level are also confirmed. The differences with the main analysis are thus only a matter of a few isolated regions and of the order of magnitude of the statistical associations. We provide the association figures for cortical thinning and the tables for subcortical structures in Supplementary material.

## 4. Discussion

Our study allows isolating the contributions from sex, APOE status, and education level on both onset age and pace of atrophy for each region of the cortex and brain, while accounting for the other covariates. We find that:

- Sex is as important a risk factor as APOE-*ε*4 genotype for cortical thinning and brain atrophy for AD patients, as both stratification yield effect sizes that are not significantly different. This finding is important, given that most studies refer to APOE polymorphism as the biggest risk factor for AD, with little to no attention paid to the sexual dimorphism. Besides, focus is often put on specific regions such as the hippocampus and enthorinal cortex, as well as cognitive abilities, while the dimorphism in structural alteration is manifest for a wide variety of brain regions, that may also play an important role in the progression of the disease.
- Female AD patients decline much faster than male patients, however, the onset of the atrophy is still a little earlier for male patients, in line with the healthy aging patterns. This point is revealed by the use of longitudinal studies and has important implications for practitioners for the follow-up of patients. It suggests that the disease strikes female patients with more intensity, but not earlier. Besides, former studies found that female sex leads to both earlier and faster cognitive decline (84), which suggests different compensation mechanisms in males and females, that translate cortical thinning and brain atrophy into cognitive alterations.
- Higher education seems to be a slightly protective factor for healthy patients, but is not significant in AD progression. Given the strong protective effect of higher education on cognition (20), it is interesting to note that such protective effect does not happen at the structural level, but rather a functional level.
- For the amyloid positive MCI cohort, the influence of sex is similar to that of the healthy cohort, while the effect of APOE-*ε*4 genotype is similar to that of the AD cohort, although of an overall weaker effect. This is in line with the idea that the sexual dimorphism of AD manifests itself at the later stages of AD, rather than at the prodromal stage, with a harsher decline in structure and function, but not an earlier onset. It is also coherent with the absence of significant difference in prevalence before the age of 75.

It is important to note that our study focuses specifically on structural alterations in the brain, and that this is only one piece of the larger puzzle of AD pathology. Nevertheless, structural changes in the brain are a key feature of the disease, and understanding the dynamics of brain atrophy is an important step toward developing effective interventions. Besides, exhibiting a different influence of risk factors on neuronal death than what is expected from previous knowledge on studies of cognitive decline hints that said risk factors differentially influence each cog of AD pathogenesis.

Several limitations and design choices need to be acknowledged in order to put results in perspective.

### 4.1. Clinical characterization of diagnosis in ADNI

For the control cohort, we used the CN patients in ADNI, because it allows a fair comparison between AD progression and cognitively normal aging, without acquisition biases. Since ADNI is not an epidemiological study on healthy aging, the cognitively normal patients may not represent accurately the general population because of ADNI's inclusion criteria. For the MCI cohort, it is important to note that MCI does not always lead to AD as other factors can cause patients to have it. The prevalence of MCI symptoms are found to be between 15 and 20% for patients over 60 years old (85), and roughly 8 to 15% of these convert to AD each year (86). In order to make the cohort less heterogeneous, we decided to only keep amyloid positive patients in order to interpret this cohort as describing AD pathogenesis. Petersen et al. (87) assert that the recruited cohorts for CN, MCI and AD patients successfully describe the associated clinical status.

### 4.2. Validation cohort

Reproducing this study is complicated because of the lack of a publicly available longitudinal database of the same scale as ADNI, and the difficulty to fit longitudinal models to pooled databases with varying protocols. Our statistical pipeline requires splitting the patients between CN and AD patients, while only keeping patients with more than 2 visits with a t1-MRI scan. For instance with those criteria, in the Australian Imaging Biomarkers and Lifestyle Study of Aging (AIBL), often used to validate findings on ADNI, the AD cohort only contains 38 patients with a total 105 visits, and the healthy cohort 128 patients with 431 visits, before the additional stratification by sex or APOE-*ε*4 genotype. The sample sizes are thus too small to provide enough statistical power to reproduce the regional map of significant correlations.

### 4.3. Impact of the chosen atlas

It must be taken into account that the choice of a different atlas might yield different results. One anatomical region that is positively associated with a covariate can span many ROIs or on the other hand be split between ROIs and prevent the detection of the true underlying association. On the other hand, our mixed-effect formulation cannot be fitted directly to vertex-wise thickness measurements because the onset age (or horizontal intercept) will not converge for features that display little progression compared to the feature noise.

### 4.4. Regions normalization

Since subcortical regions' volumes are correlated with the volumes of the skull and brain, it is standard practice to normalize by the intracranial volume in order to allow a fair comparison between subjects. The impact of such normalization is discussed in former studies: in (88–90) authors suggest that subcortical volumes should be normalized with total intracranial volume, while cortical thicknesses should not, for diagnosis prediction and progression models. On the other hand, (91) find that cortical thicknesses should be normalized with either intracranial volume or average thickness to help predict the cognitive status of a patient. Luders et al. (73) discuss the effect of normalizing cortical thicknesses to allow a fair comparison between healthy male and female subjects. Allometric scaling is often used to circumvent this issue, but Williams et al. (92) find that allometric and linear scaling yield similar effect sizes and coefficients when evaluating the effect of sex and age on brain measurements.

### 4.5. Interpretability of the onset age

As opposed to the straightforward pace of atrophy, the onset age needs to be interpreted with caution. It represents the age at which a patient crosses a chosen threshold (chosen to be the average value for the whole cohort). Brain atrophy does not have a well-defined starting point, and the notion of onset age is thus to be understood with regard to a reference point in the average progression. For instance, the earlier onset ages for men in the CN cohort can be understood as describing the fact that cortical thicknesses are significantly thinner than for women. Since the difference in onset gets way smaller for the AD cohort, it agrees with former findings that the gap between men and women closes as neurodegeneration progresses, with a greater pace of decline for women in the late stages of the disease (56, 57).

### 4.6. Clinical implications

The discrepancies in alteration patterns of the brain between sexes and APOE genotypes reinforce the idea that the disease manifests differently between subpopulation and care should be provided accordingly. Besides, clinical trials that monitor the effect of a drug on neurodegeneration should put an emphasis on both having more representative demographics after enrollment, and evaluating the impact of drugs in a stratified manner as patients are likely to react differently to the drug depending on both the explored genetic risk factors. It is also interesting to note that most regions significantly affected by the considered risk factors are known to be associated with AD (e.g., medial temporal lobe, limbic system, frontal lobe, etc.). This work could be interpreted jointly with studies that describe the sexual differences in associations between cortical thickness and cognition such as (57) in order to provide a complete description of the sexual dimorphism of neurodegeneration and its link with AD cognitive symptoms.

### 4.7. Perspectives

This statistical pipeline can be applied to other modalities of neuroimaging data. For instance, (93) applies the same statistical pipeline to FDG-PET scans instead of structural MRI in order to assess the sexual dimorphism of AD and influence of APOE genotype regarding brain hypometabolism, which complements the study of brain atrophy. Other imaging modalities specific to AD, such as amyloid-PET or tau-PET, are also under investigation within this pipeline. A strong sexual dimorphism for the spreading patterns of pathological protein tangles in the brain would prompt the latest clinical trials, that choose amyloid load as a primary disease outcome, to put great care in evaluating the differentiated results between male and female patients.

## 5. Conclusion

Structural alterations of the brain happen before the onset of cognitive decline, and the collection of MRI-derived features reveals varying patterns of atrophy between subgroups of patients. Female sex, APOE-*ε*4 genotype and low education level have been identified as the biggest risk factors for cognitive decline, but their impact on early-stage structural alterations is not yet well understood. In this work, we described the disentangled effects of these 3 covariates on the onset and pace of regional atrophy and cortical thinning. This reveals that female sex is actually as strong a risk factor for neurodegeneration as APOE-*ε*4 genotype (Figure 3), and women experience much faster atrophic rates after correcting for the APOE genotype. APOE-*ε*4 genotype leads to earlier and faster atrophy for most regions of the brain for AD patients, but not for healthy patients. Healthy patients with higher education experience slightly delayed atrophy, but not patients with neurodegeneration. The results were validated with congruent findings from the same study applied to the regional loss of gray matter density. This work calls for further exploration of the sexual dimorphism of AD regarding structural alterations.

## Data availability statement

The original contributions presented in the study are included in the article/Supplementary material, further inquiries can be directed to the corresponding author.

## Author contributions

BS and SD jointly conceived and designed the analysis. BS implemented the research, performed the analysis, and wrote the manuscript. SD contributed to methodological validation of the analysis and writing and editing of the manuscript. Both authors contributed to the article and approved the submitted version.
