# Supplementary material for: Impact of sex and APOE-ε4 genotype on patterns of regional brain atrophy in Alzheimer's disease and healthy aging
Source: Front Neurol. 2023 Jun 2;14:1161527. doi: 10.3389/fneur.2023.1161527 (PMC10272760; doi:10.3389/fneur.2023.1161527)
Supplement: Supplementary file 1 [file Data_Sheet_1.PDF]

# Impact of sex and APOE-$\varepsilon$4 genotype on patterns of regional brain atrophy in Alzheimer's Disease and healthy aging. Supplementary materials

Benoît Sauty[a,*], Stanley Durrleman[a], for the Alzheimer's Disease Neuroimaging Initiative

[a]*Sorbonne Université, Institut du Cerveau - Paris Brain Institute - ICM, CNRS, Inria, Inserm, AP-HP, Hôpital de la Pitié Salpêtrière, Paris, France*

## 1. Reconstruction error and comparison with acquisition noise

In Fig. 1, the distributions of fit error or the three cohorts (CN, MCI and AD) are compared to the acquisition and processing noise of subcortical volumes. In Fig. [2,3,4], the same errors are displayed for cortical thicknesses.

The overlapping of the distributions hints that the models could not improve the reconstruction error without overfitting. For all features, reconstruction errors gets slightly larger as the selected cohort gets more cognitively altered. That could be attributed to the considered brain regions being smaller due to a higher level of neurodegeneration, and thus yield a higher error in parcellation. That could also explain why for some features the reconstruction errors of the CN cohort are marginally lower than the re-test errors that are computed with patients at all stages of brain atrophy.

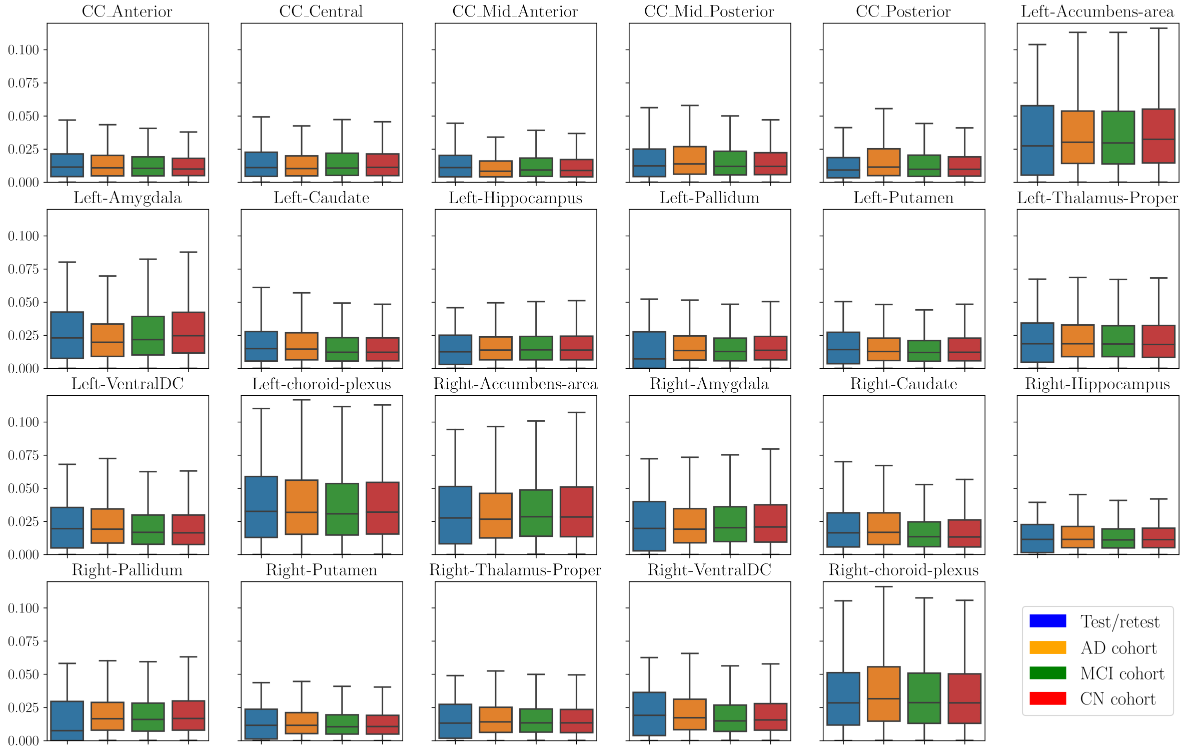


Figure 1: Comparison of measurement noise (blue) and fit errors (AD: orange, MCI: green, CN: red) for subcortical volumes.

*Corresponding author: Institut du cerveau, 47 bd de l'Hôpital, 75013 Paris, France, Tel.: +33631040386; e-mail: benoit.sauty-de-chalon@inria.fr

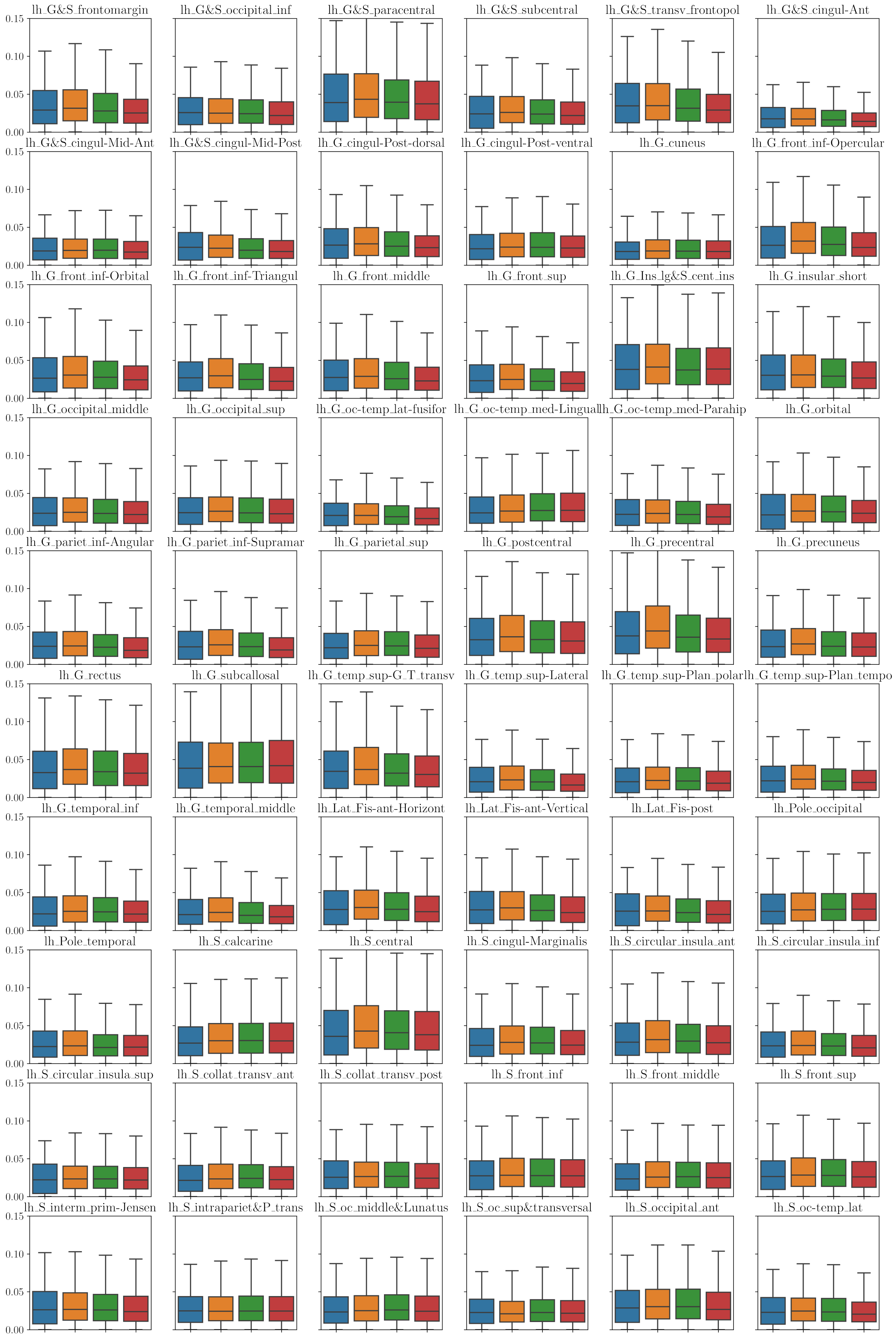


Figure 2: Comparison of test/re-test measurement noise (blue) and fit errors (AD: orange, MCI: green, CN: red) for cortical hicknesses. Part 1/3.

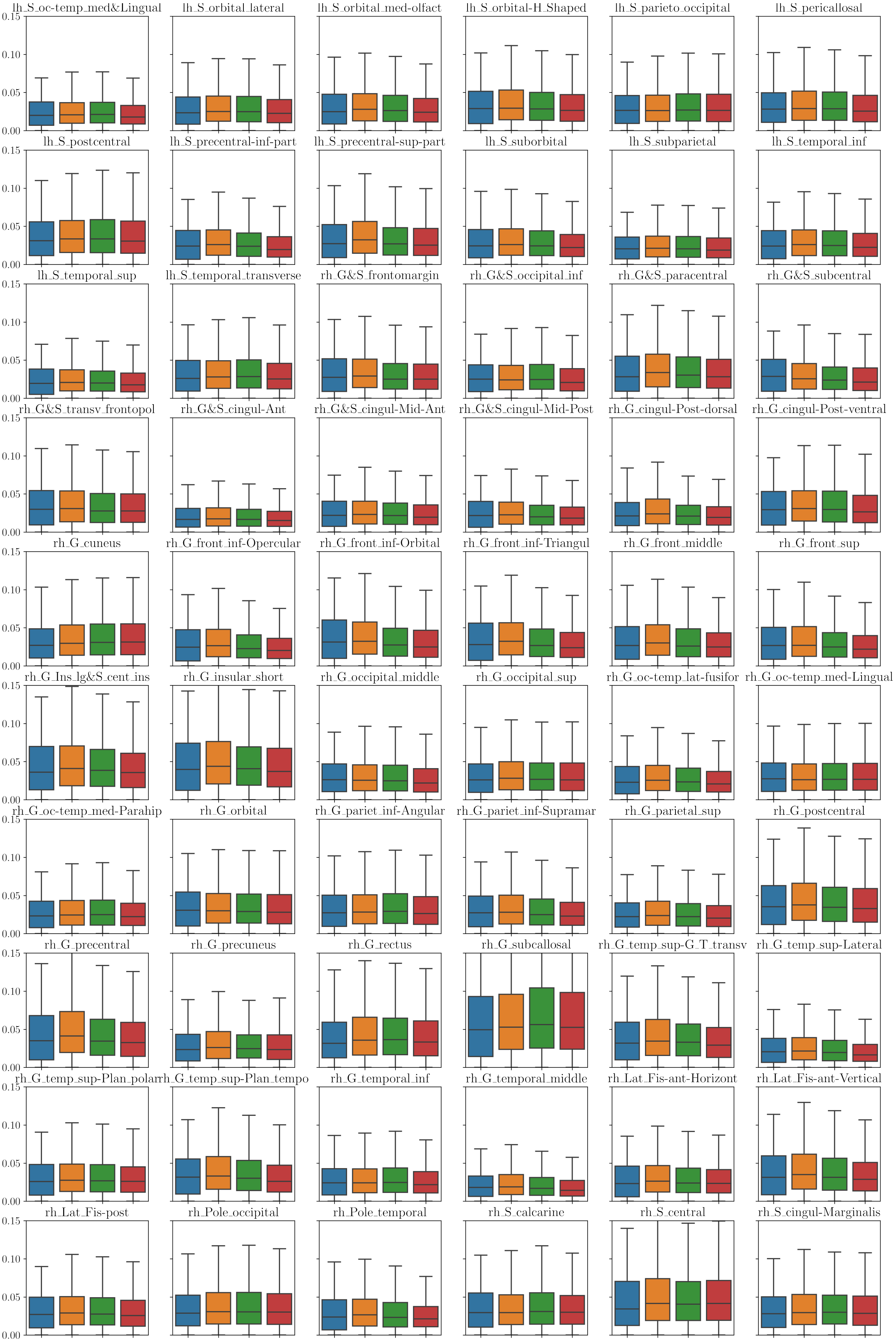


Figure 3: Comparison of test/re-test measurement noise (blue) and fit errors (AD: orange, MCI: green, CN: red) for cortical hicknesses. Part 2/3.

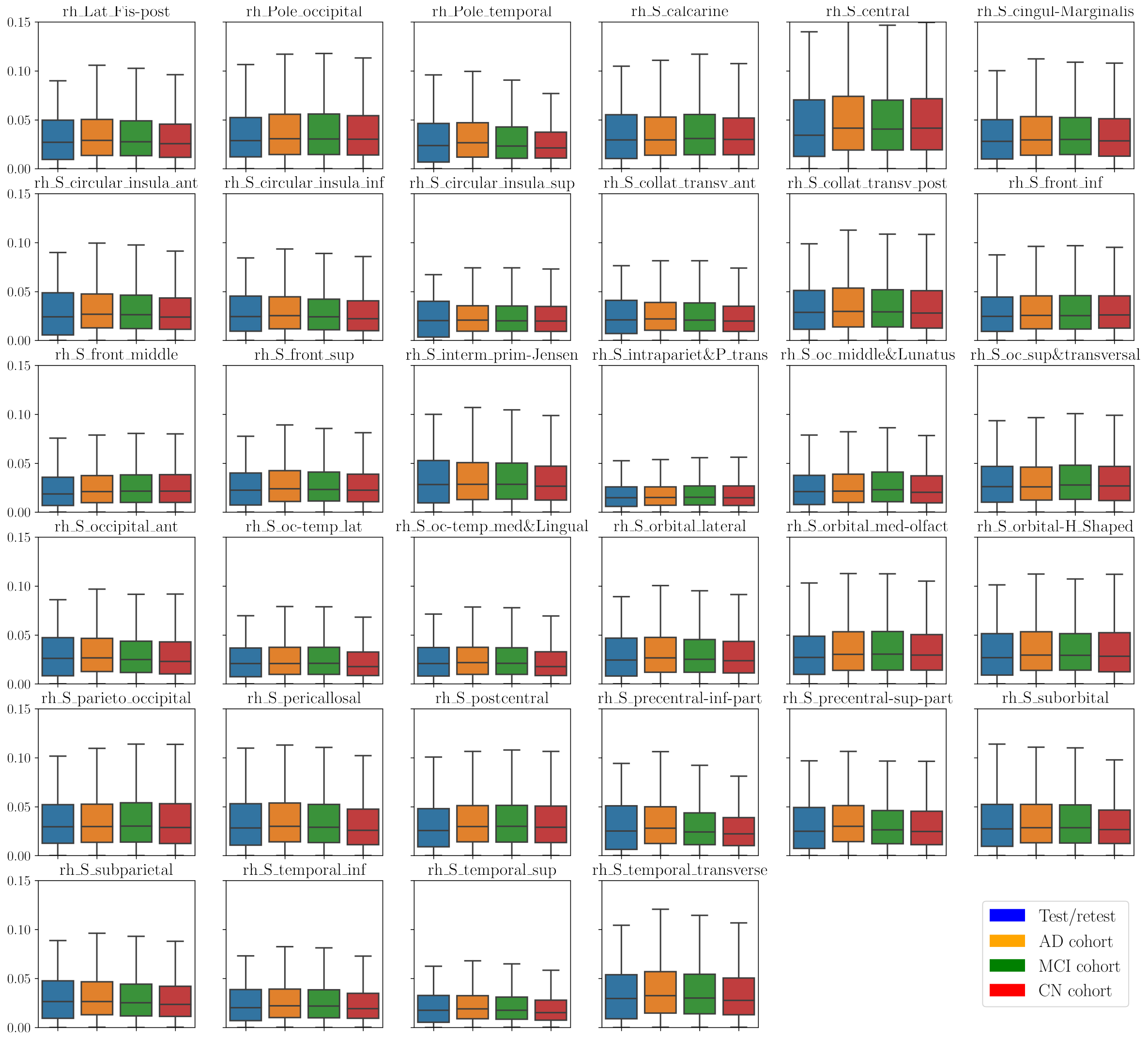


Figure 4: Comparison of test/re-test measurement noise (blue) and fit errors (AD: orange, MCI: green, CN: red) for cortical hicknesses. Part 3/3.

## 2. Longitudinal scatterplots

In order to illustrate the relevance of the chosen non-linear mixed-effect model with a temporal reparametrization, we display the scatterplots of repeated measurements (4.4 visits per patient on average), both with regard to clinical patients' age, and to the learned pathological age. Due to the large amount of cortical regions for each cohort, we chose to only display a fraction of the features for the AD cohort, in order to improve readability.

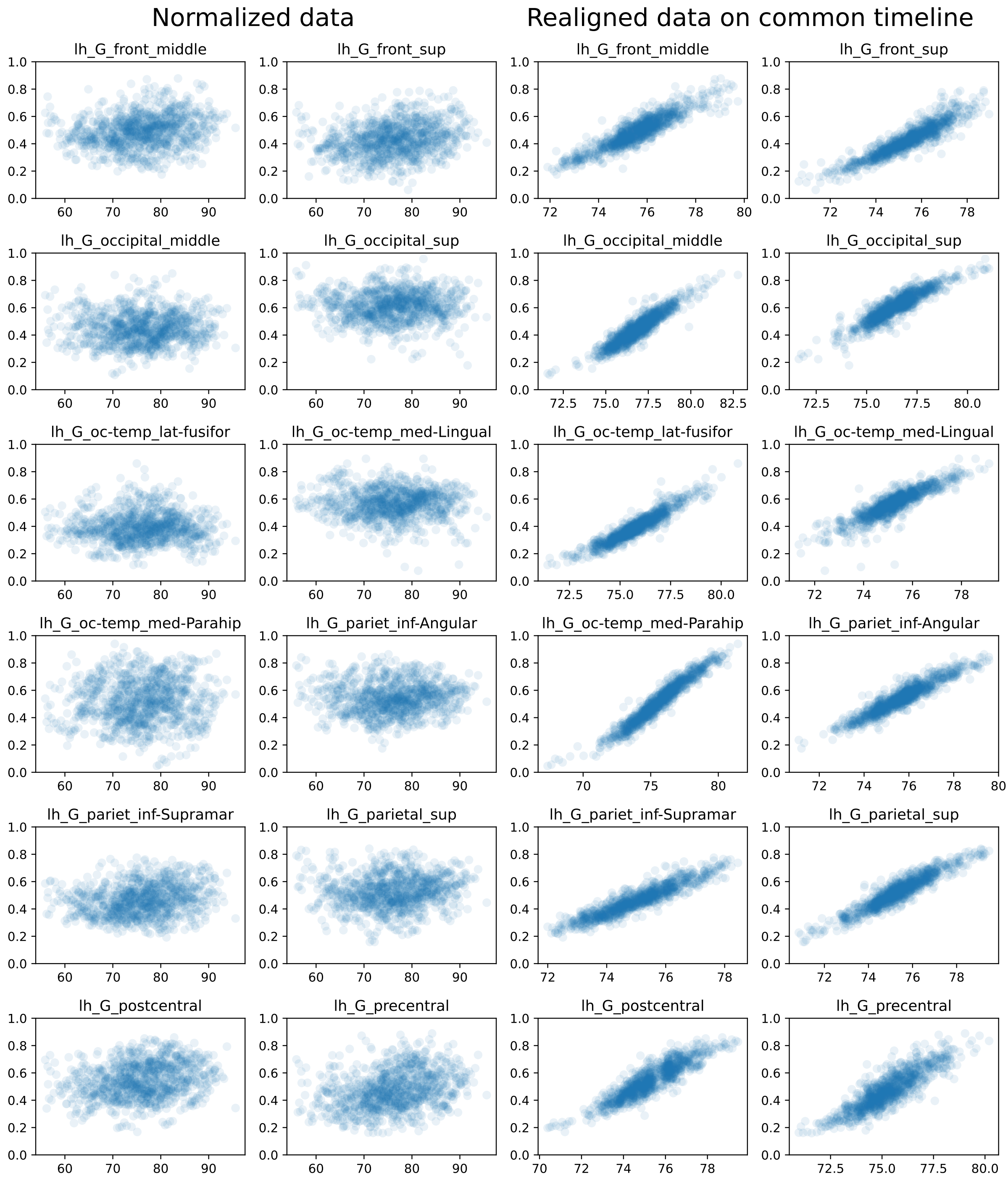


Figure 5: Scatterplots of cortical thickness measurements for 12 randomly selected regions of the Destrieux atlas for the AD cohort. The first two columns represent real measurements over time, while the last two columns represent the same measurements over the learned pathological timeline, using the onset age and pace of decline for the affine time reparametrization. It should be noted that, although the raw data share the same time of visits (between 60 and 90), the learned pathological progression is different for each feature.

Spaghetti plots that correspond to the same data are also provided to illustrate the longitudinal aspect of the data. Each color represents one patient. The noisiness of the data is visible.

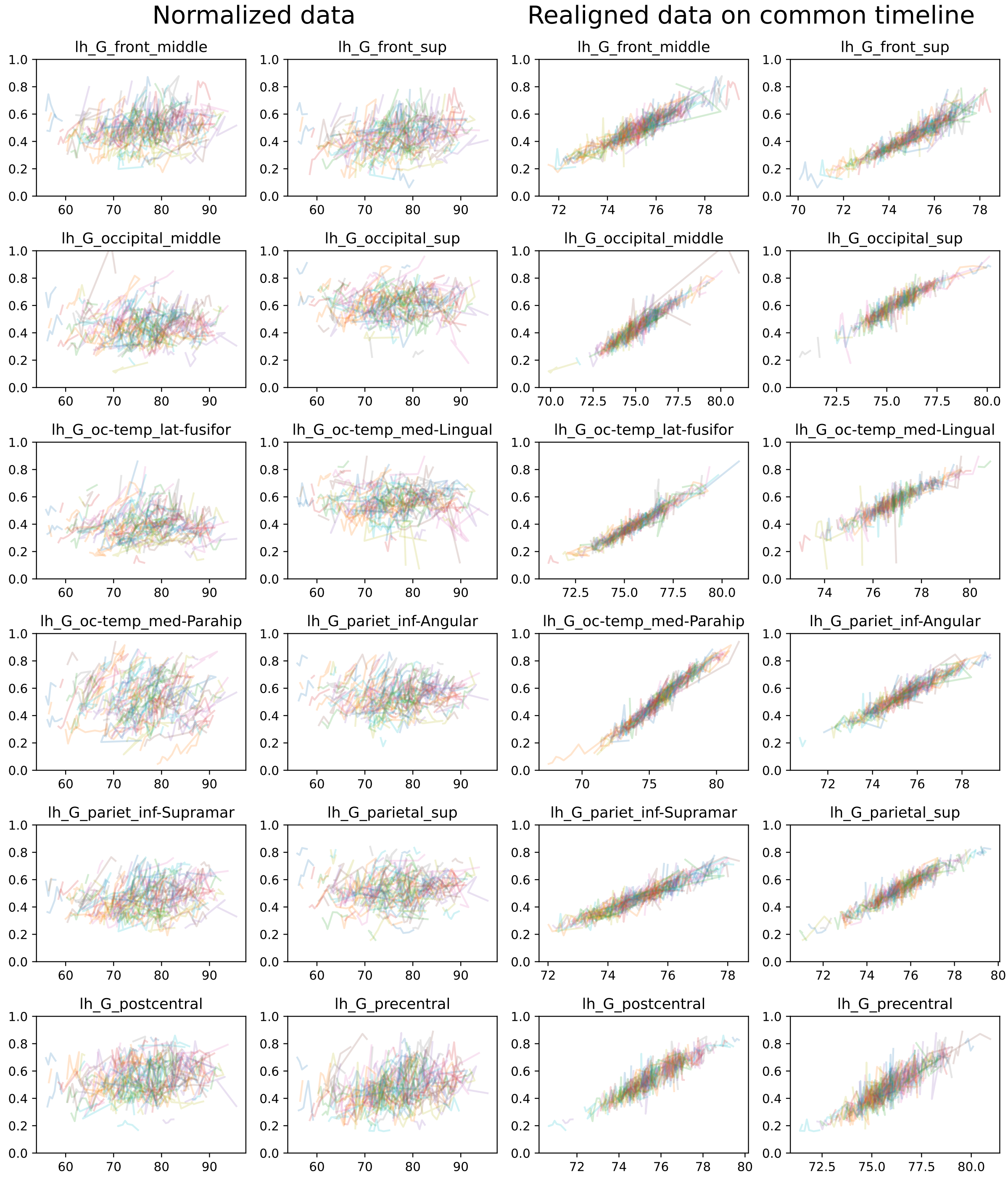


Figure 6: Spaghetti plots of longitudinal cortical thickness measurements for the same regions of the Destrieux atlas for the AD cohort. The first two columns represent real measurements over time, while the last two columns represent the same measurements over the learned pathological timeline, using the onset age and pace of decline for the affine time reparametrization.

## 3. Bias correction for MRI field strength

As mentioned in the main article, cortical thickness measurements from FreeSurfer are known to be biased with regard to the field strength of the MRI scanner. As is common in longitudinal designs, in which data from 1.5 and 3T scanners are pooled, we remove the additive bias by matching the means of the distributions. The cortical thickness measurements presented below are normalized and flipped around .5 in order to be increasing over time. This means that 3T scans yield higher thicknesses on average for all the considered features, which is consistent with the literature.

Before homogenization, patients with visits that use different field strengths display significantly higher reconstruction errors from the longitudinal model, for most regions, while the same models after homogenization yield unbiased errors with regard to field strength. This hints that the homogenization removes the intra-patient's discontinuities that arise from varying field strengths.

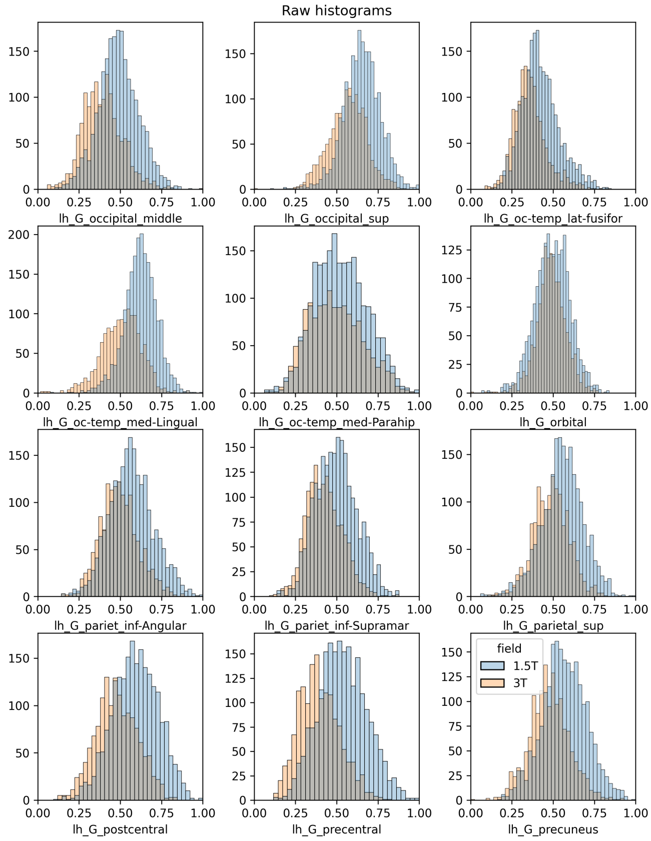


Figure 7: Histogram of cortical thickness measurements for 12 randomly selected regions of the Destrieux atlas for the AD cohort. Distributions are stratified by the field strength of the MRI.

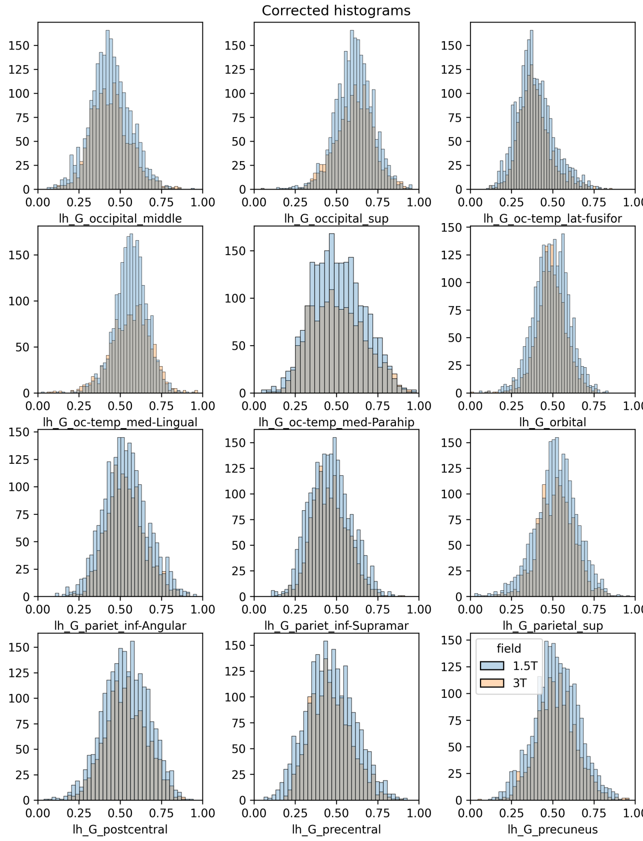


Figure 8: Histogram of *corrected* cortical thickness measurements for the same regions. Additive bias is removed and histograms align across field strengths.

## 4. Patients selection

For the sake of reproducibility, we display here the unique ADNI ID of the selected patients, with the ages that correspond to all the visits that we considered. This csv dump can be parsed to recover the exact same visits. It consists of a list of tuples (ID,TIME) separated by spaces.

For the AD cohort:
ID,TIME 619,77.5 619,78.0 619,78.5 619,79.6 729,65.1 729,65.7 729,66.2 729,68.1 729,66.7 729,67.2 729,69.1 729,72.1 729,71.1 729,70.1 816,71.3 816,70.8 816,72.2 938,84.2 938,82.2 938,82.7 938,83.2 954,69.3 954,69.8 954,70.9 954,70.2 955,78.2 955,78.7 1018,70.7 1018,71.2 1018,71.7 1018,72.7 4225,71.9 4225,70.9 4225,70.4 4225,69.9 4225,77.0 5018,74.3 5018,73.8 5018,73.3 1057,61.2 1057,61.9 1057,62.2 1057,63.3 1057,64.2 1057,65.3 1057,66.3 1059,84.5 1059,86.5 1059,85.0 1074,85.0 1074,86.0 1074,87.1 1074,86.5 1074,88.2 1074,89.3 1074,93.2 1074,91.5 1074,95.6 1257,84.9 1257,86.0 4136,67.7 4136,67.2 4136,66.7 4373,71.6 4373,71.1 4892,76.1 4892,75.7 4892,75.1 221,67.4 221,69.4 221,67.9 221,68.4 222,86.4 222,87.4 222,85.9 222,86.9 222,87.9 223,78.0 223,78.5 223,79.0 223,80.0 223,81.0 572,78.7 572,79.2 572,79.7 572,80.2 572,80.7 572,81.7 814,71.5 814,72.0 814,73.0 814,71.0 929,82.0 929,82.5 1341,73.6 1341,71.6 1341,72.1 1341,72.6 4707,69.0 4707,68.5 4707,68.0 4910,82.4 4910,81.9 4910,81.4 5038,82.5 5038,81.9 5038,81.5 5119,78.0 5119,77.0 547,75.6 547,76.1 547,76.6 547,77.6 675,78.8 675,79.3 675,79.9 675,80.9 1130,71.1 1130,71.6 1130,72.1 1130,72.6 1130,74.1 1130,75.1 1130,73.2 1130,76.2 4153,81.3 4153,80.3 4153,79.8 4153,79.3 4192,84.2 4192,83.2 4192,82.8 4192,82.2 4546,71.5 4546,71.0 4867,75.5 4867,74.9 41,71.4 41,70.9 41,71.9 41,72.4 41,72.9 41,73.9 41,75.1 101,73.6 101,74.1 101,74.7 101,75.1 101,75.5 101,76.6 101,77.7 101,80.8 101,79.7 128,64.1 128,64.6 128,65.1 128,65.6 128,66.1 128,67.1 128,68.1 249,72.0 249,72.5 249,76.1 249,73.1 249,73.6 249,74.0 249,75.0 293,88.8 293,87.7 293,88.3 293,89.2 293,89.7 293,90.7 316,81.4 316,80.9 316,81.9 316,83.0 344,78.8 344,79.8 344,80.3 344,80.8 344,81.9 344,83.0 1304,75.2 1304,74.7 1339,80.0 1339,79.5 1339,80.6 1339,81.6 4568,72.7 4568,71.8 4568,71.2 4568,70.7 4911,75.6 4911,75.1 4911,74.5 1030,67.4 1030,67.9 1030,68.4 1030,68.9 1030,71.5 1030,70.4 1030,75.4 1030,74.4 1030,73.4 1030,72.4 1334,64.2 1334,64.7 1354,58.9 1354,59.4 4324,67.0 4324,64.9 4324,63.8 4324,63.3 4324,62.8 4324,69.5 4324,70.4 5027,76.5 5027,75.9 5027,75.5 5037,68.3 5037,67.9 5037,67.3 5252,57.0 5252,56.5 786,74.5 786,75.0 786,75.5 786,76.5 829,64.9 829,65.4 829,65.9 829,66.9 904,84.1 904,84.6 904,85.1 904,85.6 904,86.2 3,81.3 3,81.8 3,82.3 3,83.3 10,73.9 10,74.4 10,74.9 10,75.9 53,80.1 53,80.6 53,81.1 53,82.1 183,72.4 183,72.9 183,73.4 183,74.4 241,84.8 241,81.8 241,82.3 241,82.8 241,83.3 241,83.8 241,85.8 326,76.9 326,77.4 326,77.9 326,78.4 326,78.9 326,79.9 856,60.3 856,60.8 856,61.3 856,61.8 856,62.3 861,87.1 861,87.6 861,88.1 861,88.6 861,89.1 861,90.1 1080,81.4 1080,81.9 1080,82.4 1080,82.9 1080,83.4 1080,84.4 1080,85.4 1282,77.0 1282,77.5 1282,78.0 1282,78.6 1282,79.0 1282,80.0 1282,81.0 2274,64.0 2274,64.5 2274,68.0 2274,67.0 2274,66.0 2274,65.0 4547,81.3 4547,79.3 4547,78.3 4547,77.8 4547,77.3 4547,82.7 4827,72.0 4827,71.5 4827,71.0 4827,76.1 4845,69.0 4845,68.5 4845,68.0 4893,72.1 4893,70.0 4893,69.1 4893,68.6 4893,68.1 4893,73.2 4912,70.2 4912,69.7 4912,69.2 4949,79.1 4949,78.6 4949,78.1 6303,70.4 6303,71.4 689,63.6 689,64.1 689,65.6 689,64.6 712,76.6 712,77.1 712,77.6 720,77.4 720,77.9 720,78.4 803,84.7 803,85.2 803,85.7 803,86.7 1033,73.3 1033,73.8 1033,74.3 1292,76.2 1292,76.7 1292,77.2 1292,77.7 4094,60.0 4094,60.5 4094,61.0 4094,62.0 4094,64.1 4094,66.1 4094,67.3 4094,68.4 4188,81.2 4188,79.2 4188,78.2 4188,77.8 4188,77.2 4188,83.1 5121,76.8 5121,78.8 6073,64.0 6073,62.9 6073,64.9 240,88.3 240,88.9 240,89.3 240,89.9 240,90.3 325,70.6 325,71.1 325,71.6 325,72.1 325,72.6 860,85.2 860,85.7 860,86.2 996,90.9 996,91.4 996,92.9 996,91.9 1161,79.8 1161,80.3 1205,83.0 1205,84.0 1205,83.5 5071,77.0 5071,76.5 5071,76.0 328,77.1 328,77.6 328,76.6 328,78.6 356,80.2 356,80.7 563,81.6 563,82.1 563,82.6 563,83.6 563,80.6 563,81.1 563,84.8 563,85.6 658,72.8 658,73.1 658,73.8 658,74.1 658,75.1 658,76.1 658,72.1 658,78.2 658,77.1 1095,80.3 1095,80.8 1095,81.4 4039,57.9 4039,56.9 4039,56.4 4039,55.9 4615,87.5 4615,87.0 4615,86.5 702,85.0 702,85.5 702,86.2 702,86.5 702,87.0 702,89.0 702,88.0 702,91.6 702,90.0 991,86.3 991,86.7 991,87.4 991,88.7 1117,68.9 1117,69.7 1117,69.9 1117,70.4 1117,70.9 1117,72.4 1117,74.4 1117,73.3 1121,56.2 1121,56.7 1121,57.2 1121,57.7 1121,58.2 1121,59.2 1326,66.8 1326,67.3 1326,68.3 1326,66.3 1326,67.8 1326,69.4 1326,70.4 1326,72.3 1326,71.5 4009,92.3 4009,91.3 4009,90.8 4009,90.3 4591,67.9 4591,66.9 4591,66.4 4591,65.9 4887,74.9 4887,74.4 4887,73.8 4902,78.6 4902,77.3 4902,76.3 4902,75.8 4902,75.3 4902,81.1 4902,82.4 5032,78.5 5032,78.1 5032,77.5 5057,76.3 5057,75.2 5057,81.5 57,77.3 57,77.9 57,78.3 57,78.9 57,79.4 155,81.6 155,82.1 155,82.6 155,83.1 155,81.1 155,84.1 286,65.9 286,66.4 286,66.9 286,67.9 335,83.9 335,84.4 335,85.5 335,83.4 406,77.8 406,78.2 406,78.8 406,79.8 406,79.4 406,80.8 450,69.9 450,68.4 450,68.9 450,69.4 450,70.4 450,71.4 633,83.3 633,83.8 633,84.3 633,85.3 682,68.0 682,68.5 682,69.0 682,70.0 2133,62.5 2133,63.0 2133,66.5 2133,65.5 2133,64.5 2133,63.6 2133,69.6 2133,70.7 4696,75.0 4696,73.5 4696,73.0 4733,75.8 4733,75.3 4252,88.5 4252,87.5 4252,87.0 4252,86.5 4293,69.7 4293,70.2 4293,70.7 4293,71.7 4293,73.7 4293,75.3 4293,76.3 4477,84.1 4477,83.1 4477,82.6 4477,82.1 4549,79.8 4549,79.5 4549,79.0 5012,77.4 5012,76.8 5012,76.3 5019,63.9 5019,63.4 5019,62.9 213,62.9 213,63.5 213,63.9 141,81.1 141,81.6 141,82.6 141,83.1 141,84.1 141,85.1 141,82.1 332,69.9 332,70.4 332,70.9 343,71.8 343,72.3 343,72.8 343,73.8 626,83.7 626,84.2 626,84.7 626,85.3 626,86.2 626,87.2 626,83.2 626,91.2 626,90.2 626,89.2 626,88.2 626,93.8 642,84.5 642,85.0 642,85.5 753,65.5 753,66.5 753,67.6 753,66.0 984,76.6 984,77.1 984,77.6 984,78.6 984,79.6 984,80.6 984,81.7 984,82.6 984,83.6 1109,77.8 1109,78.3 1109,78.8 1109,79.3 4659,89.9 4659,87.9 4659,86.9 4659,86.4 4659,85.9 4659,91.0 4718,79.5 4718,79.0 4718,78.5 7,75.4 7,75.9 7,77.4 129,80.2 129,80.7 129,81.2 129,82.2 219,74.9 219,74.4 543,72.2 543,73.5 543,74.2 750,75.6 750,76.1 750,76.5 750,77.1 750,77.6 1097,73.0 1097,74.0 1097,74.5 1097,75.0 1097,76.0 1097,77.0 1097,78.1 1351,71.8 1351,72.3 1351,72.8 1351,73.3 1351,73.8 1351,75.9 1351,74.8 1351,76.9 1394,77.1 1394,77.6 1394,78.1 1394,78.7 1394,79.1

1394,80.1 1394,81.1 6013,60.4 6013,61.4 6013,62.4 30,80.0 30,80.5 30,81.0 30,81.5 30,82.1 30,83.1 42,72.8 42,73.3 42,73.8 42,74.3 42,74.9 42,75.9 42,77.2 42,80.8 42,80.0 42,78.9 42,78.2 78,77.5 78,76.0 78,76.5 78,77.0 83,73.2 83,73.7 83,74.2 83,75.2 84,75.3 84,75.8 84,76.3 84,77.3 93,77.2 93,78.2 93,79.2 126,77.5 126,78.0 126,78.5 126,79.0 126,79.5 126,80.5 126,81.5 126,82.5 126,84.6 126,83.5 139,65.9 139,66.4 139,68.0 139,66.9 217,84.6 217,87.6 217,88.6 217,83.6 217,84.1 217,85.0 217,85.6 217,86.6 217,89.8 331,64.6 331,65.1 331,65.6 331,66.1 331,66.6 331,67.6 331,68.7 331,72.6 331,71.6 331,70.8 331,69.7 376,70.5 376,71.4 376,72.0 376,72.5 376,73.5 376,74.6 376,71.0 376,75.5 388,71.2 388,71.7 388,72.2 388,72.7 388,73.2 388,74.2 388,75.1 604,86.5 604,87.0 604,87.5 604,88.0 604,88.6 604,89.6 625,75.9 625,76.5 625,76.9 625,77.4 625,78.0 625,79.2 625,80.1 855,75.6 855,76.1 855,76.5 855,77.1 887,73.7 887,74.2 887,74.7 887,75.2 887,75.7 887,76.7 887,77.7 887,80.7 887,79.8 887,78.7 916,79.7 916,80.3 916,80.7 916,81.7 1126,80.4 1126,80.9 1126,81.4 1126,81.9 1126,82.5 1190,76.5 1190,77.0 1190,77.5 1190,78.5 1190,79.6 1190,80.7 1190,83.6 1190,85.7 1190,87.3 1190,88.6 1190,89.3 1247,72.5 1247,73.0 1247,73.5 1247,74.0 1247,74.6 1262,72.7 1262,73.2 1262,73.8 1262,74.7 1289,77.4 1289,77.9 4115,75.7 4115,67.4 4115,67.9 4115,68.4 4115,69.4 4115,71.8 4115,73.7 4115,74.7 4501,81.0 4501,80.0 4501,79.5 4501,79.0 6661,75.0 6661,76.0 1171,71.5 1171,72.0 1171,72.5 1171,73.5 1307,75.3 1307,75.8 1307,77.3 1307,76.3 1393,83.5 1393,82.5 1393,83.0 1393,84.0 1393,85.5 4223,76.8 4223,76.3 4223,75.8 4280,80.7 4280,80.2 4280,79.7 4905,73.7 4905,73.2 4905,72.7 5054,75.0 5054,74.5 5054,74.0 256,70.1 256,70.6 256,71.6 256,72.1 256,73.1 256,74.1 256,75.1 256,71.1 256,76.1 404,87.8 404,88.3 404,88.8 404,89.8 408,82.2 408,82.7 408,83.2 408,83.7 408,84.2 408,85.2 408,86.2 408,87.2 408,89.2 408,88.1 461,80.3 461,80.8 461,81.3 461,81.8 835,72.7 835,73.2 835,73.7 835,74.2 835,74.7 835,75.7 835,76.7 835,77.7 835,78.7 850,78.1 850,78.6 850,79.1 850,80.2 1081,85.0 1081,85.5 1081,86.5 1081,84.5 1082,69.5 1082,70.0 1082,70.5 1082,71.5 1254,84.3 1254,84.8 1254,85.3 1254,86.3 1385,69.5 1385,70.0 1385,70.5 1385,71.5 1387,85.6 1387,86.1 1387,86.6 1387,87.1 1387,87.6 1387,89.6 1387,90.6 4801,78.7 4801,78.2 4801,77.7 4802,84.2 4802,83.7 4802,83.2 4938,71.9 4938,71.4 4938,70.9 4962,81.1 4962,80.6 4962,80.1 4964,81.9 4964,81.4 4964,80.9 836,83.1 836,83.6 836,84.1 878,72.8 878,73.3 878,73.8 878,74.3 878,76.4 878,72.3 878,75.4 999,70.8 999,71.5 999,72.0 1056,71.1 1056,73.1 1056,71.6 1056,72.1 4307,79.5 4307,79.0 4307,78.5 294,79.2 294,79.7 294,80.2 294,80.7 294,81.2 294,82.2 321,68.3 321,68.8 321,69.3 554,71.6 554,73.6 554,72.1 554,72.6 568,79.8 568,80.3 568,80.8 568,81.3 568,81.8 830,70.8 830,71.3 830,71.8 830,72.3 830,72.8 830,73.8 830,75.8 1066,72.9 1066,73.9 1066,72.4 1066,73.4 1066,74.4 1066,75.4 1066,76.4 1066,78.5 1209,72.5 1209,73.0 1209,74.0 1209,72.0 4024,56.9 4024,56.4 4024,55.9 4149,75.3 4149,74.3 4149,73.8 4149,73.3 4149,80.5 147,59.7 147,60.2 147,60.7 147,61.8 187,77.1 187,77.6 187,78.1 187,78.6 187,79.1 214,65.1 214,65.6 214,66.1 214,66.6 214,67.1 214,68.1 214,69.4 214,70.2 214,73.1 214,72.2 214,71.2 400,69.2 400,69.7 400,70.2 400,71.2 978,73.1 978,73.6 978,74.2 978,74.6 978,75.2 978,76.1 1037,73.7 1037,74.2 1101,70.9 1101,71.4 1101,71.9 4755,71.7 4755,72.3 4755,72.7 6600,71.2 6600,72.2 511,57.7 511,58.2 511,58.7 511,59.2 511,59.7 513,68.9 513,69.4 513,69.9 513,70.4 513,70.9 513,71.9 514,80.7 514,81.2 514,81.7 514,82.2 514,82.7 514,83.7 514,84.7 514,85.7 567,73.6 567,74.1 567,74.6 567,75.1 567,75.6 567,76.6 723,79.1 723,79.6 723,80.1 723,80.7 723,81.1 723,82.1 724,79.1 724,80.7 724,78.6 724,79.6 725,80.9 725,81.4 725,81.9 725,82.5 725,82.9 733,57.1 733,57.6 733,58.1 733,59.1 889,72.5 889,73.5 889,74.5 889,73.0 906,65.3 906,65.8 906,66.3 906,67.3 906,66.8 906,68.3 906,69.3 906,72.3 906,71.3 906,70.3 922,68.1 922,68.6 922,69.1 922,69.6 922,70.1 922,71.1 922,72.1 922,74.1 922,73.1 1098,72.0 1098,72.5 1098,73.0 1098,74.0 1098,75.0 1098,75.9 1098,82.0 1098,80.0 1098,79.0 1098,78.0 1098,77.0 1098,83.0 1281,77.6 1281,78.1 1281,79.6 1281,78.6 1283,59.9 1283,60.4 1283,60.9 1283,61.9 1285,80.0 1285,80.5 1285,81.0 1285,82.0 1308,79.7 1308,80.2 1308,80.7 1308,81.7 5013,69.3 5013,68.9 5013,68.3 5017,85.3 5017,84.9 5017,84.3 5087,65.6 5087,65.1 5087,64.6 6497,66.5 6497,67.4 204,71.1 204,71.7 204,72.1 204,72.6 204,73.1 204,74.1 204,75.1 292,76.4 292,76.9 292,77.4 292,78.0 292,78.4 292,79.4 292,80.4 292,85.7 292,84.5 292,83.5 292,82.5 292,81.4 341,74.0 341,74.5 341,75.1 341,76.0 555,77.0 555,77.5 555,78.0 555,79.3 555,80.2 555,81.0 555,86.5 555,85.1 555,83.0 555,82.0 555,88.2 997,80.4 997,80.9 997,81.5 997,81.9 997,82.5 997,83.4 997,84.4 997,85.4 4414,65.0 4414,63.8 4414,62.8 4414,61.7 4414,61.3 4414,60.8 4414,66.3 4783,84.2 4783,83.6 4783,83.2 577,72.4 577,72.9 577,73.4 577,74.4 759,81.8 759,82.8 759,83.8 759,82.3 760,69.4 760,70.0 760,70.4 760,71.5 945,72.4 945,73.0 945,73.4 945,73.9 945,74.4 945,75.4 945,76.5 976,80.5 976,81.0 976,81.5 976,82.0 976,82.6 976,83.6 1001,69.3 1001,69.8 1001,70.3 1135,75.6 1135,76.1 1135,76.6 1135,77.1 1135,77.6 1240,67.0 1240,67.5 1240,68.0 1240,68.5 1240,69.0 4430,84.0 4430,82.0 4430,81.0 4430,80.5 4430,80.0 4430,85.7 4538,82.8 4538,80.9 4538,79.8 4538,79.3 4538,78.8 4538,84.3 4538,85.4 4715,60.7 4715,58.7 4715,57.7 4715,57.2 4715,56.7 4715,62.0 4820,86.6 4820,86.1 4820,85.6 4894,61.7 4894,61.2 4894,60.7 5063,72.5 5063,72.0 5063,71.5 6179,79.4 6179,80.6 150,73.9 150,74.4 150,74.9 150,75.4 150,75.9 150,76.9 150,83.9 150,82.1 150,79.4 150,85.4 539,73.4 539,73.9 539,74.4 539,74.9 539,75.3 539,76.5 539,77.3 552,60.1 552,60.6 552,61.1 552,61.6 552,62.1 552,65.1 566,78.8 566,79.3 566,79.8 566,80.3 566,82.1 566,80.8 566,87.8 566,85.8 566,85.1 588,66.8 588,64.6 588,65.1 588,65.6 588,66.1 588,70.2 588,69.2 627,58.9 627,59.4 627,58.4 1078,70.6 1078,71.1 1078,71.6 1078,72.3 1078,72.8 1078,73.8 1078,74.7 1078,79.6 1078,78.8 1078,76.5 1078,75.6 1225,76.9 1225,77.4 1225,76.4 1225,78.0 4001,89.0 4001,88.5 4030,72.3 4030,70.3 4030,69.3 4030,68.8 4030,68.3 4030,75.6 4030,74.6 4770,76.9 4770,76.4 4770,75.9 4879,80.6 4879,80.1 4879,79.6 6377,81.6 6377,82.6 549,68.7 549,69.2 549,69.7 549,70.3 898,83.1 898,83.6 898,84.1 898,85.2 898,86.1 1010,74.2 1010,74.7 1010,75.2 1010,75.7 1010,76.3 1010,77.2 1010,78.2 1368,75.2 1368,75.8 1368,76.2 1368,77.2 1412,59.4 1412,59.9 1412,60.4 1423,69.4 1423,69.9 1423,70.4 1425,76.1 1425,77.1 1425,78.6 1425,75.6 1425,76.6 1425,77.6 1425,79.6 4041,77.9 4041,78.4 4041,78.9 4041,79.8 4143,63.6 4143,64.1 4143,64.6 4143,65.6 4143,67.5 4143,69.5 4143,70.5 4271,65.8 4271,63.8 4271,62.8 4271,62.3 4271,61.8 4271,67.7 4271,68.9 4271,69.7 4974,75.4 4974,74.4 4974,73.9 4974,73.4 4974,78.2 1296,76.9 1296,77.4 1296,77.9 1296,78.9 1331,76.5 1331,78.1 1331,77.0 1331,77.6 1331,78.5 1331,80.7 1331,81.8 4980,88.3 4980,87.9 4980,87.3 5005,79.2 5005,78.6 5005,78.1 5285,71.4 5285,73.8

5285,75.8 5285,76.9 952,81.7 952,82.2 952,82.7 952,83.2 952,84.8 952,85.8 952,86.7 952,83.7 952,87.3 1054,69.9 1054,70.4 1054,71.4 1054,70.9 1054,71.9 1346,72.6 1346,73.2 1346,73.7 1346,74.2 1346,74.6 1346,75.8 1346,76.6 1346,80.6 1346,79.6 1346,78.6 1346,77.7 4959,78.5 4959,78.0 4959,77.5 5062,72.1 5062,71.5 5062,71.0 389,71.5 389,72.0 389,74.5 389,73.5 389,73.0 389,72.5 389,75.5 507,55.2 507,55.7 507,56.2 507,56.7 507,57.2 507,58.2 1044,65.6 1044,66.6 1044,66.1 1044,67.7 5070,72.2 5070,71.8 5070,71.2 474,77.8 474,79.2 474,77.3 474,78.3 941,73.3 941,73.8 941,74.3 941,74.8 941,75.3 1007,72.3 1007,72.8 1007,73.3 1007,73.7 1007,74.3 1007,75.3 1007,77.3 1217,67.8 1217,68.3 1217,68.8 1217,69.3 1217,69.8 1217,70.9 1371,84.2 1371,84.7 1371,85.2 1371,86.2 1373,75.4 1373,75.9 1373,77.4 1373,76.4 1379,88.2 1379,87.7 1379,88.7 1379,89.7 2398,71.5 2398,72.0 2398,75.5 2398,73.5 2398,72.5 535,76.8 535,77.3 535,77.8 535,78.8 690,80.1 690,80.6 690,79.6 690,81.6 730,71.1 730,71.6 730,72.6 730,70.6 793,68.9 793,69.4 793,69.9 793,71.1 29,64.1 29,64.6 29,65.1 29,66.6 76,78.3 76,78.8 76,79.3 76,81.1 77,79.7 77,80.2 77,80.7 77,81.3 77,82.1 98,84.4 98,85.2 98,85.4 98,87.4 110,82.6 110,83.6 243,68.0 243,69.0 336,76.1 336,76.6 336,77.1 336,78.1 812,71.5 812,72.0 812,73.5 1185,62.0 1185,62.5 1253,62.6 1253,63.1 1253,63.7 4728,82.9 4728,82.4 4728,81.9 109,70.1 109,70.6 109,71.1 109,72.2 442,74.7 442,75.4 442,75.8 872,69.6 872,70.2 872,70.7 872,71.4 872,72.0 872,74.6 2316,74.3 2316,74.8 2316,75.5 565,75.1 565,74.6 565,75.6 565,76.6 909,69.1 909,69.6 909,70.1 909,71.1 909,72.1 4853,71.9 4853,70.9 832,79.0 832,80.0 832,80.5 832,81.1 832,83.2 1079,78.0 1079,78.5 1079,79.0 1377,82.6 1377,83.1 1377,83.6 5029,80.1 5029,79.6 434,71.7 434,72.2 434,72.7 434,73.2 434,73.7 434,74.7 1015,58.7 1015,59.2 1027,69.6 1027,70.1 1027,69.1 1090,71.3 1090,71.8 1090,72.3 1102,72.8 1102,73.3 1164,69.5 1164,70.0 1164,70.5 1164,71.6 1397,55.1 1397,55.6 1397,56.1 1398,77.6 1398,78.1 1398,79.1 1398,78.6 1402,69.8 1402,70.3 1402,69.3 4089,75.7 4089,74.7 4089,74.0 4089,73.7 4282,90.1 4282,89.6 149,87.7 149,88.2 149,88.7 149,89.7 160,75.2 160,75.9 160,76.7 160,77.2 160,79.2 160,76.3 160,78.3 160,81.2 160,80.3 269,67.1 269,68.6 269,69.1 269,70.1 269,71.1 269,67.6 269,68.1 269,72.1 667,69.0 667,69.6 667,70.0 667,70.5 667,71.0 667,72.0 667,73.0 667,74.1 896,76.8 896,77.8 896,78.8 896,79.8 896,77.3 896,80.8 896,86.0 896,81.9 896,88.2 896,89.2 4201,64.5 4201,64.0 4215,83.6 4215,82.6 4215,82.1 4215,81.6 51,66.5 51,67.0 51,67.5 51,68.1 51,68.5 51,69.5 51,74.5 51,73.5 51,72.2 54,81.0 54,81.6 54,82.0 54,83.0 54,82.6 111,75.8 111,76.4 111,76.8 372,79.0 372,79.5 372,80.0 372,81.0 470,86.6 470,87.6 470,88.6 470,87.1 1144,77.4 1144,78.0 1144,78.4 1144,79.5 190,79.8 190,78.8 190,79.3 190,80.3 190,80.8 743,85.6 743,86.1 747,72.6 747,73.1 747,73.6 930,75.0 930,75.5 930,77.0 930,76.0 995,78.5 995,79.0 995,79.5 995,80.7 995,81.5 1062,81.9 1062,82.4 1062,83.9 1062,82.8 1113,79.2 1113,79.7 1154,76.6 1154,77.0 1226,83.1 1226,84.6 1226,85.7 1226,86.6 1226,82.6 1226,83.6 1226,84.1 1157,84.9 1157,85.4 1157,85.9 1157,86.9 374,75.9 374,76.4 374,76.9 374,77.9 378,69.0 378,69.5 378,70.0 378,70.5 378,71.0 378,72.0 378,76.6 378,75.7 378,74.6 979,86.2 979,86.7 979,87.2 979,88.2 4379,89.1 4379,88.6 4379,87.8 4404,86.5 4404,84.5 4404,83.5 4404,83.0 4404,82.5 4404,88.0 4404,89.6 4404,90.0 361,74.5 361,75.0 361,75.5 361,76.0 361,76.5 361,77.5 361,78.5 361,83.5 361,81.5 361,80.6 361,79.5 370,74.5 370,75.0 370,75.5 370,76.5 392,84.6 392,85.1 392,85.6 392,86.6 487,76.4 487,76.9 487,77.4 487,78.4 649,86.7 649,87.2 649,87.7 649,88.2 649,88.7 649,89.7 752,82.0 752,82.5 752,82.9 752,83.5 752,84.0 752,85.0 752,86.0 752,88.0 752,87.0 834,63.9 834,65.4 834,66.0 834,67.0 834,64.4 834,65.0 834,67.9 1243,77.3 1243,77.8 1243,78.3 1243,78.8 1243,80.3 1243,79.3 1243,81.3 1243,84.4 1243,83.3 1243,82.3 1271,71.2 1271,71.7 1271,72.2 1271,73.2 1271,74.2 1271,72.7 1271,76.2 1271,75.2 1315,83.3 1315,83.8 1315,84.3 1315,84.8 1315,85.3 1315,86.3 4195,64.2 4195,63.2 4195,62.7 4195,62.2 4338,81.5 4338,81.0 4338,80.5 4625,65.8 4625,64.8 4625,64.3 4625,63.8 4732,78.2 4732,77.7 4732,77.2 6100,71.3 6100,72.3 6100,73.4 6543,72.2 6543,73.1 1350,71.6 1350,72.0 50,79.1 50,79.6 50,78.1 50,77.6 50,78.6 50,80.6 88,65.6 88,66.1 88,66.6 91,62.4 91,62.9 91,63.4 91,64.4 94,70.7 94,71.2 94,72.7 94,71.7 106,73.2 106,73.7 106,74.2 106,75.2 106,76.2 106,77.2 106,78.2 106,82.2 106,81.2 106,80.2 106,79.2 106,84.6 106,85.9 108,78.2 108,79.2 108,79.7 108,80.2 108,81.2 108,83.2 108,82.2 108,78.7 108,84.2 162,71.8 162,72.3 162,72.8 162,73.8 390,81.4 390,81.9 390,80.4 390,80.9 390,82.4 4526,81.4 4526,80.4 4526,79.9 4526,79.4 606,68.5 606,69.0 606,69.5 606,70.5 708,76.4 708,76.9 708,77.4 708,77.9 708,78.4 708,79.4 708,80.4 784,75.8 784,76.3 784,76.8 784,77.8 891,79.3 891,81.3 891,79.8 891,80.3 1077,83.3 1077,83.8 1077,84.8 1077,85.3 1077,86.3 1221,71.2 1221,71.7 1221,72.2 1221,73.2 4494,73.1 4494,72.1 4494,71.6 4494,71.1 4507,80.1 4507,79.3 4507,78.7 4507,78.2 4507,84.5 4507,85.4 4686,72.7 4686,72.2 4686,71.7 4891,63.7 4891,61.7 4891,60.7 4891,60.2 4891,59.7 4891,65.6 4891,66.7 6683,66.7 6683,67.7 259,70.6 259,71.1 259,71.6 259,72.6 259,73.6 259,74.6 259,75.6 259,78.6 259,77.6 259,76.6 259,82.1 259,83.2 431,84.0 431,84.5 431,85.0 431,86.0 754,67.7 754,68.2 754,68.7 754,69.7 844,85.3 844,85.8 844,86.3 844,87.3 1382,64.3 1382,64.8 1382,65.3 1382,66.3 1427,69.6 1427,70.1 1427,70.6 1427,71.1 1427,71.6 1427,72.6 1427,78.6 1427,76.6 1427,75.6 1427,74.6 1427,73.6 1427,79.6 1427,80.7 1427,81.7 4197,84.4 4197,83.3 4197,81.3 4197,80.3 4197,79.8 4197,79.3 4197,85.4 4197,86.5 4500,92.2 4500,91.4 4500,90.8 4500,90.3 4765,80.0 4765,78.0 4765,77.0 4765,76.5 4765,76.0 4765,81.0 4928,81.8 4928,79.8 4928,79.0 4928,78.3 4928,77.8 4928,82.7 4940,90.3 4940,89.8 4940,89.3 4992,64.7 4992,64.2 4992,63.7 5028,62.9 5028,62.4 5028,61.9 5056,86.4 5056,85.8 5056,85.3 5058,62.8 5058,62.3 5058,61.8 5067,80.6 5067,81.1 5067,81.6 5095,66.9 5095,66.4 5095,65.9 6433,76.4 6433,77.5 6549,66.1 6549,67.1 216,84.8 216,84.3 216,85.3 216,86.3 227,79.6 227,80.1 227,80.6 227,81.2 227,81.6 227,83.0 227,84.2 227,87.6 227,86.7 227,85.6 230,80.0 230,80.6 230,81.0 230,82.0 230,83.5 230,87.8 230,86.8 230,85.8 258,71.3 258,71.8 258,72.3 258,72.8 258,73.3 266,85.5 266,86.0 266,86.5 266,87.5 310,82.7 310,83.2 310,83.7 517,81.8 517,82.3 517,82.8 528,70.8 528,71.3 528,71.8 528,72.8 611,71.9 611,72.5 611,73.4 611,72.9 611,75.2 740,72.2 740,72.7 740,73.2 947,82.6 947,83.1 947,83.6 947,84.1 1043,68.5 1043,69.0 1043,69.5 1043,70.0 1043,71.5 1043,73.4 1407,74.6 1407,75.2 1407,76.8 1407,77.9 1407,81.1 1407,80.2 1407,79.1 1409,65.9 1409,66.5 1409,67.5 1430,83.9 1430,83.4 1430,85.4 4772,79.7 4772,78.7 4774,86.8 4774,86.2 4774,85.8 4792,80.8 4792,80.3 5123,74.5 5123,73.5 4422,74.8 4422,72.8 4422,71.8 4422,71.4 4422,70.8 4422,76.0 4422,77.0 289,67.7 289,68.7 289,69.2 289,69.7 289,71.7 289,68.2 289,72.8 289,73.7 956,64.3 956,64.8 956,63.8 956,65.8 1201,76.0 1201,76.5 1201,75.5 1290,79.3

1290,79.8 1290,80.3 1337,71.4 1337,71.9 1337,72.4 2373,79.0 2373,79.5 2373,83.0 2373,82.0 2373,81.0 2373,80.0 2373,85.1 2373,86.1 2373,87.1 2403,79.6 2403,79.1 2403,81.1 2403,80.1 2403,84.8 2403,86.9 4294,79.2 4294,77.1 4294,76.2 4294,75.6 4294,75.2 4294,81.0 4294,82.5 4294,83.1 4415,79.1 4415,77.1 4415,76.2 4415,75.6 4415,75.1 4415,80.8 4589,75.7 4589,75.1 4641,75.6 4641,74.6 4641,74.1 4641,73.6 4660,79.2 4660,78.2 4660,77.7 4660,77.2 4730,82.1 4730,81.6 4730,81.1 4971,76.9 4971,76.4 4982,59.2 4982,58.7 4982,58.2 4984,74.0 4984,73.5 4984,73.1 4990,75.4 4990,75.1 5006,69.2 5006,68.7 5006,68.2 5059,72.5 5059,72.1 6072,89.2 6072,90.2 6072,91.2 457,82.7 457,83.2 457,83.7 457,84.8 497,75.7 497,76.2 497,76.7 497,77.8 691,64.3 691,64.8 1389,76.1 1389,76.6 1389,77.1 1389,77.6 1389,78.2 1389,79.1 987,79.1 987,79.6 987,80.2 638,83.1 638,83.6 638,84.1 638,84.6 638,85.1 638,86.1 727,69.2 727,69.7 727,71.2 727,70.7 727,70.2 727,72.2 913,56.6 913,57.1 913,57.6 913,58.1 1055,84.6 1055,85.1 1055,85.7 1170,72.9 1170,73.4 1170,73.9 1170,75.1 4657,73.5 4657,72.5 4657,72.0 4657,71.5 4676,78.8 4676,79.3 4676,79.8 4676,80.8 4863,71.0 4863,70.5 4863,70.0 4954,62.1 4954,61.6 4954,61.1 5015,78.8 5015,78.3 5015,77.8 6284,80.1 6284,81.1 6389,69.7 6389,70.7 6545,61.4 6545,62.4 194,80.5 194,81.0 194,81.4 195,79.6 195,80.1 195,80.6 195,81.1 195,82.6 195,81.6 299,89.1 299,89.6 299,90.1 299,91.1 300,56.4 300,56.9 300,57.4 300,58.4 426,79.6 426,80.1 426,80.6 426,81.6 695,74.3 695,74.8 695,77.3 695,78.4 695,75.3 695,76.3 873,78.1 873,79.1 873,79.6 873,80.1 873,78.6 873,82.1 874,66.1 874,66.6 874,67.1 874,67.6 874,69.2 874,68.2 366,56.5 366,57.0 366,57.5 366,58.5 631,77.1 631,77.6 631,78.1 631,78.6 631,80.1 631,79.1 796,80.3 796,80.8 796,81.3 796,82.7 841,77.5 841,77.0 973,76.8 973,77.3 973,77.8 973,78.3 973,78.8 973,80.8 973,79.8 994,55.1 994,55.6 994,56.1 994,56.6 994,58.1 994,59.1 994,57.1 994,63.1 994,62.1 994,61.0 994,60.1 1041,70.9 1041,71.4 1041,71.9 1041,72.9 4211,82.9 4211,81.9 4211,81.4 4211,80.9 4258,77.8 4258,76.8 4258,76.3 4258,75.8 4631,73.5 4631,71.5 4631,70.5 4631,70.0 4631,69.5 4631,76.1 4672,68.8 4672,67.8 4672,67.3 4672,66.8 4756,85.2 4756,84.7 4756,84.2 4862,79.3 4862,77.3 4862,82.3 4862,83.3 696,75.0 696,74.0 696,74.5 790,83.3 790,83.8 790,84.4 852,84.3 852,84.8 852,85.3 853,86.8 853,87.3 853,87.8 915,80.6 915,81.2 915,81.6 915,82.1 915,87.6 915,86.6 982,80.2 982,81.2 982,81.7 1004,73.9 1004,74.4 1004,74.9 1004,75.4 1004,80.9 1004,79.9 1004,79.0 1024,68.6 1024,69.6 1137,81.5 1137,82.0 1137,82.5 1152,71.4 1152,71.9 1152,72.3 1244,78.9 1244,79.4 1255,76.4 1255,77.5 1255,80.4 1255,76.9 1255,78.5 1255,81.4 4172,77.8 4172,76.8 4172,76.3 4172,75.8 6142,86.1 6142,87.5 6142,88.2 6634,80.3 6634,81.3 6592,71.8 6592,72.8 1295,76.7 1295,77.2 1295,78.2 1311,69.1 1311,69.6 1311,70.2 1311,70.6 1311,71.1 6345,79.3 6345,80.3

For the A$\beta$+ MCI cohort:
ID,TIME 782,81.6 782,82.7 782,82.2 782,83.4 782,83.8 782,84.6 1261,71.1 1261,73.0 1261,71.6 1261,72.4 1261,74.1 1261,79.4 1261,78.1 1261,77.1 1261,76.1 1261,81.1 1261,75.1 1261,82.1 1261,83.1 1268,83.2 1268,83.9 1268,84.2 1268,84.7 1268,85.7 1268,86.8 1268,82.7 1268,87.7 2010,62.9 2010,63.4 2010,63.9 2010,64.9 2010,65.9 2010,67.9 2043,72.2 2043,73.2 2073,63.4 2073,63.9 2073,69.4 2073,67.4 2073,66.4 2073,65.4 2073,64.4 4171,70.4 4171,69.9 4171,69.4 4219,80.4 4219,79.9 4219,79.4 4229,70.4 4229,68.4 4229,67.4 4229,66.9 4229,66.4 4229,72.4 4229,73.4 4229,74.5 4251,72.4 4251,71.9 4270,78.7 4270,76.6 4270,75.6 4270,75.1 4270,74.6 4447,70.5 4447,69.6 4447,68.5 4447,68.0 4447,67.5 4473,78.8 4473,77.8 4473,76.8 4473,75.8 4473,75.3 4473,74.8 4473,79.9 4654,75.4 4654,75.9 4654,76.4 4654,77.4 4654,79.4 4654,80.4 4654,81.5 4654,82.5 4746,72.2 4746,71.7 4746,71.2 4799,72.0 4799,70.0 4799,69.0 4799,68.5 4799,68.0 4799,72.9 4799,73.9 4799,74.9 908,62.9 908,63.4 908,63.9 908,64.4 908,64.9 908,65.9 908,67.0 908,70.1 908,69.2 908,68.1 908,73.9 908,75.1 908,75.9 4288,74.6 4288,73.5 4288,73.0 4288,72.5 4288,78.4 4288,80.3 6258,79.8 6258,80.8 6268,69.6 6268,70.6 6432,67.3 6432,68.3 6606,73.6 6606,74.6 324,75.3 324,75.8 324,76.3 324,76.8 324,77.3 324,78.4 2390,87.8 2390,88.3 2390,91.8 2390,89.8 2390,88.8 4168,86.2 4168,84.2 4168,83.2 4168,82.8 4168,82.2 4185,84.4 4185,82.4 4185,81.4 4185,80.9 4185,80.4 4185,86.4 4185,87.4 4346,72.3 4346,71.8 4346,71.3 4363,75.6 4363,74.5 4363,74.0 4363,73.5 4679,71.1 4679,70.2 4679,69.6 4679,69.1 4713,74.5 4713,72.5 4713,71.5 4713,71.0 4713,70.5 4713,77.2 6291,72.3 6291,73.4 6610,61.3 6610,62.3 414,79.6 414,80.1 414,80.6 414,81.1 414,81.6 698,74.3 698,74.9 698,75.3 698,75.9 698,76.3 698,77.3 698,78.3 698,79.5 2394,68.0 2394,67.4 2394,71.4 2394,69.4 2394,68.4 2394,73.5 2394,74.5 4272,74.9 4272,72.9 4272,71.9 4272,71.4 4272,70.9 4272,77.1 4272,78.1 4467,63.9 4467,62.9 4467,62.4 4467,61.9 4611,70.9 4611,69.0 4611,67.9 4611,67.4 4611,66.9 6341,67.1 6341,68.1 842,73.6 842,74.1 842,74.6 842,77.7 842,75.6 842,76.6 842,81.6 842,80.7 842,79.7 842,78.6 4359,80.8 4359,78.7 4359,77.8 4359,77.2 4359,76.7 4388,70.9 4388,68.8 4388,67.9 4388,67.3 4388,66.8 4388,72.3 4903,78.2 4903,77.7 4903,77.2 5000,77.6 5000,77.2 5000,76.6 161,79.1 161,79.6 168,89.8 168,89.3 4235,67.8 4235,65.8 4235,64.8 4235,64.3 4235,63.8 4278,79.0 4278,77.0 4278,76.0 4278,75.5 4278,75.0 4278,81.1 4278,82.3 917,70.2 917,70.7 932,75.3 932,75.8 932,76.3 932,76.8 932,77.3 1009,75.8 1009,76.3 1009,76.8 1009,77.8 1165,81.4 1165,81.9 1165,82.9 1165,83.4 1165,82.5 1175,72.7 1175,73.2 1175,74.2 1175,74.7 4012,72.9 4012,70.9 4012,70.0 4012,69.4 4012,68.9 4128,76.9 4128,74.8 4128,73.8 4128,73.3 4128,72.8 4849,66.9 4849,66.4 4849,65.9 1120,77.9 1120,78.4 1120,79.1 1120,79.4 1275,78.9 1275,79.4 1275,79.9 1275,80.6 2324,56.3 2324,56.8 2324,57.3 2389,57.8 2389,58.4 2389,59.0 2389,64.2 4985,80.7 4985,79.7 4985,79.2 4985,78.7 169,72.9 169,73.4 169,75.9 169,71.9 169,72.4 169,73.9 169,74.9 169,76.9 169,77.9 520,81.9 520,77.7 520,78.2 520,78.7 520,79.7 520,80.7 520,84.4 520,82.7 557,72.0 557,72.5 557,73.0 557,73.5 557,74.1 557,75.0 558,79.8 558,80.3 558,80.8 558,82.8 558,83.8 558,81.8 4577,85.2 4577,84.7 6199,69.1 6199,70.2 354,76.9 354,75.8 354,76.3 1028,76.9 1028,77.4 1028,77.9 1028,78.4 1028,78.9 1092,74.3 1092,74.8 1092,76.3 1092,77.3 1092,75.3 1092,75.8 1138,67.4 1138,67.9 2007,83.4 2007,83.8 2007,85.1 2007,84.1 2284,76.6 2284,77.1 4646,62.8 4646,61.8 4646,61.3 4646,60.8 5007,73.5 5007,72.5 5007,72.1 5007,71.5 55,76.0 55,76.6 55,78.2 55,80.3 55,85.0 55,83.0 55,81.8 80,85.0 80,85.5 80,86.0 87,75.1 87,75.7 87,76.1 87,76.6 103,87.3 103,87.8 2138,81.6 2138,82.3 2155,68.9 2155,69.4 2155,72.9 2155,71.9 2155,70.9

2155,69.9 2155,75.8 2155,76.6 2155,77.7 4400,75.1 4400,73.2 4400,72.1 4400,71.7 4400,71.1 4400,76.8 4400,77.8 4400,78.8 4597,69.5 4597,68.5 4809,82.3 4809,80.3 4809,79.3 4809,78.8 4809,78.3 4868,81.1 4868,79.1 4868,78.1 4868,77.6 4868,77.1 4868,82.1 4868,83.1 4889,77.6 4889,76.6 4889,76.1 4889,75.6 6315,71.2 6315,72.2 6483,62.6 6483,63.6 6635,56.5 6635,57.5 883,76.1 883,76.7 883,77.2 883,78.3 883,79.2 899,80.1 899,80.6 899,81.2 899,82.2 899,83.2 4920,62.9 4920,62.4 4920,61.9 178,64.8 178,75.9 178,78.0 2077,81.0 2077,81.4 2077,85.0 2077,83.0 2077,82.0 2100,87.8 2100,88.3 2100,91.8 2100,90.8 2100,89.8 2100,88.8 2125,78.1 2125,78.6 2125,81.1 2125,80.1 2125,79.1 2142,82.7 2142,83.2 2142,84.7 2142,83.7 2150,73.2 2150,72.7 4254,89.9 4254,87.9 4254,86.9 4254,86.4 4254,85.9 4254,91.4 4254,92.5 4419,65.8 4419,64.8 4419,64.3 4419,63.8 4744,76.3 4744,74.3 4744,73.3 4744,72.8 4744,72.3 4744,77.4 4744,78.4 5237,82.3 5237,80.3 5237,85.4 5237,86.4 6312,80.3 6312,81.3 44,86.3 44,85.8 544,76.7 544,77.2 544,77.8 544,78.2 544,78.7 961,73.3 961,73.8 961,74.3 961,74.8 961,75.8 961,72.8 2167,81.2 2167,80.7 2167,85.7 2167,83.7 2167,82.9 2167,81.9 2263,62.5 2263,62.0 2263,66.0 2263,65.0 2263,64.1 2263,63.0 2263,68.2 2263,70.2 2379,66.0 2379,66.5 2379,70.2 2379,68.0 2379,67.0 2379,71.9 2379,73.9 4444,81.5 4444,79.6 4444,78.5 4444,78.0 4444,77.5 4805,73.9 4805,72.9 4805,72.6 4805,71.9 4922,77.2 4922,76.9 4922,76.2 1046,71.6 1046,72.1 1046,72.6 1046,73.1 1046,74.6 1046,75.8 1046,73.8 1046,76.7 1104,64.5 1104,65.0 2068,84.1 2068,83.6 2068,83.1 2068,86.2 2068,85.1 2068,90.1 4034,73.1 4034,72.4 4034,72.1 4241,70.8 4241,70.3 4241,69.8 6356,68.7 6356,69.7 6535,78.2 6535,79.2 1063,78.4 1063,78.9 1063,79.4 1063,80.4 1063,81.4 1063,82.3 2239,73.5 2239,74.0 2239,77.4 2239,76.5 2239,74.5 2239,75.4 2239,80.8 2239,82.0 4392,84.4 4392,83.4 4392,82.9 4392,82.4 4674,81.9 4674,79.9 4674,78.9 4674,78.4 4674,77.9 4674,83.0 4674,84.0 4674,85.0 116,78.4 116,78.9 116,79.4 116,79.9 116,82.4 116,83.4 116,80.4 116,81.4 116,84.4 116,85.4 307,78.5 307,79.0 307,79.5 307,80.0 307,80.5 307,81.5 307,82.5 307,83.5 307,85.7 307,84.6 417,79.8 417,80.3 417,80.8 485,64.4 485,64.9 485,65.4 485,65.9 644,75.0 644,75.5 644,76.0 644,76.5 644,77.0 644,78.0 644,79.0 644,80.0 1277,86.3 1277,86.8 1277,87.3 2219,74.3 2219,74.7 2219,75.3 2219,76.3 2219,77.3 2219,78.3 2219,80.4 2219,81.4 2219,82.4 2219,83.4 2245,57.0 2245,57.4 2245,58.0 2245,62.0 2245,61.0 2245,60.0 2245,59.0 2245,63.0 2245,64.0 2245,65.0 2245,66.0 2336,72.9 2336,73.9 2336,73.4 2336,78.0 2336,76.9 2336,75.9 2336,74.9 4804,80.4 4804,79.9 4804,79.4 4919,78.0 4919,76.8 4919,75.8 4919,75.3 4919,74.8 4919,79.0 4919,80.0 4919,81.1 4919,82.2 4926,65.5 4926,64.5 4926,63.5 4926,63.0 4926,62.5 4955,72.4 4955,72.0 4955,71.4 5277,73.8 5277,71.8 5277,75.8 6034,63.9 6034,65.9 6370,69.8 6370,71.0 1038,83.2 1038,83.7 1218,86.3 1218,87.5 1218,85.4 1218,88.3 1218,89.3 1384,73.0 1384,72.0 1384,72.5 1384,73.6 1384,74.1 1384,75.1 2376,81.9 2376,82.3 2376,83.9 2376,82.9 4290,75.4 4290,74.5 4290,74.1 4290,73.5 4290,80.8 4327,83.9 4327,83.4 4327,82.9 4652,82.3 4652,80.3 4652,79.8 4652,79.3 5135,79.0 5135,77.0 5219,77.9 5219,75.7 5219,81.3 5219,82.3 351,68.4 351,68.9 351,69.9 351,70.3 351,69.4 351,71.4 2022,66.0 2022,66.5 2022,69.0 2022,68.0 2022,66.9 2233,55.5 2233,55.9 2233,58.7 2233,57.5 2233,56.5 2233,63.6 4029,63.1 4029,62.1 4029,61.6 4029,61.1 4474,86.1 4474,85.6 4590,61.2 4590,60.7 4590,60.2 4721,86.4 4721,85.9 4721,85.4 718,80.4 718,80.9 718,81.4 718,82.4 718,82.0 718,83.4 2119,71.7 2119,72.2 2119,72.7 2119,73.7 2119,74.7 2119,76.7 2119,78.3 2240,78.0 2240,78.4 2240,79.2 2247,65.1 2247,65.6 2247,70.2 2247,67.8 2247,66.3 4277,71.4 4277,72.0 4277,72.4 4277,73.4 4277,77.1 4277,78.1 4277,79.1 6279,89.1 6279,90.2 739,82.8 739,83.3 739,83.8 739,84.9 920,80.0 920,80.5 920,81.0 920,82.0 920,84.0 920,83.0 920,88.1 920,87.0 920,86.0 1116,83.4 1116,83.9 1116,84.4 1116,84.9 1279,68.3 1279,68.8 1279,69.3 1279,69.8 1279,70.3 1284,85.9 1284,86.4 1284,86.9 1309,69.1 1309,69.6 1309,70.1 1309,70.6 1309,71.1 33,84.3 33,86.4 33,83.3 33,83.8 33,84.8 33,85.3 4256,63.9 4256,63.3 4256,62.8 656,82.5 656,83.0 656,83.5 656,84.0 656,84.5 656,85.5 673,78.2 673,78.7 673,79.2 673,79.7 673,80.3 673,81.2 673,82.5 748,79.6 748,80.1 748,80.6 748,81.1 748,81.6 2380,67.8 2380,68.3 2380,73.5 2380,71.8 2380,69.8 2380,68.8 2380,74.7 2380,75.6 4562,65.6 4562,64.6 4562,64.1 4562,63.6 4736,73.8 4736,72.8 4736,72.3 4736,71.8 377,79.3 377,80.3 377,80.8 377,81.3 377,82.5 377,79.8 377,84.0 377,89.3 377,87.0 377,85.0 377,90.1 377,91.1 377,92.3 454,81.8 454,82.3 454,82.8 454,83.8 454,84.8 454,87.4 454,88.4 454,89.6 454,90.9 454,93.0 1421,74.4 1421,74.9 1421,75.4 1421,77.5 1421,76.1 4071,88.8 4071,86.5 4071,85.5 4071,85.0 4071,84.5 4071,90.2 4146,65.8 4146,63.8 4146,62.8 4146,62.3 4146,61.8 4214,78.2 4214,76.2 4214,75.2 4214,74.7 4214,74.2 4214,79.6 4214,81.2 4302,80.1 4302,78.2 4302,77.1 4302,76.6 4302,76.1 4302,81.3 6083,72.1 6083,73.0 6141,60.1 6141,61.1 6144,65.0 6144,67.2 6222,73.9 6222,75.0 282,72.1 282,72.8 282,73.1 282,73.7 679,62.9 679,63.4 679,63.9 679,64.4 679,64.9 679,65.9 679,66.9 679,71.9 679,69.9 679,68.9 679,67.9 679,73.8 679,74.8 679,75.6 721,66.3 721,66.8 1420,81.3 1420,81.8 4510,70.4 4510,68.4 4510,67.4 4510,66.9 4510,66.4 4510,72.0 4513,64.6 4513,62.6 4513,61.6 4513,61.1 4513,60.6 4513,66.3 4513,68.3 4629,71.2 4629,70.7 4720,79.4 4720,78.4 4720,77.9 4720,77.4 4877,86.4 4877,85.4 4877,84.9 4877,84.4 5097,69.5 5097,68.5 5097,67.5 5097,72.3 5097,73.6 5097,74.3 1040,71.4 1040,71.9 1040,72.4 1131,86.4 1131,86.9 1131,87.4 1131,87.9 1131,88.5 1131,89.5 1131,91.0 4929,76.7 4929,76.4 4929,75.7 989,64.3 989,64.8 989,65.9 989,66.4 989,67.5 989,69.3 989,68.4 989,69.9 1168,81.2 1168,81.7 1168,82.2 1168,82.7 1168,83.2 2249,75.8 2249,76.3 2249,76.8 2249,77.8 2249,79.8 4944,71.1 4944,70.0 4944,69.1 4944,68.5 4944,68.0 4944,73.2 4944,74.5 621,62.3 621,64.3 621,63.8 621,62.8 621,63.3 621,65.3 919,62.4 919,62.9 919,63.4 919,64.0 919,64.4 919,65.4 919,66.4 919,67.4 919,71.4 919,69.5 919,68.5 2396,70.2 2396,70.7 2396,71.2 2396,72.2 2396,73.2 2396,75.3 2396,77.7 4557,85.3 4557,84.3 4557,83.8 4557,83.3 4813,67.6 4813,68.1 4813,68.6 4813,69.6 4813,71.6 4813,73.8 4813,74.9 779,79.5 779,80.0 779,80.5 779,81.5 779,82.5 1265,82.2 1265,82.7 1265,83.2 1265,83.7 1265,84.2 1269,73.4 1269,73.9 1269,74.4 1269,74.9 1269,75.4 1269,76.5 1269,77.4 1269,78.4 4897,76.8 4897,76.3 4897,75.8 4909,79.2 4909,78.7 4909,78.2 1182,76.0 1182,76.5 1182,77.0 1182,77.5 1182,78.1 1182,79.0 1294,79.2 1294,79.7 38,76.8 38,77.3 38,77.8 45,85.9 45,86.4 176,74.7 176,75.3 176,75.7 176,76.8 176,77.7 284,76.8 284,77.4 284,77.8 290,75.9 290,75.2 290,76.3 290,77.2 290,78.2 607,78.0 607,78.6 607,79.8 607,80.3 607,79.1 2196,68.2 2196,68.7 2196,70.2 2196,69.2 2301,65.7 2301,66.2 2301,66.7 2301,67.7 2301,68.7 2301,69.7 2301,72.1 2301,74.1 4054,70.3

4054,69.3 4054,68.8 4054,68.3 4310,66.3 4310,64.3 4310,63.3 4310,62.8 4310,62.3 4767,70.4 4767,68.4 4767,67.4
4767,66.9 4767,66.4 4767,71.4 4767,72.4 4767,73.4 4782,75.9 4782,73.9 4782,72.9 4782,72.3 4782,71.9 4782,77.0
4782,78.0 4782,79.1 6474,73.8 6474,74.8 6529,60.3 6529,61.6 210,72.4 210,73.0 210,73.4 210,74.8 210,75.6 210,76.6
210,83.1 210,78.5 210,84.1 802,80.9 802,81.5 802,82.5 802,83.2 802,85.1 802,89.8 802,88.2 802,87.1 802,91.7 802,93.4
1075,67.1 1075,68.3 1075,68.8 2171,65.3 2171,65.9 2171,66.4 2184,80.4 2184,81.0 2184,85.0 2184,81.4 2184,87.3
2184,89.3 2194,71.0 2194,71.5 4061,69.5 4061,66.1 4061,65.1 4061,64.6 4061,64.1 4061,70.4 4061,72.5 4067,68.1
4067,66.4 4067,65.0 4067,71.1 4134,69.9 4134,68.8 4274,64.1 4274,63.0 4332,72.6 4332,69.0 4332,74.6 4332,75.7
4332,76.8 4431,77.4 4431,74.1 4431,79.7 4431,81.0 4793,85.8 4793,85.3 1211,86.7 1211,87.2 1211,88.7 1211,89.7
1380,84.7 1380,85.2 1380,86.2 1380,86.9 1380,87.7 1380,88.7 2037,75.8 2037,76.3 2037,79.8 2037,78.8 2037,77.8
2037,76.8 2164,71.3 2164,70.9 2164,75.1 2164,73.9 2164,72.9 2164,71.9 4007,80.1 4007,79.1 4007,78.7 4007,78.1
4226,62.9 4226,63.4 4226,63.9 4226,64.9 4226,65.9 4462,73.3 4462,72.3 4462,71.8 4462,71.3 2190,82.4 2190,82.9
2190,84.5 2190,83.4 2225,70.4 2225,70.9 2225,74.5 2225,73.5 2225,72.5 2225,71.4 2264,69.3 2264,69.9 2264,73.3
2264,72.3 2264,71.4 2264,70.3 4259,65.0 4259,64.0 4259,63.5 4259,63.0 4300,82.7 4300,81.5 4300,81.2 4300,80.6
4311,76.6 4311,74.5 4311,73.4 4311,73.0 4311,72.5 4312,73.4 4312,71.3 4312,70.2 4312,69.8 4312,69.3 4614,72.1
4614,69.7 4614,68.6 4614,68.1 4614,67.7 4986,87.3 4986,86.3 4986,85.3 5227,73.2 5227,71.3 928,82.8 928,83.5 928,83.8
928,84.5 1119,78.1 1119,76.4 1119,77.0 1119,77.5 2121,67.8 2121,68.3 2121,71.9 2121,70.8 2121,69.8 2121,68.8 2121,75.1
2307,78.6 2307,79.1 2307,79.7 4244,93.4 4244,92.4 4244,91.9 4244,91.4 5014,77.5 5014,77.0 5014,76.5 531,74.3 531,75.0
531,75.3 531,75.8 921,71.4 921,72.0 921,72.4 921,73.0 921,73.4 921,74.4 1188,84.2 1188,81.2 1188,81.7 1188,82.2
1188,82.7 1188,83.2 1241,72.1 1241,72.6 1241,73.1 1241,74.1 1241,75.1 1293,77.1 1293,77.6 1293,78.1 1293,78.6
1293,79.1 1314,80.7 1314,82.7 1314,81.7 1314,82.2 1314,83.7 1330,62.7 1330,61.7 1330,63.2 1330,63.7 1417,63.6
1417,64.1 1417,64.7 1417,63.1 1417,66.2 4858,59.1 4858,57.0 4858,56.0 4858,55.5 4858,55.0 2079,65.5 2079,65.9
2079,71.7 2079,69.5 2079,68.5 2079,67.5 2079,66.5 4059,73.1 4059,72.4 4059,71.9 60,70.0 60,70.5 60,71.0 291,79.4
291,79.9 291,80.4 291,80.9 291,81.4 291,82.4 291,85.4 551,64.2 551,64.7 551,65.3 551,66.3 551,67.3 958,64.3 958,64.8
1034,75.1 1034,75.6 1034,76.1 1034,76.6 1034,77.1 1034,78.1 1034,79.1 1034,80.2 2042,69.5 2042,70.0 2042,71.5
2042,70.5 2063,68.3 2063,68.8 2063,72.4 2063,71.3 2063,70.3 2063,69.3 2205,84.7 2205,85.2 2205,86.7 2205,85.7
4022,87.4 4022,86.9 4022,86.4 4157,83.1 4157,82.1 4157,81.6 4157,81.1 4205,83.5 4205,82.4 4205,81.9 4205,81.4
4480,65.8 4480,64.8 4480,64.3 4480,63.8 4498,81.5 4498,79.5 4498,78.4 4498,78.0 4498,77.5 4565,84.4 4565,83.3
4565,82.9 4565,82.4 6632,74.5 6632,75.5 6,80.4 6,80.9 6,81.4 6,81.9 6,83.4 6,82.3 296,79.1 296,79.6 296,80.1 296,82.1
296,84.1 296,80.6 296,81.1 296,89.9 296,87.1 296,85.1 5096,82.4 5096,81.4 5096,80.4 950,73.5 950,74.0 950,74.5 950,75.0
950,75.5 1114,79.3 1114,78.3 1114,78.8 1114,79.8 1114,80.3 1343,64.7 1343,65.2 1343,65.7 4531,77.7 4531,76.7 4531,75.7
4531,74.7 4531,74.2 4531,73.7 173,73.2 173,73.7 173,74.2 173,75.2 173,76.2 173,83.2 173,81.2 173,80.2 173,79.2 410,61.3
410,61.8 410,62.3 410,62.8 458,82.9 458,83.4 458,83.9 458,84.4 1103,83.0 1103,83.5 1103,84.0 1103,84.5 2392,64.9
2392,64.4 2392,68.4 2392,66.5 2392,65.4 2392,70.4 2392,71.6 2392,72.5 5047,71.8 5047,70.8 5047,69.8 5047,69.3
5047,68.8 5047,73.5 5234,80.6 5234,78.6 5234,82.8 5234,84.1 890,59.7 890,60.2 4092,85.5 4092,83.5 4092,82.5 4092,82.0
4092,81.5 4175,79.5 4175,77.5 4175,76.4 4175,76.0 4175,75.5 4635,76.8 4635,74.7 4635,73.7 4635,73.2 4635,72.7
4855,88.1 4855,85.8 4855,84.8 4855,84.3 4855,83.8 4855,89.3 4855,90.3 4855,91.3 4898,77.2 4898,76.2 4898,75.7
4898,75.2 1322,71.7 1322,72.1 1322,72.7 1300,73.4 1300,73.9 1300,74.4 1300,74.9 1300,75.4 1300,76.4 1300,77.4
1300,81.4 1300,80.4 1300,79.4 1300,78.4 1300,83.6 2055,75.2 2055,75.7 2055,79.3 2055,78.2 2055,77.2 2055,76.2
4127,65.2 4127,64.1 4127,63.6 4127,63.1 4127,70.2 4127,71.3 4170,69.6 4170,67.7 4170,66.6 4170,66.1 4170,65.6
4170,71.7 4170,73.1 4170,73.7 4780,72.2 4780,71.2 4780,70.6 4780,70.2 4806,56.4 4806,55.9 4806,58.0 4904,66.8
4904,66.2 4904,65.7 709,83.8 709,84.3 709,84.8 709,85.3 709,86.8 709,88.8 709,85.8 709,87.8 709,90.8 709,89.8 2360,63.5
2360,67.1 2360,65.0 2360,64.0 4743,71.1 4743,70.6 4743,70.1 393,86.2 393,86.7 393,87.2 393,87.7 397,74.6 397,75.1
622,74.5 622,75.0 622,75.5 622,76.5 622,77.5 622,78.5 1032,84.7 1032,85.7 1032,86.7 1032,87.7 1032,85.2 1032,86.2
1032,88.7 1032,90.7 1419,77.9 1419,78.4 1419,78.9 1419,79.4 1419,79.9 1419,80.9 1419,81.9 4198,82.4 4198,80.4
4198,79.4 4198,78.9 4198,78.4 4198,84.4 4198,85.5 4301,79.6 4301,78.6 4301,76.6 4301,75.6 4301,75.1 4301,74.6
4301,80.5 4624,81.6 4624,79.8 4624,78.7 4624,78.1 4624,77.6 4844,87.3 4844,86.4 4844,85.3 5132,71.3 5132,70.3
5132,69.3 5132,73.3 5132,75.3 6173,70.4 6173,72.4 6241,72.2 6241,73.4 6512,78.2 6512,79.2 138,86.3 138,86.8 138,87.3
188,86.1 188,86.6 188,87.6 188,87.1 188,88.1 188,89.7 229,74.4 229,75.0 229,75.4 229,76.4 229,77.9 229,78.9 229,84.4
229,82.4 229,81.4 229,80.3 522,69.9 522,70.4 522,70.9 522,71.9 522,73.2 522,77.4 522,76.4 545,71.8 545,72.3 545,72.8
545,73.8 545,74.9 545,76.0 545,81.8 545,79.9 545,78.8 545,77.8 608,68.4 608,68.9 608,69.4 608,69.9 608,70.6 608,71.7
715,80.0 715,80.5 715,81.0 770,71.7 770,72.2 770,72.9 770,71.2 770,74.2 1148,69.7 1148,70.2 1148,70.7 1406,61.1
1406,62.2 1406,64.1 1406,61.6 1406,63.1 2045,72.3 2045,72.7 2045,76.3 2045,75.3 2045,74.3 2045,73.3 4553,70.5
4553,69.5 4553,69.0 4553,68.5 4636,81.1 4636,79.1 4636,78.1 4636,77.1 4653,82.4 4653,81.4 4653,80.9 4653,80.4
4742,74.6 4742,72.6 4742,71.6 4742,71.1 4742,70.6 4742,76.8 4742,77.8 5066,81.7 5066,80.8 5066,80.2 5066,79.7
1204,77.3 1204,77.8 1246,72.4 1246,72.9 1246,73.9 1246,73.5 1246,74.4 1246,75.4 1246,79.4 1246,78.4 1246,77.5
1246,76.5 4073,77.3 4073,76.7 4073,76.2 4287,75.0 4287,72.9 4287,71.9 4287,71.3 4287,70.9 102,71.3 102,71.8 102,70.3
102,70.8 102,72.3 232,78.0 232,78.5 232,79.0 232,82.0 232,80.0 232,83.0 232,84.1 285,65.6 285,66.6 285,67.1 285,67.6
285,69.6 285,66.1 285,70.6 285,71.6 449,66.5 449,67.1 449,68.1 449,68.6 449,67.7 783,79.2 783,79.7 783,80.7 783,81.2
783,80.2 2391,71.7 2391,72.2 2391,73.8 2391,72.8 4405,75.4 4405,73.4 4405,72.5 4405,71.9 4405,71.4 4405,76.9 4405,78.0
4417,76.4 4417,75.4 4417,74.9 4417,74.4 4417,79.9 4417,80.9 4417,81.9 4605,85.6 4605,85.2 4605,84.7 4817,64.7
4817,62.7 4817,61.7 4817,61.2 4817,60.7 4817,65.5 4817,66.5 4817,67.5 4925,77.0 4925,76.0 4925,75.5 4925,75.0

6047,71.1 6047,72.2 6047,73.2 6604,85.3 6604,86.3 6688,78.5 6688,79.5 384,79.8 384,80.4 384,80.9 384,81.5 384,81.8
384,82.8 384,84.0 384,84.8 384,89.8 384,87.8 384,86.7 384,85.8 384,91.2 409,79.1 409,79.7 629,70.8 629,71.3 629,71.8
629,72.3 771,74.1 771,74.5 771,75.1 771,76.1 771,75.6 792,75.0 792,73.5 792,74.0 792,74.5 792,75.5 1031,77.1 1031,78.6
1031,78.1 1031,79.1 4309,73.2 4309,72.2 4309,70.2 4309,69.2 4309,68.7 4309,68.2 4489,78.2 4489,76.2 4489,75.2
4489,74.7 4489,74.2 4489,79.5 4489,80.5 4489,81.5 4566,85.4 4566,84.4 4566,83.9 4566,83.4 5273,92.1 5273,90.1
6544,77.3 6544,78.3 481,84.8 481,85.3 481,85.8 481,86.3 481,87.8 481,86.8 669,63.6 669,64.1 669,64.6 669,65.1 669,66.6
669,65.6 800,74.5 800,76.0 800,78.0 800,74.0 800,75.0 800,75.5 800,77.0 800,84.0 800,82.0 800,81.0 800,80.0 800,79.0
825,87.5 825,88.0 825,87.0 1414,74.3 1414,75.3 1414,74.8 1414,75.8 1414,76.3 1414,77.3 1414,82.3 1414,81.3 1414,80.3
1414,79.3 1414,78.3 1426,83.4 1426,83.9 1426,84.4 4303,81.2 4303,80.7 4303,80.2 4351,71.8 4351,69.8 4351,68.8
4351,68.3 4351,67.8 4351,73.7 4852,65.1 4852,64.6 4852,64.1 697,86.8 697,88.4 717,76.0 717,76.5 717,77.0 717,78.0
717,80.2 717,82.0 717,81.0 851,72.5 851,73.0 851,73.5 1231,83.5 1231,84.0 1245,71.3 1245,72.5 1245,71.8 1378,61.8
1378,62.8 1378,65.3 1378,66.9 1378,66.0 1378,71.8 1378,73.8 2210,80.4 2210,79.8 2210,80.8 2333,67.0 2333,67.5
2333,70.0 2333,68.1 2333,73.7 2333,74.7 2333,75.7 4053,76.3 4053,75.8 4053,75.3 4232,76.3 4232,75.2 4232,74.7
4232,74.2 4423,78.1 4423,77.6 4426,78.2 4426,77.7 4426,77.2 4438,77.8 4438,77.3 4438,76.8 4456,78.0 4456,77.5
4976,64.2 4976,63.6 6061,74.0 6061,75.3 6061,76.0 6075,75.4 6075,77.4 2109,75.2 2109,75.8 2109,78.2 2109,77.3
2109,76.2 4297,83.6 4297,83.0 4621,71.9 4621,71.1 4621,70.4 4621,69.9 4838,77.6 4838,76.7 4838,76.2 4838,75.6
6426,79.5 6426,80.7 6467,71.2 6467,72.3 6541,81.9 6541,82.9 6297,81.4 6297,82.5 6615,76.4 6615,77.4 6498,79.4
6498,80.4 1194,84.8 1194,85.3 1194,85.9 1194,86.8 1194,87.8 1194,88.8 1202,77.5 1202,78.5 1202,79.5 1202,80.6
1202,78.0 1202,82.9 1202,81.8 2060,73.9 2060,78.9 2060,76.9 2060,76.0 2060,74.9 4100,82.5 4100,80.5 4100,79.5
4100,79.3 4100,78.5 4100,84.7 4100,85.8 4187,66.0 4187,64.0 4187,63.0 4187,62.5 4187,62.0 4187,67.8 4187,69.8
4365,82.5 4365,81.3 4365,80.8 4365,80.3 4365,86.9 4377,70.3 4377,69.8 4377,69.3 4420,82.4 4420,81.9 4420,81.4
4764,84.7 4764,83.7 4764,82.7 6052,88.1 6052,89.0 6068,75.7 6068,76.7 6068,77.7

And finally for the CN cohort:
ID,TIME 295,84.8 295,85.3 295,85.8 295,87.0 295,87.8 295,88.8 295,90.8 295,89.9 413,76.3 413,76.8 413,77.4 413,78.5
413,79.3 413,80.3 413,85.4 413,84.3 413,83.3 413,82.3 413,81.4 413,87.4 413,89.6 559,79.3 559,81.4 559,82.3 559,79.8
559,80.4 685,89.6 685,90.1 685,90.6 685,91.7 685,92.6 685,93.6 685,94.6 685,95.6 1280,70.7 1280,71.2 1280,71.8 1280,72.7
1280,73.7 1280,74.9 1280,78.9 1280,77.7 1280,76.7 1280,75.7 1280,80.7 4213,79.0 4213,78.5 4213,78.0 4213,83.9
4213,80.0 4213,82.0 4213,86.0 5178,70.7 5178,69.1 5178,68.6 5178,72.6 5178,75.1 5230,75.8 5230,74.0 5230,73.5
5230,77.6 5230,78.6 5230,79.8 5256,72.5 5256,70.9 5256,70.4 6007,76.8 6007,77.8 6007,78.9 6009,67.5 6009,69.5
6030,65.1 6030,67.2 6053,65.7 6053,67.9 6066,68.4 6066,70.6 907,88.6 907,89.1 907,89.6 907,90.6 907,91.6 907,92.8
907,95.0 907,93.9 931,86.1 931,86.6 981,84.8 981,85.3 981,86.3 981,88.5 981,84.3 981,91.8 981,90.7 981,89.7 1021,86.9
1021,87.4 4081,74.7 4081,72.7 4119,79.3 4119,79.8 4119,80.3 4119,81.3 4119,87.5 4350,72.9 4350,73.4 4350,73.9
4350,74.9 4350,76.9 4350,80.8 4441,70.7 4441,69.9 4441,69.4 4441,68.7 4441,75.8 4555,67.8 4555,66.9 4555,66.4
4555,65.8 4644,67.6 4644,68.2 4644,68.6 4644,72.7 4644,74.7 4872,69.5 4872,69.2 4872,68.5 4900,60.8 4900,60.3
4900,59.8 4900,67.0 5154,72.4 5154,77.3 6067,63.1 6067,65.1 6260,69.0 6260,70.0 6307,76.3 6307,77.3 553,85.1 553,85.6
553,86.6 553,88.9 553,84.6 553,87.6 553,94.6 553,93.1 553,92.1 553,91.1 553,90.0 610,79.0 610,79.5 610,80.0 610,81.0
610,82.1 610,83.3 610,84.4 610,85.3 610,86.4 610,87.4 610,89.0 610,90.1 484,70.7 484,71.2 498,70.3 498,70.8 498,71.3
498,72.4 498,74.5 498,75.5 498,76.4 498,79.5 498,81.4 498,83.5 681,77.3 681,77.9 681,78.9 731,71.5 731,72.0 731,72.5
731,73.6 731,75.9 731,80.7 731,78.6 731,77.5 731,76.7 731,82.5 731,83.6 731,84.6 4150,73.9 4150,74.4 4150,75.0 4150,75.9
4150,78.1 4357,77.7 4357,75.8 4357,74.7 4357,74.2 4357,73.7 4357,79.8 4449,68.0 4449,67.4 4449,66.9 4485,77.3
4485,75.4 4485,74.3 4485,73.8 4485,73.3 4485,80.9 5153,79.0 5153,81.2 6209,72.2 6209,73.2 6234,70.6 6234,71.7
6277,70.7 6277,71.7 68,74.4 68,74.9 68,75.4 68,76.4 68,77.4 68,78.5 68,79.5 70,74.0 70,74.6 70,75.1 1206,72.9 1206,73.4
1206,74.0 1206,74.9 1206,75.9 1206,77.9 1206,79.0 1206,79.9 1222,73.4 1222,73.9 1222,74.4 1222,75.4 1222,76.4
1222,82.4 1222,80.4 1222,79.5 1222,78.6 1222,84.3 1222,86.4 4387,80.1 4387,78.1 4387,77.2 4387,76.6 4387,76.1
4387,81.9 4387,82.9 4387,83.9 4488,76.6 4488,73.6 4488,73.1 4488,72.6 4488,78.2 4488,79.2 4516,75.4 4516,73.4
4516,72.4 4516,71.9 4516,71.4 4620,81.0 4620,79.1 4620,78.1 4620,77.6 4620,77.1 4620,82.7 4620,83.8 4620,84.7
4637,74.8 4637,72.9 4637,71.8 4637,71.3 4637,70.8 4637,76.3 4637,77.3 4637,78.3 5265,77.1 5265,75.1 5265,79.2
5265,80.2 5265,81.2 6120,67.8 6120,68.8 6255,75.3 6255,76.4 751,70.9 751,71.4 751,71.9 751,72.9 751,73.9 751,78.9
751,78.0 751,76.9 751,81.8 751,82.8 751,83.8 862,73.4 862,73.9 862,74.4 862,75.4 862,76.4 4612,70.4 4612,69.5 4612,68.9
4612,68.4 4612,75.7 5125,66.2 5125,65.2 5147,76.5 5147,77.5 5176,67.8 5176,65.7 5176,70.0 67,74.5 67,75.1 67,75.5
67,76.5 419,70.2 419,70.7 419,71.2 419,72.2 419,73.2 419,74.2 419,79.2 419,77.3 419,76.4 419,75.2 419,83.1 472,72.0
472,72.5 472,73.0 472,74.0 4345,73.7 4345,71.7 4345,70.7 4345,70.2 4345,69.7 4442,75.3 4442,74.8 4442,74.2 5,73.7
5,74.2 5,74.7 5,75.7 5,76.7 8,84.5 8,85.0 16,65.4 16,65.9 16,66.4 16,67.3 16,68.4 21,72.6 21,73.6 21,74.6 21,75.6 21,77.6
21,73.1 21,82.6 21,80.6 21,79.5 21,78.6 21,84.7 23,71.7 23,72.2 23,72.7 23,73.7 23,74.7 23,76.8 23,75.9 23,79.8 23,78.8
23,77.8 4075,73.4 4075,73.9 4075,74.4 4075,75.4 4105,74.8 4105,72.8 4105,71.8 4105,71.3 4105,70.8 4105,77.0 4120,85.8
4120,83.8 4120,82.8 4120,82.3 4120,81.8 4222,86.3 4222,84.3 4222,83.4 4222,82.8 4222,82.3 6367,81.2 6367,82.2
6465,66.8 6465,67.8 637,76.1 637,76.6 637,77.1 637,78.1 637,79.2 1133,79.9 1133,80.4 1133,80.9 1133,81.9 1133,82.9
1212,75.8 1212,75.3 4026,75.5 4026,74.5 4026,74.0 4026,73.5 4545,71.0 4545,69.0 4545,68.0 4545,67.5 4545,67.0
4643,65.1 4643,65.6 4643,66.1 4643,67.1 4643,69.1 4643,70.5 4643,72.1 5157,67.5 5157,65.5 5157,71.1 5195,70.4
5195,65.1 5195,67.1 5213,65.4 5213,67.5 502,74.9 502,75.4 502,75.9 502,76.9 502,77.9 575,86.2 575,86.7 575,87.2

575,88.2 575,89.3 1035,87.7 1035,88.2 1035,89.2 1035,87.2 1035,90.2 1276,72.4 1276,73.8 4580,71.7 4580,70.7 4580,70.2 4580,69.7 4580,75.4 4616,86.8 4616,85.8 4616,85.3 4616,84.8 5137,79.9 5137,78.9 5171,74.8 5171,72.8 519,74.1 519,74.6 519,75.1 519,76.1 519,77.1 519,78.3 519,80.8 4080,79.6 4080,79.0 4080,78.6 4093,70.9 4093,70.5 4093,70.0 4401,67.5 4401,68.0 4401,68.5 4401,69.6 4401,72.0 4401,73.2 4401,75.2 4576,70.5 4576,71.0 4576,71.5 4576,72.5 4576,75.9 4576,77.1 6076,58.7 6076,59.7 6076,60.7 6145,70.3 6145,71.4 6145,72.3 6522,71.2 6522,72.2 359,81.3 359,81.8 359,82.3 359,83.3 359,84.3 359,85.7 359,91.4 359,88.6 359,86.6 538,82.7 538,83.2 538,83.7 538,84.7 538,85.7 4097,71.3 4097,70.8 4638,74.0 4638,74.7 4638,75.2 4688,82.3 4688,81.7 4951,74.9 4951,72.9 4951,72.4 4951,71.9 4951,76.1 4951,78.4 4952,69.3 4952,69.8 4952,70.3 4952,72.3 4952,75.8 43,76.2 43,76.7 43,77.2 43,79.2 43,80.2 43,78.2 369,76.0 369,76.5 369,77.0 369,78.0 369,79.1 425,85.8 425,86.3 425,86.8 425,87.8 4257,79.4 4257,78.9 4313,81.0 4313,79.0 4313,78.0 4313,77.6 4313,77.0 4313,82.9 4313,84.8 4349,73.4 4349,72.4 4349,72.0 4349,71.4 4399,81.9 4399,78.9 4399,78.4 4399,77.9 4399,83.4 4399,79.9 4399,85.5 4367,69.1 4367,67.1 4367,66.1 4367,65.6 4367,65.1 4367,70.5 4367,72.5 4835,83.2 4835,81.3 4835,80.3 4835,79.8 4835,79.3 4835,84.3 4835,85.3 4835,86.3 5242,69.3 5242,67.7 5242,67.2 97,72.8 97,73.4 97,73.8 97,74.9 1288,59.9 1288,60.5 1288,61.1 1288,62.0 1288,62.9 1288,65.0 5140,72.3 5140,70.3 5140,74.8 5140,76.8 5203,68.5 5203,66.5 5203,70.8 5203,72.0 5203,72.8 6185,82.7 6185,83.6 6185,84.6 6513,66.1 6513,67.2 159,77.9 159,78.4 159,78.9 159,79.9 159,80.9 159,81.9 159,83.0 159,86.9 159,85.9 159,84.9 159,83.9 337,75.8 337,76.3 337,76.8 337,77.8 337,78.8 337,79.8 337,80.8 337,84.8 337,82.8 337,81.8 337,86.9 337,88.9 647,72.8 647,73.3 647,73.8 647,74.9 4276,73.9 4276,74.5 4276,74.9 4276,75.9 4276,77.9 4276,79.4 4276,80.4 4335,75.7 4335,73.7 4335,72.7 4335,72.2 4335,71.7 4335,77.2 4421,75.1 4421,75.6 4421,76.1 4421,77.1 4558,71.0 4558,70.5 4558,70.0 5129,75.5 5129,74.5 5177,72.5 5177,74.5 5177,76.6 5177,77.6 5177,78.6 5194,67.2 5194,65.2 5194,69.3 5194,70.4 5194,71.3 5236,87.3 5236,85.3 5236,89.3 14,78.5 14,79.0 14,79.6 14,80.6 14,81.5 66,75.0 66,75.5 66,76.5 66,77.7 66,74.5 96,79.6 96,80.0 96,80.6 96,81.7 96,82.6 96,83.6 96,84.6 96,85.6 130,73.6 130,74.1 130,75.1 130,76.1 130,77.2 130,78.3 130,73.1 130,80.3 130,79.4 4173,74.2 4173,71.8 4173,70.8 4173,70.3 4173,69.8 4196,80.4 4196,79.4 4196,78.9 4196,78.4 4266,73.8 4266,71.0 4266,70.3 4266,69.8 4291,79.6 4291,77.6 4291,76.6 4291,76.1 4291,75.5 4320,74.7 4320,72.7 4320,71.9 4320,71.3 4320,70.7 6069,73.6 6069,74.9 6069,75.9 31,77.7 31,78.2 31,78.7 31,79.8 31,80.8 31,81.7 31,82.8 31,83.7 31,84.6 31,85.7 31,88.0 31,90.2 31,91.2 58,70.1 58,70.6 58,71.1 58,72.1 58,73.1 58,74.4 58,78.1 58,76.4 58,75.5 81,70.6 81,71.1 81,71.6 81,72.6 81,73.6 81,74.7 926,71.4 926,71.9 926,73.4 926,75.4 926,72.4 926,74.4 926,78.4 926,77.6 926,76.5 963,72.6 963,73.1 963,73.6 963,74.7 963,75.6 4020,66.5 4020,67.1 4020,67.5 4020,68.5 4020,70.5 4164,72.6 4164,73.2 4164,73.6 4164,74.7 4164,76.8 4164,78.7 4164,80.6 4448,63.8 4448,64.3 4448,64.8 4448,65.8 4448,68.0 4448,69.6 4448,71.6 6399,64.0 6399,65.1 4084,68.4 4084,68.8 4084,69.4 4084,70.4 4084,72.4 4084,74.5 4084,76.4 4158,88.3 4158,86.3 4158,85.3 4158,84.8 4158,84.3 5290,71.0 5290,67.0 5290,69.0 5290,72.1 5290,73.0 6005,67.5 6005,69.5 6184,70.5 6184,72.5 6202,69.6 6202,71.6 6385,66.6 6385,67.6 74,78.2 74,78.7 74,80.2 74,82.2 74,79.2 74,81.2 74,83.2 74,84.2 74,87.3 74,86.2 74,85.1 74,91.2 118,80.9 118,81.4 118,80.4 118,82.4 118,83.4 118,84.4 118,85.4 118,86.4 120,72.0 120,72.5 120,73.0 120,74.0 120,75.0 120,76.0 120,77.0 120,78.1 120,81.0 120,80.0 120,79.0 120,83.1 403,76.3 403,76.8 403,77.3 403,78.3 403,79.3 403,80.3 5079,73.2 5079,73.7 5079,74.3 5079,75.2 5079,77.4 5083,75.7 5083,74.8 5083,73.7 5083,77.7 5093,75.0 5093,69.3 5093,69.8 5093,70.3 5093,71.3 5093,73.3 5093,76.2 5109,80.5 5109,79.4 5109,78.9 5109,78.4 5109,82.4 5110,68.8 5110,69.9 5110,70.9 5118,70.3 5118,69.2 5118,68.2 5118,72.5 5118,74.3 5127,78.4 5127,77.3 5127,76.3 5169,67.7 5169,65.7 5169,69.7 5169,71.9 5170,76.9 5170,79.1 5170,81.0 5170,83.2 5288,81.9 5288,83.9 5288,85.9 5288,87.9 824,76.7 824,77.2 824,78.2 824,79.2 824,80.2 824,76.2 824,85.5 824,83.3 824,82.3 824,81.2 845,80.3 845,80.8 845,81.3 845,82.3 845,83.3 845,84.3 866,80.0 866,80.5 866,81.0 866,82.0 866,83.0 4279,83.7 4279,84.2 4279,84.7 4279,85.9 4384,66.4 4384,64.3 4384,63.3 4384,62.8 4384,62.3 4384,68.5 4384,69.5 4585,69.3 4585,67.3 4585,66.3 4585,65.8 4585,65.3 4585,71.0 4585,73.0 5158,76.1 5158,74.1 5158,78.6 6289,56.5 6289,57.5 618,74.8 618,75.3 618,75.8 618,76.8 618,77.8 618,78.8 618,81.8 618,80.8 618,79.8 618,88.0 4021,66.5 4021,67.0 4021,67.5 4021,68.5 4021,70.1 4021,73.9 4021,74.9 4032,72.2 4032,71.2 4032,70.7 4032,70.2 4496,76.1 4496,76.6 4496,77.1 4496,78.1 95,71.1 95,71.6 95,72.1 479,73.5 479,74.0 479,74.5 479,75.5 479,77.6 479,76.6 479,79.8 479,78.8 677,70.8 677,71.4 677,71.8 677,74.8 677,73.8 677,80.8 677,78.0 677,77.0 677,76.0 677,81.8 677,72.9 677,83.8 4348,66.6 4348,66.1 4386,88.7 4386,86.7 4386,85.7 4386,85.1 4386,84.7 4429,77.0 4429,77.5 4429,78.0 4429,79.0 4429,81.1 4429,82.8 4921,67.7 4921,67.2 4921,66.7 5263,77.4 5263,75.2 5289,63.2 5289,59.7 5289,60.2 5289,61.7 5289,64.2 5289,65.2 6211,80.6 6211,81.8 6293,86.3 6293,87.3 516,87.6 516,88.1 516,88.6 516,89.6 516,90.6 734,72.6 734,73.1 734,73.6 734,74.6 734,75.6 734,76.6 734,81.6 734,79.6 734,78.6 734,77.6 734,83.8 734,84.8 923,83.1 923,83.6 923,84.1 923,85.1 923,86.1 923,87.1 923,91.1 923,90.1 923,89.1 923,88.1 1016,78.3 1016,78.8 1016,79.3 1016,80.4 1016,81.4 1016,82.3 1016,86.3 1016,85.3 1016,84.3 1016,89.4 1016,91.3 1086,81.4 1086,81.9 1086,82.4 1086,83.4 4176,88.3 4176,86.0 4176,85.0 4176,84.5 4176,84.0 4176,90.3 4176,91.4 4176,92.4 4177,86.9 4177,85.9 4177,85.4 4177,84.9 4177,91.3 4177,93.4 4179,87.3 4179,85.0 4179,84.0 4179,83.5 4179,83.0 4179,89.4 4505,80.4 4505,80.9 4505,81.4 4505,82.4 4505,84.4 4508,78.1 4508,77.6 4508,77.1 5198,71.2 5198,69.2 5198,73.6 5198,74.6 5198,75.6 5259,80.8 5259,78.8 5259,83.1 5259,84.0 5259,85.0 6298,69.5 6298,70.5 6352,71.4 6352,72.4 6572,67.9 6572,68.9 48,78.3 48,78.8 48,79.4 48,80.3 48,81.4 4082,79.4 4082,77.4 4082,76.5 4082,75.8 4082,75.4 4464,74.7 4464,72.3 4464,71.4 4464,70.9 4464,70.4 4464,76.3 4785,68.9 4785,66.7 4785,71.1 6488,76.6 6488,77.6 576,77.5 576,78.0 576,78.5 576,79.5 672,62.0 672,62.5 672,63.0 672,64.1 672,65.0 672,66.3 813,72.7 813,73.3 813,73.7 813,74.8 813,75.8 813,76.8 1023,77.1 1023,77.6 1023,78.2 1023,79.1 1023,80.1 1023,83.1 4389,83.1 4389,82.1 4389,81.6 4389,81.1 4389,86.9 4491,88.1 4491,86.1 4491,85.1 4491,84.6 4491,84.1 4491,90.2 4878,72.9 4878,73.4 4878,73.9 4878,74.9 5271,80.4 5271,83.4 5283,72.9 5283,75.9 6088,69.2 6088,70.2 6316,77.5 6316,78.7 303,84.2 303,86.2 303,84.7 303,85.2 303,87.3 303,88.4 303,94.2 303,92.2 303,91.2 303,89.3

303,95.1 327,70.0 327,70.5 327,72.0 327,71.0 327,73.1 4028,63.5 4028,64.0 4028,64.5 4028,65.5 4028,67.5 4028,70.0 4028,72.0 4308,74.0 4308,75.0 4308,76.1 4308,78.0 4308,79.5 4410,69.1 4410,69.6 4410,70.1 4410,71.1 4410,73.1 4410,74.2 5126,70.5 5126,69.5 5126,68.5 5126,74.9 5222,67.5 5222,69.5 5222,73.6 6031,67.2 6031,69.4 6187,74.3 6187,75.5 6204,65.3 6204,66.5 125,73.7 125,74.2 125,74.7 125,75.7 125,76.7 125,77.8 262,85.8 262,86.4 262,86.8 262,87.8 262,88.8 262,89.8 1002,76.3 1002,76.8 1002,77.3 1002,78.4 1002,79.4 4014,84.7 4014,82.7 4014,81.7 4014,81.2 4014,80.7 4037,75.6 4037,76.2 4037,76.6 4037,77.6 4037,79.6 4037,81.7 4060,88.4 4060,86.4 4060,85.4 4060,84.9 4060,84.4 4200,74.2 4200,72.2 4200,71.2 4200,70.7 4200,70.2 4200,76.2 4200,78.3 4427,73.3 4427,72.3 4427,71.8 4427,71.3 4427,77.0 4427,75.4 4427,79.0 5078,69.4 5078,68.4 5078,67.9 5078,67.4 5078,71.8 5078,73.0 5082,71.2 5082,70.2 5082,69.7 5082,69.2 5100,73.2 5100,72.2 5100,71.7 5100,71.2 5100,75.5 5100,76.5 5100,77.5 5131,72.5 5131,71.5 5131,70.5 5141,76.7 5141,77.7 5141,80.8 5244,77.8 5244,75.8 5253,68.1 5253,70.1 5253,72.2 5253,74.2 6136,61.1 6136,63.1 6159,76.2 6159,78.2 6192,83.3 6192,85.3 6401,75.7 6401,76.6 5294,68.5 5294,71.0 951,84.8 951,85.3 951,85.8 951,88.1 951,88.9 951,89.8 951,86.8 951,90.4 1250,73.2 1250,76.5 1250,77.3 1250,73.7 1250,74.1 1250,75.3 1250,79.3 1251,74.2 1251,74.7 1251,75.2 1251,76.3 1251,77.4 4578,69.2 4578,69.7 4578,70.2 4578,71.2 4578,73.2 5202,68.0 5202,65.7 5272,69.9 5272,71.9 5272,75.1 5287,78.9 5287,80.9 5296,69.3 5296,71.3 5296,74.2 643,71.4 643,74.4 643,71.9 643,72.4 643,73.4 818,74.0 818,74.5 818,75.0 818,76.0 818,77.0 934,70.0 934,70.5 934,71.0 934,72.0 934,73.0 934,75.0 934,79.0 934,77.0 934,76.0 934,81.8 5292,74.3 5292,76.3 5292,79.1 5292,80.1 5295,75.5 5295,77.5 578,77.0 578,77.5 578,78.0 578,79.0 768,77.3 768,77.8 768,79.3 768,78.3 1099,79.2 1099,79.7 1099,80.2 19,73.1 19,73.7 19,74.1 19,76.1 177,74.8 177,75.5 177,75.8 177,76.9 257,78.5 257,79.1 257,79.5 257,80.7 257,81.5 257,82.5 257,86.5 257,85.5 257,84.5 5159,68.4 5159,66.4 473,72.6 473,73.2 473,73.6 473,74.6 473,75.6 473,76.6 473,82.6 473,79.6 473,78.6 473,77.5 473,84.1 473,86.2 4174,74.9 4174,74.2 4174,73.7 4340,66.7 4340,67.7 4340,70.5 4340,72.3 4340,74.6 4424,69.6 4424,67.0 4424,66.2 4424,71.8 4856,68.1 4856,66.0 4856,65.5 4856,65.0 4856,70.7 5040,75.9 5040,77.0 5040,79.1 5040,81.3 315,71.5 315,72.0 315,72.6 315,74.6 315,75.5 315,79.5 315,78.5 315,77.5 315,76.5 4103,72.9 4103,71.9 4103,71.4 4103,70.9 4391,74.4 4391,74.9 4391,75.4 4391,76.4 89,65.1 89,65.7 89,66.1 89,67.1 89,68.1 89,69.8 89,74.1 89,72.9 89,71.8 89,70.8 311,78.6 311,78.1 311,79.0 311,80.1 311,82.1 311,85.2 311,84.4 311,83.1 312,82.8 312,83.3 312,83.7 386,72.3 386,72.9 386,74.3 386,73.3 386,75.3 386,76.3 4155,79.1 4155,78.1 4155,77.6 4382,75.9 4382,76.3 4382,76.9 4382,77.9 4393,73.5 4393,73.9 4393,74.4 4393,75.5 4393,77.6 4552,67.4 4552,65.3 4552,64.3 4552,63.7 4552,63.2 4559,71.7 4559,68.5 4559,68.0 4559,67.4 4559,69.4 4739,67.2 4739,66.0 4739,65.5 4739,65.2 4762,75.8 4762,74.9 4762,74.4 4762,73.8 4795,63.2 4795,62.2 4795,61.2 5023,63.9 5023,64.9 5023,65.9 5167,66.6 5167,68.6 304,70.8 304,72.8 1256,71.6 1256,72.2 1256,72.6 1256,73.7 1256,75.8 4090,72.4 4090,71.9 4090,71.4 4090,73.5 4208,78.2 4208,78.7 4208,79.2 4208,80.2 4224,76.1 4224,75.6 4224,75.1 4224,81.5 4224,82.8 4339,86.3 4339,85.3 4339,84.8 4339,84.3 4428,73.6 4428,73.1 4428,72.6 4428,78.5 4428,80.2 5278,80.2 5278,84.7 5282,66.9 5282,69.1 5282,71.6 5282,72.7 489,73.9 489,74.4 526,85.1 526,85.8 526,86.1 526,87.1 526,88.1 692,76.7 692,77.1 692,77.7 692,78.8 711,77.8 711,79.3 711,77.3 711,78.3 711,80.3 1267,73.2 1267,73.7 1267,74.2 1267,75.5 1267,76.2 2201,63.7 2201,64.2 2201,64.7 2201,65.7 2201,66.7 2201,67.7 2201,70.4 4234,74.6 4234,72.1 4234,71.1 4234,70.6 4234,70.1 4503,75.7 4503,73.8 4503,72.7 4503,72.2 4503,71.7 4560,70.3 4560,70.8 4560,71.2 4560,72.3 4649,69.4 4649,67.4 4649,66.4 4649,65.9 4649,65.4 4649,71.0 171,78.3 171,78.7 171,79.3 171,82.3 171,80.3 171,81.3 171,84.5 171,83.4 172,70.6 172,71.2 172,71.6 172,72.6 172,73.6 172,74.6 172,75.6 172,76.6 4003,77.3 4003,75.3 4003,74.4 4003,73.3 4003,72.8 4003,72.3 4003,78.9 4003,80.9 4018,76.1 4018,76.6 4018,77.1 4018,78.0 4018,80.1 40,73.2 40,73.6 40,75.2 40,76.3 40,74.2 90,69.8 90,70.3 90,70.8 90,71.9 90,72.8 352,77.0 352,77.5 352,78.0 352,79.0 352,81.0 352,80.0 352,85.0 352,84.0 352,83.0 352,82.0 533,81.5 533,82.0 533,81.0 533,83.0 533,84.0 534,62.8 534,63.3 534,63.8 534,64.9 534,65.9 4076,72.6 4076,73.2 4076,73.6 4076,74.7 4076,76.6 4076,78.9 4076,81.0 4086,85.8 4086,83.9 4086,82.8 4086,82.4 4086,81.8 4086,88.1 4104,74.5 4104,73.4 4104,72.9 4104,72.4 6016,65.4 6016,67.4 6025,79.7 6025,82.1 6038,77.4 6038,78.4 6038,79.6 6097,74.6 6097,76.6 6175,70.5 6175,69.3 6396,75.3 6396,76.3 15,80.8 15,81.8 15,83.8 15,81.3 15,82.8 35,76.9 35,77.4 35,77.9 35,79.9 35,78.9 47,84.7 47,85.2 47,85.7 47,87.7 47,86.7 47,89.7 1286,75.5 1286,77.5 1286,78.1 1286,79.5 1286,76.0 1286,76.5 1286,85.4 1286,82.5 1286,80.5 1286,86.4 4469,66.1 4469,66.6 4469,67.1 4469,68.1 4469,71.6 4469,73.6 5091,68.0 5091,70.0 5091,72.7 5091,74.7 5102,68.4 5102,66.4 5280,69.5 5280,67.5 6164,68.9 6164,70.9 6273,65.8 6273,67.2 6493,79.5 6493,80.5 967,76.8 967,77.3 967,77.8 967,78.8 1013,77.8 1013,78.3 1014,84.7 1014,85.2 1014,85.7 1014,86.7 601,76.6 601,77.1 601,77.6 601,78.6 601,79.6 6251,65.5 6251,66.7 382,75.5 382,76.0 382,76.5 382,77.5 382,78.5 382,79.7 382,84.5 382,82.5 382,81.5 382,80.5 382,87.7 648,72.0 648,74.5 648,71.5 648,72.5 648,73.5 657,77.7 657,78.2 657,79.7 657,78.7 657,80.7 657,81.7 1232,72.1 1232,72.6 1232,73.1 1232,74.1 1232,75.1 1232,76.1 1232,81.1 1232,79.1 1232,78.1 1232,77.1 4010,73.8 4010,71.8 4010,71.3 4010,70.8 4043,82.1 4043,82.6 4043,83.1 4043,84.1 4043,86.1 4043,88.8 4043,90.8 4453,65.9 4453,66.4 4453,66.9 4453,67.9 4453,70.1 4453,71.9 4453,73.9 4483,73.6 4483,71.5 4483,70.7 4483,70.0 4483,69.5 4483,75.6 6133,69.3 6133,70.4 6133,71.3 72,70.6 72,71.1 72,71.6 72,72.6 72,74.6 72,75.6 72,73.7 72,78.6 72,77.6 72,76.6 72,82.4 72,84.3 113,75.2 113,75.7 113,76.2 113,77.2 113,79.3 113,80.2 113,78.2 113,85.4 113,83.2 113,82.2 113,81.2 298,76.4 298,76.9 298,77.4 298,78.4 298,79.4 298,80.4 298,81.4 298,86.6 298,84.4 298,83.4 298,82.4 298,87.9 6118,74.7 6118,76.7 405,76.1 405,76.6 5243,73.0 5243,71.0 5243,76.0 5243,77.0 260,78.6 260,79.1 260,79.6 260,80.6 260,81.6 260,82.6 260,83.6 260,87.6 260,86.6 260,85.6 260,84.6 684,77.4 684,77.9 684,78.4 4148,77.0 4148,75.0 4148,74.0 4148,73.5 4148,73.0 4148,78.9 4148,80.9 4604,69.0 4604,66.9 4604,66.0 4604,65.5 4604,65.0 4604,70.3 4604,72.4 4645,80.3 4645,78.3 4645,77.3 4645,76.8 4645,76.3 4645,82.4 4843,75.4 4843,74.5 4843,73.4 5185,71.9 5185,69.9 5185,74.1 5185,75.1 5185,76.1 5200,78.4 5200,76.4 5200,80.4 5200,81.4 5200,82.4 5228,77.8 5228,79.8 5228,82.0 5228,84.0 5266,65.9 5266,67.9 5266,69.9 5266,71.0 6024,67.4 6024,69.4 6147,69.6 6147,71.6 6168,76.0 6168,78.0 6330,70.2

6330,71.2 6436,69.8 6436,70.8 245,73.8 245,74.3 272,70.4 272,70.9 272,71.4 272,72.4 272,74.8 272,73.6 272,80.4 272,78.5 272,77.5 272,76.4 272,82.4 500,77.4 500,77.9 500,78.4 500,79.4 863,79.1 863,79.6 863,83.2 863,80.1 863,82.4 863,84.1 1242,71.1 1242,71.6 1242,72.2 1242,77.4 1242,76.4 4586,75.8 4586,76.4 4586,76.8 4586,77.9 4586,79.8 4599,61.9 4599,60.9 4599,60.4 4599,59.9 4609,82.2 4609,81.7 4832,71.9 4832,70.2 4832,69.7 4369,68.3 4369,69.0 4369,69.3 4369,70.3 4369,73.8 4369,75.8 4371,69.7 4371,68.7 4371,68.3 4371,67.7 4396,80.4 4396,79.4 4396,78.4 4396,84.4 6146,65.5 6146,67.5 6228,75.1 6228,77.1 6288,72.6 6288,73.6 6452,62.3 6452,63.3 6621,57.4 6621,58.4 886,71.3 886,71.8 886,72.3 886,73.3 886,74.3 886,76.3 969,70.0 969,70.5 969,71.0 969,73.0 969,72.0 969,74.1 969,79.1 969,77.0 969,76.1 969,80.5 4343,81.6 4343,80.7 4343,80.2 4343,79.6 4343,85.0 4352,83.6 4352,84.0 4352,84.5 4352,85.6 4352,90.1 5142,78.3 5142,77.3 5142,76.3 5175,79.5 5175,83.5 5175,81.5 5258,79.9 5258,77.9 5258,83.8 6027,75.6 6027,77.6 6037,67.0 6037,69.0 6105,75.4 6105,77.4 319,70.2 319,70.7 441,73.7 441,74.7 441,75.7 441,76.7 441,72.7 441,73.4 441,77.7 441,82.7 441,79.7 441,78.7 1301,72.1 1301,72.6 1301,73.2 1301,74.1 1301,75.1 433,85.5 433,86.0 433,86.5 433,88.5 433,87.5 488,70.9 488,71.4 488,72.0 488,73.9 488,72.9 493,77.5 493,78.0 493,80.5 493,79.5 493,78.6 525,70.0 525,70.6 525,71.1 525,73.0 525,72.1 4598,65.1 4598,65.6 4598,66.1 4598,67.1 4598,69.1 4598,70.4 4598,72.4 5113,65.1 5113,66.1 5113,67.1 5113,69.3 5113,71.4 5269,66.9 5269,64.9 6104,69.0 6104,70.0 6104,71.0 6359,67.4 6359,68.5 6360,68.2 6360,69.2 6509,65.4 6509,66.4 6510,66.9 6510,67.9 86,80.3 86,80.8 86,84.4 86,81.3 86,82.3 86,83.3 184,78.3 184,79.3 184,80.3 184,81.3 184,78.7 186,80.4 186,80.9 186,81.4 186,82.4 186,83.5 186,84.6 186,88.4 186,87.0 186,85.6 196,78.5 196,79.0 196,81.0 196,78.0 196,80.0 4269,67.6 4269,66.3 4269,65.6 283,78.4 283,78.9 283,79.4 283,80.4 283,81.4 301,73.4 301,73.9 301,74.4 301,75.4 301,76.4 301,81.4 301,80.4 301,79.4 301,78.4 459,72.9 459,73.4 459,73.9 459,74.9 459,75.9 686,72.0 686,72.5 686,73.0 686,74.0 686,75.0 4466,83.8 4466,81.8 4466,80.8 4466,80.3 4466,79.8 4466,85.6 4482,81.2 4482,79.2 4482,78.2 4482,77.7 4482,77.2 4482,82.8 4520,67.8 4520,68.3 4520,68.8 4520,69.8 4520,71.8 4520,73.5 4520,75.5 4587,69.7 4587,67.7 4587,66.7 4587,66.2 4587,65.7 4632,71.7 4632,69.7 4632,68.7 4632,68.2 4632,67.7 726,81.0 726,81.5 726,82.2 767,73.0 767,73.5 767,74.0 767,75.0 767,77.1 767,80.1 767,79.0 767,78.0 767,83.6 767,85.6 810,83.3 810,83.8 810,84.3 810,85.9 1094,76.0 1094,76.6 1094,77.1 6008,63.1 6008,65.1 6015,68.3 6015,70.3 6116,69.6 6116,71.8 6178,66.0 6178,67.0 6178,68.0 4125,79.8 4125,76.8 4125,76.4 4125,75.8 4139,71.0 4139,70.5 4151,74.2 4151,73.2 4151,72.7 4151,72.2 4372,72.0 4372,71.0 4372,70.5 4372,70.0 6051,66.3 6051,68.5 6059,64.9 6059,66.9 6062,67.4 6062,69.6 6065,71.2 6065,73.3 6085,55.8 6085,58.0 6151,65.1 6151,67.2 6157,67.8 6157,69.8 1195,76.9 1195,77.6 1195,78.9 1195,79.9 1195,80.9 1195,85.9 1195,84.0 1195,82.9 1195,82.0 1195,88.0 1197,82.4 1197,83.7 1197,82.9 1197,84.4 4255,74.4 4255,72.9 4292,75.5 4292,71.9 4292,71.4 4292,70.9 4292,76.5 4292,72.7 4292,78.5 4376,76.5 4376,77.0 4376,77.5 4376,78.7 4376,80.8 4376,83.0 4376,84.0 5124,76.7 5124,77.7 5124,78.7 5124,81.4 5193,74.5 5193,72.5 5193,76.5 5193,78.5 6054,78.6 6054,80.6 6058,68.3 6058,70.3 6080,76.8 6080,78.8 6094,69.6 6094,71.6 6333,77.9 6333,78.9 6575,73.5 6575,74.5 6580,79.9 6580,80.9

## 5. Results for the gray matter density maps

This secondary analysis is done on the same cohorts from ADNI. Table 1 recalls the demographics and amount of available scans for each model.

| | AD cohort | | Aβ+ MCI cohort | | Healthy cohort | |
|---|---|---|---|---|---|---|
| | Male | Female | Male | Female | Male | Female |
| Patients ($N$) | 329 | 261 | 262 | 176 | 210 | 264 |
| Visits ($N_{scan}$) | 1,447 | 1,092 | 1,164 | 794 | 1,063 | 1,169 |
| Total follow-up (y) | 3.7 ± 2.9 | 3.4 ± 3.0 | 4.5 ± 3.4 | 4.6 ± 3.3 | 4.6 ± 3.3 | 4.1 ± 3.0 |
| Age at baseline | 75.0 ± 7.1 | 73.6 ± 7.7 | 75.4 ± 7.5 | 71.5 ± 7.8 | 74.0 ± 6.1 | 72.7 ± 6.0 |
| Education (y) | 16.1 ± 2.8 | 14.7 ± 2.6 | 16.4 ± 2.8 | 15.5 ± 2.7 | 17.2 ± 2.4 | 16.1 ± 2.8 |
| APOE-ε4 (2/1/0) | 69/163/97 | 44/133/84 | 32/99/121 | 24/71/81 | 5/51/154 | 7/75/182 |
| MMSE | 24.0 ± 4.0 | 23.2 ± 4.6 | 27.7 ± 2.0 | 28.2 ± 2.1 | 29.0 ± 1.2 | 29.2 ± 1.1 |
| ADAS-Cog13 | 26.5 ± 10.6 | 29.2 ± 11.8 | 15.3 ± 6.9 | 12.6 ± 6.7 | 10.0 ± 4.6 | 8.3 ± 4.3 |

Table 1: Demographics for cohorts selected from ADNI. Numeric fields are in the form mean ± standard deviation. (y) is years. (2/1/0) refers to the amount of APOE-ε4 alleles. Cognitive scores are for all visits (not just baseline).

Here we report the figures for loss of grey matter in cortical regions and subcortical volumes defined by the AAL2 atlas. Since the AAL2 atlas is a volumetric atlas, data cannot be projected onto a reference cortical surface and make visualization more tedious. We used the fsleyes software to display the lateral views, but could not provide a medial view. Significant associations for the AD cohort are reported in Fig. 9, for CN cohort in Fig. 10 and for MCI cohort in Fig. 11. We use the same conventions for plotting the signed log $p$-values as in the main manuscript. Table 2 reports the associations found for subcortical atrophy.

In order to facilitate comparison with the main analysis, we present results with the same structure and emphasize the differences at the end of each paragraph.

*Impact of sex on cortical thinning for AD progression.* Almost all regions of the cortex display a significantly higher acceleration factor for female patients, with an emphasis on the entire temporal, frontal and occipital lobes and the anterior parietal lobe. Onset ages are, on the other hand, more homogeneous across sexes. The motor cortex, the cingulate gyrus and the medial parietal lobe display an earlier onset for men, while the inferior occipital lobe displays an earlier onset age for female patients. The impact of sex on loss of gray matter is thus almost identical to that of sex on cortical thinning for AD patients.

*Impact of APOE-ε4 genotype on cortical thinning for AD progression.* With the exception of the postcentral gyri, almost all regions of the cortex also display a significantly higher acceleration factor for APOE-ε4 carriers. The effect is overall lower than that of the influence of sex. The temporal lobe, the parietal lobe and the frontal lobe also display an earlier onset for APOE-ε4 carriers. Once again, the results are similar to those of the main analaysis.

*Impact of sex on cortical thinning for healthy aging.* Contrary to what was seen in AD progression, the sexual dimorphism for healthy aging manifests mainly through a significantly earlier onset age for male subjects, especially in the motor cortex, but also in the parietal and frontal lobes. On the other hand, acceleration factors are not statistically distinguishable across sexes except for a few isolated regions that barely display threshold-level of significance. The findings for the onset age are in line with the main analysis, but the pace of loss of gray matter slightly differs, possibly because of the small effect sizes.

*Impact of APOE-ε4 genotype on cortical thinning for healthy aging.* APOE-ε4 allele carriers do not display significantly different patterns for loss of gray matter in the cortex across healthy aging, in line with the findings on cortical thinning.

*Influence of both covariates for patients with MCI.* The onset ages display correlations with sex that are similar to those displayed for healthy aging and correlations with APOE-ε4 status that are similar to those displayed for AD progression, although of a weaker effect. On the other hand, the acceleration factors are significantly higher for men in almost all the cortex, and significantly higher for APOE−ε4 carriers in the temporal, frontal and parietal lobes. The associations with onset are in line with the main analysis, but the associations with pace of loss of gray matter are novel.

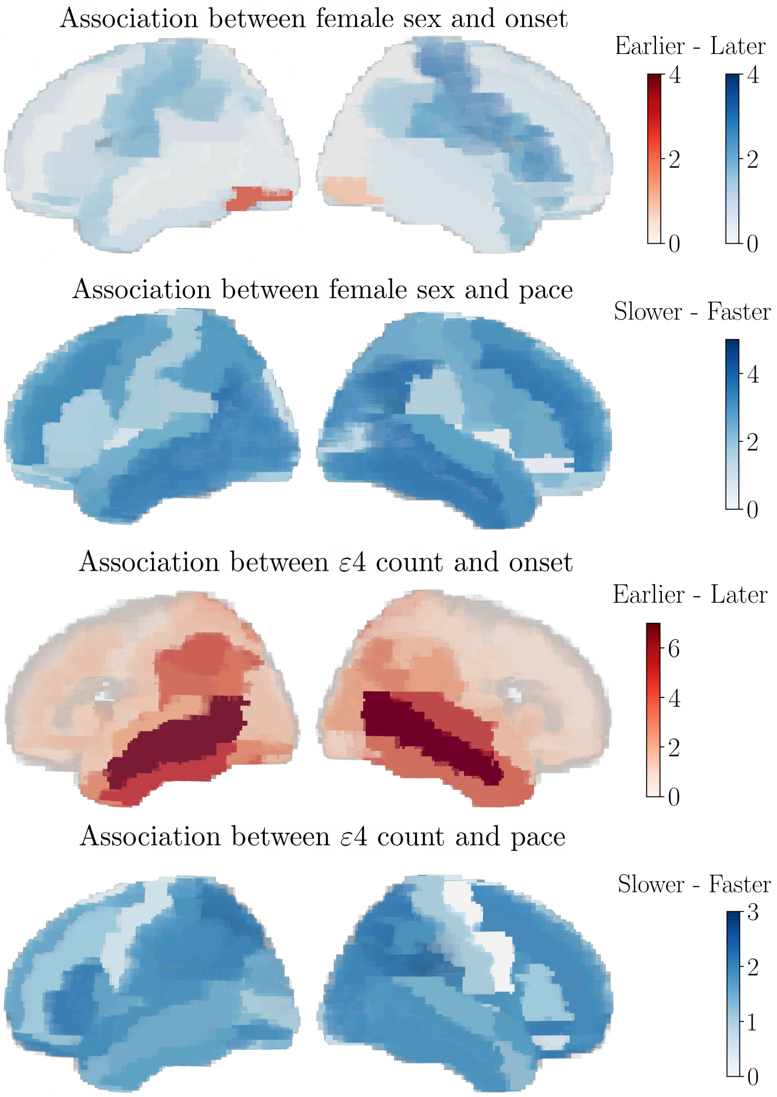


Figure 9: Cortical thinning over the course of AD progression. Columns display left lateral and right lateral view. Legend bars shows negative log $p$-values. It should be noted that blue values indicate that the considered stratifying factor is a protective factor for onset age but a risk factor for accelerations regarding disease severity, since one wants the highest possible onset and lowest possible acceleration factor.

*Correlations with the patterns of atrophy for subcortical structures.* For healthy aging, male sex correlates with an earlier onset age for most regions with no significant differences in pace of atrophy, while no significant correlation in found for APOE-$\varepsilon$4 genotype. For the AD cohort on the other hand, female sex correlates with an earlier onset for most regions, and higher paces of the hippocampi, parahippocampi, and bilateral amygdalas and putamens while APOE-$\varepsilon$4 genotype correlates with earlier onset and higher pace for the hippocampi, parahippocampi, bilateral amygdalas and left thalamus. We reach the same conclusion regarding the MCI cohort as for the main analysis : sex correlates with loss of gray matter in a similar fashion to the healthy cohort, while APOE-$\varepsilon$4 genotype correlates in a similar fashion to the AD cohort.

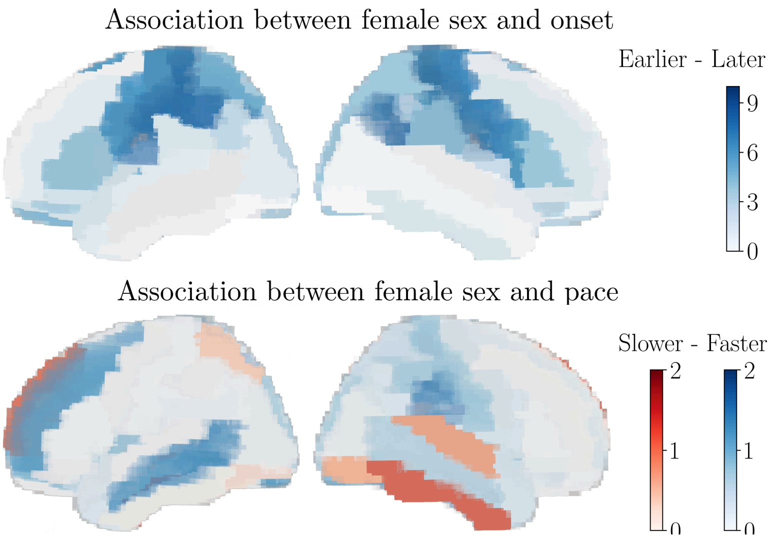


Figure 10: Cortical thinning over the course of healthy aging.

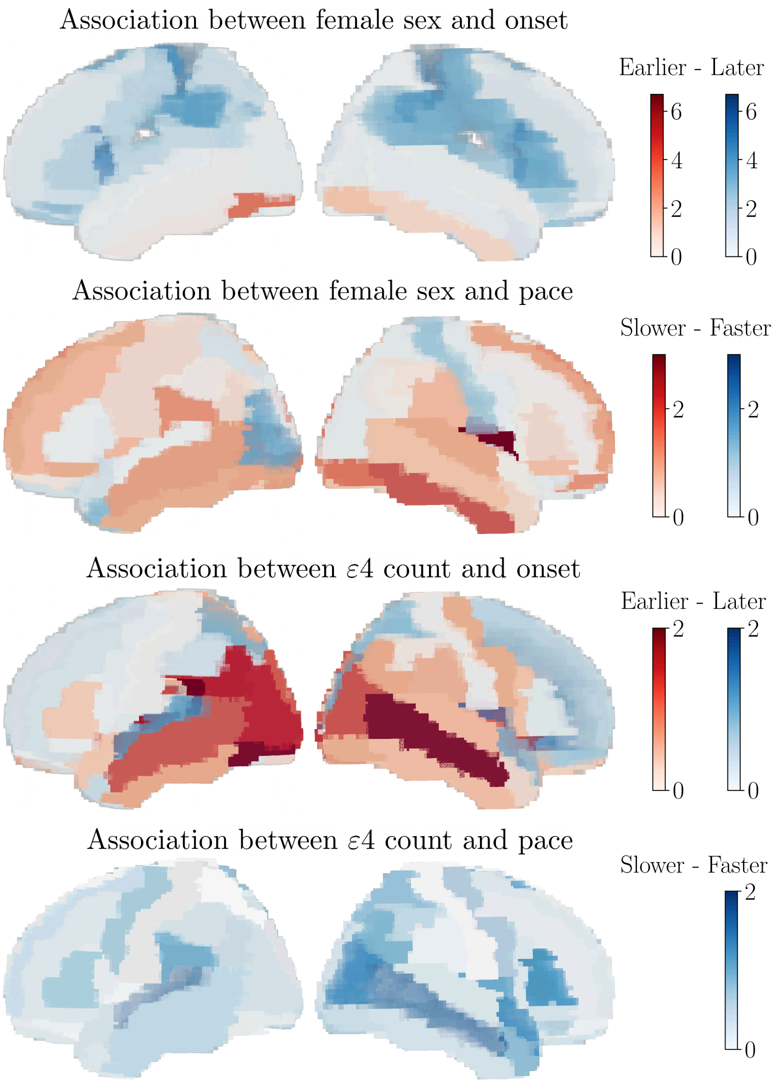


Figure 11: Cortical thinning for patients with MCI.

| | | Correlations with sex | | | Correlations with APOE-$\varepsilon$4 | | |
|---|---|---|---|---|---|---|---|
| | | $p_{val}$ CN | $p_{val}$ MCI | $p_{val}$ AD | $p_{val}$ CN | $p_{val}$ MCI | $p_{val}$ AD |
| Amygdala (L) | Onset | - | - | - | - | 4.8e-02 | 3.7e-07 |
| | Pace | - | - | 4.0e-06 | - | 1.0e-04 | 2.8e-08 |
| Amygdala (R) | Onset | - | - | - | - | 4.9e-02 | 2.8e-08 |
| | Pace | - | - | 2.0e-06 | - | 1.4e-04 | 1.6e-06 |
| Caudate (L) | Onset | 3.7e-09 | 9.5e-03 | 2.0e-03 | - | - | - |
| | Pace | - | - | - | - | - | - |
| Caudate (R) | Onset | 1.2e-09 | 4.3e-3 | 1.5e-04 | - | - | - |
| | Pace | - | - | - | - | - | - |
| Hippocampus (L) | Onset | 4.8e-06 | - | - | - | 1.8e-02 | 6.4e-08 |
| | Pace | - | - | 1.3e-02 | - | 6.8e-04 | 2.7e-06 |
| Hippocampus (R) | Onset | 1.2e-03 | - | - | - | 3.9e-02 | 8.5e-07 |
| | Pace | - | - | 1.7e-03 | - | 2.9e-04 | 5.0e-07 |
| ParaHippocampus (L) | Onset | - | - | - | - | - | 5.6e-04 |
| | Pace | - | - | 1.2e-02 | - | 1.9e-02 | 7.5e-04 |
| ParaHippocampus (R) | Onset | - | - | - | - | - | 1.5e-05 |
| | Pace | - | - | 1.9e-02 | - | 1.3e-02 | 2.0e-04 |
| Putamen (L) | Onset | 1.9e-07 | 1.8e-02 | 1.0e-03 | - | - | - |
| | Pace | - | - | 1.2e-02 | - | - | - |
| Putamen (R) | Onset | 3.3e-07 | 1.3e-02 | 1.2e-04 | - | - | - |
| | Pace | - | - | 8.3e-3 | - | - | - |
| Thalamus (L) | Onset | 6.3e-09 | 1.3e-05 | 4.5e-05 | - | - | 2.2e-02 |
| | Pace | - | - | - | - | - | 8.9e-04 |
| Thalamus (R) | Onset | 7.0e-08 | 1.6e-05 | 6.3e-04 | - | - | - |
| | Pace | - | - | - | - | - | - |

Table 2: Significant correlations with subcortical structures' atrophic dynamics. For all the significant features, APOE-$\varepsilon$4 genotype are correlated with lower onset age and higher pace, and female sex is correlated with higher onset age and higher pace, although correlations with pace are rare and only present in AD and MCI cohorts. Pallidum is discarded as it shows no significant correlation.
